# Does socioeconomic position moderate the associations between the content and delivery features of digital behaviour change interventions for smoking cessation and intervention effectiveness? A systematic review and meta-analysis

**DOI:** 10.1080/17437199.2024.2366189

**Published:** 2024-06-18

**Authors:** Corinna Leppin, Tosan Okpako, Jamie Brown, Claire Garnett, Olga Perski

**Affiliations:** a Department of Behavioural Science and Health, University College London, London, UK; b School of Psychological Science, University of Bristol, Bristol, UK; c Herbert Wertheim School of Public Health and Human Longevity Science, University of California San Diego, San Diego, CA, USA; d Faculty of Social Sciences, Tampere University, Tampere, Finland

**Keywords:** Smoking cessation, health equity, digital behaviour change intervention, mHealth, meta-CART, meta-analysis

## Abstract

Prior research indicates that digital smoking cessation interventions can be effective, but little is known about their active ingredients. Therefore, this review aimed to examine the associations of content (behaviour change techniques [BCTs]), delivery features (delivery mode, readability, ease-of-use), and socioeconomic position with effectiveness. Systematic searches and hand searches were conducted from February to June 2023 to identify experimental evaluations of digital smoking cessation interventions published since 2004. Random-effects meta-analyses were used to explore intervention effectiveness. Meta-CART were used to explore whether content, delivery features, or socioeconomic position moderate effectiveness and assessed interactions between potential moderators. Meta-regressions were performed as sensitivity checks. For *k* = 29 studies (*n* = 42,662), the authors provided sufficient data and materials for inclusion in the primary analyses. Participants in the intervention groups had greater odds of successfully quitting smoking (OR = 1.29, 95% CI: 1.10–1.51, *p *= .002) with similar effect sizes across socioeconomic groups (OR_low SEP_* *= 1.25, 95% CI: 1.00–1.57, *p *= .048; OR_high SEP_* *= 1.36, 95% CI: 1.06–1.76, *p *= .017). No delivery features were significantly associated with effectiveness. The BCT ‘commitment’ was associated with larger effects in populations with high, but not low, socioeconomic positions. There were no significant interactions between potential moderators. Digital smoking cessation interventions are effective across socioeconomic groups. Uncertainty around active ingredients remains.

## Introduction

Smoking is still a key contributor to worldwide morbidity and mortality (Department of Health and Social Care, 2017; Lewer et al., 2020; Marteau et al., 2021; NHS Digital, 2020; Office for National Statistics, 2022). Socioeconomic differences in the success rates of quit attempts play a crucial role in maintaining the social gradient in smoking (Hiscock et al., 2012; Kotz & West, 2009; Marteau et al., 2021), which, in turn, contributes to stark inequalities in health outcomes and life expectancy (Lewer et al., 2020). Individual-level, behavioural interventions with and without pharmacological support have historically been unable to compensate for these inequalities in quit success (T. Brown et al., 2014; Hill et al., 2014). However, novel and improved smoking cessation interventions, especially for disadvantaged groups, are still needed to achieve the UK government target of ‘Smokefree 2030’ (i.e., a smoking prevalence of under 5% by the year 2030) for all groups (Hopkinson, 2020), even if all of the government’s recently announced planned policies are implemented (Department of Health and Social Care, 2023).

Digital behaviour change interventions (DBCIs) are services or products aimed at changing behaviours, such as smoking, delivered through computer or mobile technologies, such as mobile phones, smartphone applications, websites, or wearable devices (West & Michie, 2016). They have the potential to fill treatment gaps (Abernethy et al., 2022) as they do not present the same barriers as other treatment modalities, such as face-to-face behavioural support (Kale et al., 2019; Kwah et al., 2019). Barriers to face-to-face support include a lack of time, monetary, and childcare resources to attend inflexible appointments (Kale et al., 2019; Kwah et al., 2019). Since DBCIs can be used remotely and asynchronously, they do not present these same barriers. DBCIs can also be more discrete than other intervention modalities, potentially preventing barriers related to shame, lack of social support, and privacy concerns (Kale et al., 2019; Kwah et al., 2019). Additionally, there is considerable interest in DBCIs by healthcare providers and the National Health Service (NHS) due to their potential reach and scalability (Abernethy et al., 2022; NHS, 2019). While there is a plentiful supply of DBCIs for smoking cessation, most are not evidence-informed and few have been formally evaluated, and may therefore be inadequate to address the complexity of smoking behaviour (Abernethy et al., 2022; Tofighi et al., 2019; Ubhi, Kotz, et al., 2016). Reviews of different types of DBCIs for smoking cessation developed in a research context have found that these DBCIs are more effective than non-active controls (Amiri & Khan, 2023; Graham et al., 2016; Griffiths et al., 2018; Kingkaew, 2018; Liu et al., 2023; McCrabb et al., 2019; Taylor et al., 2017; Whittaker et al., 2019). However, their effectiveness compared to active controls is unclear (Graham et al., 2016; Griffiths et al., 2018; Liu et al., 2023; Taylor et al., 2017; Whittaker et al., 2019).

Ascertaining what differentiates more from less effective interventions can offer insight into their active ingredients, which can then aid in the designing of more effective interventions in the future. However, previous systematic reviews that have tried to identify which content and delivery features of interventions are the active ingredients have reached divergent conclusions (Bartlett et al., 2014; Black et al., 2020; de Ruijter et al., 2022; Griffiths et al., 2018; Kingkaew, 2018; McCrabb et al., 2019). The reasons for these different findings are not entirely clear. However, it may be partly attributable to different inclusion criteria and interactions between different ‘entities’ of each behaviour change intervention ‘scenario’ (Michie et al., 2020). That is, different attributes of each intervention and study are included in the review, such as intervention content, delivery mode, and the target population. Specifically, there are indications that the effectiveness of DBCIs for smoking cessation may vary according to the socioeconomic characteristics of the population using them (J. Brown et al., 2014; Coleman et al., 2022). Interventions relying on or targeting specific mechanisms may be more or less effective for people in different socioeconomic positions due to differences in material and social circumstances, resources, and barriers, including general and (digital) health literacy as well as smoking-related and quitting-related cognitions (Estrela et al., 2023; Hiscock et al., 2012; Twyman et al., 2014). This may contribute to socioeconomic disparities in quit success (Hiscock et al., 2012). However, the mechanisms underlying this potential effect moderation by socioeconomic position are still unclear (Kock et al., 2019; Siddiqi & Dogar, 2014; Smith et al., 2021). Another possibility is that there are synergistic or antagonistic effects when certain intervention contents are combined with each other or certain delivery features. For example, a certain intervention content (e.g., strengthening of the non-smoker identity) may be effective in the presence of certain other content (e.g., focus on past success) or when delivered by text messaging where it is readily accessible in the environment where a person usually smokes, compared with delivery on a website, where accessing it might be more difficult in the context where a person usually smokes. The past decade has seen substantial advances in behaviour change theory (e.g., Michie et al., 2013, 2020) and statistical techniques (e.g., Li et al., 2017). These innovations allow us to explore divergent findings and heterogeneity in study results in new ways to further our understanding of what works for whom under which circumstances.

There are a few taxonomies that can be used to reliably code different attributes of DBCIs to compare interventions, identify potential active ingredients, and guide the development of new interventions. One of the most comprehensive, reliable, and well-validated taxonomies of potential active ingredients is the Behaviour Change Technique Taxonomy v1 (BCTTv1) (Abraham et al., 2015; Michie et al., 2013; Ogden, 2016). Behaviour Change Techniques (BCTs) are generalisable content components of a behaviour change intervention that can be observed and replicated (Michie et al., 2013). The BCTTv1 has been used in several reviews aiming to identify active ingredients of smoking cessation interventions (Bartlett et al., 2014; Black et al., 2020; de Ruijter et al., 2022; Griffiths et al., 2018; Kingkaew, 2018; McCrabb et al., 2019). However, reviews of the relationship between intervention attributes besides BCTs, including delivery features (such as delivery mode, language, and usability), and intervention effectiveness are scarce (Bartlett et al., 2014; Black et al., 2020; Griffiths et al., 2018). This is partly due to a lack of coherent, widely used, well-validated taxonomies to conceptualise the intervention attributes (Lehto & Oinas-Kukkonen, 2011; Oinas-Kukkonen & Harjumaa, 2009; Sama et al., 2014; Yang, 2021). However, a taxonomy of ease-of-use features for DBCIs has been developed and found to have good to acceptable reliability (Ubhi, Kotz, et al., 2016; Ubhi, Michie, et al., 2016). Additionally, readability can be reliably assessed using the Gunning-Fog-Index (Gunning, 1969), a standardised and widely used measure of text complexity (Bothun et al., 2021; Grossman et al., 1994; Kue et al., 2021; Szmuda et al., 2020). The delivery mode can be coded based on a recently developed and validated ontology (Marques et al., 2020).

In summary, a better understanding of which features of DBCIs make them effective for whom is necessary to develop improved interventions to decrease smoking rates and improve population health and reduce health inequalities.

## Aims and research questions

The aim of this review is to identify which intervention content and delivery features of DBCIs for smoking cessation are associated with intervention effectiveness and to explore whether and how socioeconomic position (SEP) moderates this relationship.

Specifically, this systematic review seeks to answer the following questions:

1. What are the associations between the effectiveness of DBCIs for smoking cessation and (i) readability (ii) ease-of-use, (iii) behaviour change techniques, and (iv) mode of delivery?
2. To what extent are these associations moderated by SEP?

## Methods

This systematic review follows the Preferred Reporting Items for Systematic Reviews and Meta-Analysis (PRISMA) and Cochrane Collaboration guidelines (Hartmann-Boyce & Lindson, 2023; Higgins et al., 2022; Page et al., 2021). Certainty of evidence as classified using the GRADE approach (Guyatt et al., 2008).

### Search strategy and selection criteria

The search strategy was developed in collaboration with a subject librarian. MEDLINE, PsycINFO, EMBASE, CINAHL, ASSIA, Web of Science Core Collection, the ACM Digital Library, the IEEE Digital Library, the Cochrane Central Register of Controlled Trials (CENTRAL), and ProQuest Dissertations were searched from 2004 to 21 February 2023, using keywords and database-specific subject headings related to the concepts of ‘digital interventions’ (e.g., cell phone* or cellular phone* or mobile phone* or mobile device* or smart device* or phone-based or phonebased or smartphone* or app or apps or telecommunication or telehealth or telemedicine or ehealth or e health or internet or online or on line or digital* or computer* or laptop* or iPad* or (tablet adj5 (internet or computer or digital or mobile or internet or electronic or cellular or web or online or on line or smart* or mobile*)) or m health or mhealth or u health or uhealth or email or e mail or electronic mail or text messag* or SMS or chat-based or text-based or multimedia messag* or MMS or social media), ‘smoking cessation’, (e.g., (quit* or stop* or give* or ceas* or cess*) adj3 (smok* or tobacco* or cigar* or pipe* or shisha* or hookah* or nargile* or narghile* or waterpipe*)) and ‘experimental evaluation’ (e.g., compare or compared or comparison or randomised controlled trial or controlled clinical trial or placebo or drug therapy or random* or groups or trial or experiment* or quasiexperiment* or (pilot adj2 stud*) or (feasib* adj2 stud*)). Equivalent terms were connected with the Boolean operator ‘or’ while those referring to different concepts were connected using the Boolean operator ‘and’. In databases where they were available, medical subject headings were used. Additionally, the dblp computer science library, the British Library e-theses online service (eThOS), and the National Institute for Health Research UK Be Part of Research (formerly Clinical Trials Gateway) were hand-searched in May and June 2023. Reference list searches of reviews on similar topics were undertaken in May and June 2023. The full search strategy used for each database is provided in section A of the supplementary files. The search strategy was limited to studies published since 2004; this is often selected as the year in which digital technology, and specifically the internet, shifted towards primarily interoperable, user-centred applications and services to promote sharing of media and information, collaboration, and social connectedness (Roser et al., 2015; Wilson et al., 2011). DBCIs from before this period are likely to be incommensurable with later ones and of limited relevance to the development of future DBCIs.

To be included in the review, studies needed to report experimental or quasi-experimental evaluations of individual-level smoking cessation interventions delivered primarily through digital technologies (West & Michie, 2016). Our definition of digital technologies included, but was not limited to, email, websites or web-based games, mobile or tablet applications, SMS text messages, MMS multimedia messages, social media, and wearables. Interventions that consisted of a real-time talk-based intervention delivered over the phone were not included. Only studies using abstinence from smoking combustible tobacco (such as cigarettes, cigars, pipes, waterpipes, and cigarillos) as an outcome were included. This means that studies measuring only smoking reduction or measuring abstinence from electronic nicotine delivery systems, non-combustible tobacco, or nicotine replacement therapy were not included. We did not apply any geographical limits on where the study could be conducted, but the intervention needed to be conducted in the English language. This was a pragmatic choice to allow us to code readability in a reliable and valid manner. No restrictions were placed on study populations; studies conducted with adults or adolescents, in a community or clinical context, and studies looking exclusively at specific subpopulations, such as pregnant people, people with chronic illnesses, and people in low SEPs, were all eligible for inclusion. No restrictions were applied on the control condition.

Only studies reporting a measure of SEP were included to allow us to analyse effectiveness and moderators of effectiveness by SEP. If studies did not report differential effects by SEP, authors were contacted up to three times to request the relevant data and materials at either the participant or summary level.

### Measures

Intervention effectiveness, measured in terms of the odds that a participant in the intervention group would be abstinent from smoking compared with a participant in the control group, was the primary outcome. The odds ratios (ORs) and 95% confidence intervals (CIs) of abstinence outcomes were calculated using the data extracted from each study and any additional data provided by the authors. The most rigorous measure of abstinence (Piper et al., 2020; West et al., 2005) reported or made available by the study authors was extracted. This means that if a study, for example, reported both 30- and 7-day point-prevalence abstinence at 6 months, the 30-day measure was used.

Readability, ease-of-use, modes of delivery, and BCTs were used to characterise intervention content and delivery features. Study authors were contacted to request intervention materials in order to characterise these intervention features.

The readability of the intervention text was assessed using the Gunning-Fog-Index (GFI; Gunning, 1969), which is a reliable, standardised, and widely used measure of text complexity (Bothun et al., 2021; Grossman et al., 1994; Kue et al., 2021; Szmuda et al., 2020). Based on classifications of reading difficulty by the National Work Group on Literacy and Health and NHS guidelines for the readability of patient information, the scores were split into three categories (NHS Health Education England, n.d.; Report of the National Work Group on Literacy and Health, 1998): ‘Easy’ (GFI of 6 or below); ‘Average’ (GFI above 6 and below or equal to 9); ‘Difficult’ (GFI of above 9); and not text-based/written. For the moderator analysis, the categories ‘Easy’ and ‘Average’ were collapsed into one because only one intervention was categorised as ‘Easy’.

Ease-of-use features were coded based on the ease-of-use taxonomy (Ubhi, Michie, et al., 2016). This ease-of-use taxonomy has been specifically developed for DBCIs and has been previously found to have a good to acceptable reliability (Ubhi, Kotz, et al., 2016; Ubhi, Michie, et al., 2016). We modified this taxonomy to exclude the readability item as it was assessed separately with the GFI. Therefore, overall ease-of-use was scored from 0 (none of the features are present) to 8 (all the features are present).

The mode of delivery was coded based on a recently developed and validated ontology that outlines various modes of delivery, including ones using digital technologies (Marques et al., 2020).

Intervention content was coded using BCTs from the BCTTv1 (Michie et al., 2013). The BCTTv1 contains 93 BCTs across 16 BCT domains that can be mapped on the COM-B (Capability, Opportunity, Motivation, Behaviour) and Theoretical Domains Framework (TDF) models of behaviour as well as the Behaviour Change Wheel (BCW) guide for intervention development (Atkins et al., 2017).

If a study included multiple control conditions, the most minimal control was included in the quantitative synthesis, in line with recommendations by the Cochrane Collaboration (Higgins et al., 2022). To determine the most minimal control condition, we, hierarchically, either picked the inactive control condition if one existed, followed the authors’ description in the original paper, or used our own judgment and knowledge of the research literature to determine which control condition was the least active (e.g., if a study compared a more active study app to both a less active app and print materials, we chose the print materials as the most minimal control).

Study quality and risk of bias were assessed using the current version of the Cochrane Risk of Bias Tool (Sterne et al., 2019).

### Data extraction

The literature search results, deduplication, abstract screening, and full-text sifting of records were managed using Rayyan (Ouzzani et al., 2016; Rayyan – Intelligent Systematic Review – Rayyan). Abstract screening and full-text sifting were done by comparing study characteristics to tabulated inclusion and exclusion criteria. Interrater agreement was assessed using kappa coefficients as appropriate. The strength of agreement as indicated by kappa coefficients can be interpreted as follows: poor (<0.00), slight (0.00–0.20), fair (0.21–0.40), moderate (0.41–0.60), substantial (0.61–0.80), almost perfect (0.81–1.00) (Landis & Koch, 1977).

CL did the literature search, screened all abstracts and full texts, extracted the data, and assessed all studies for risk of bias. TO screened 20% of abstracts to assess the reliability of inclusion (PABAK: 0.783, 95% CI: 0.745–0.818). To assess reliability in the coding of intervention content and delivery features, TO also double-coded 20% of included studies for BCTs (PABAK: 0.778; 95% CI: 0.738–0.813), ease-of-use (PABAK: 0.432; 95% CI: 0.220–0.614), and mode of delivery (100% agreement). TO also assessed 20% of included studies for risk of bias (overall risk of bias Fleiss’ *κ* = 0.40, *p *= .003; risk of bias for individual questions Fleiss’ *κ* = 0.63, *p* < .001). All disagreements were resolved through discussion to reach a consensus.

### Analyses

All studies were included in the analyses for which authors provided sufficient data and intervention materials to estimate differential effect sizes by SEP and code the relevant intervention features. Random-effects models were used because there was likely residual heterogeneity between intervention effect sizes that was not accounted for by the moderators investigated and the ‘true’ effect sizes of the populations were likely to be different (Borenstein et al., 2010). All analyses were performed in RStudio version 4.3.1. Conventional meta-analyses were performed using the ‘metafor’ package (Viechtbauer, 2010) and meta-CART analyses were conducted using the ‘metacart’ package (Li et al., 2017, 2019).

#### Meta-analysis

A conventional random-effects meta-analysis was performed on the overall effect sizes of all included studies to estimate the overall effectiveness of DBCIs for smoking cessation (*K* = 29). Additionally, subgroup random-effects meta-analyses using the separate effect sizes for high (*K* = 27) and low (*K* = 28) SEP were conducted. Subgroup analyses stratified by whether the control condition was active (*K* = 14) or inactive (*K* = 15) were performed. The meta-analyses were also repeated on an extended dataset including studies for which authors provided sufficient data to estimate differential effect size by SEP, but not sufficient intervention materials to allow for coding of relevant intervention features (*K* = 48).

#### Meta-CART

To explore moderators of effect size, meta-CART analyses were conducted. Meta-CART is a data-driven meta-analytic method to identify moderators of effect size (Li et al., 2017, 2019). As such, it has a higher power to detect main effects and interactions between multiple moderators of effect size than traditional methods such as multivariate meta-regression and subgroup meta-analysis (Li et al., 2017, 2019). In line with the pre-registered review protocol, the following moderators were added to meta-CART model: Mode of delivery (dichotomised into mobile-based and not-mobile-based); ease-of-use (dichotomised using a median split); readability (three categories: ‘not text-based/written’, ‘easy or average’ [GFI of 9 or below], and ‘difficult’ [GFI over 9]); SEP (dichotomised as high or low based on the measure used by the authors of the original study); BCTs (numerical variable coding whether each BCT was present in the intervention, but not control condition [1], present in both control and intervention or neither [0], or present in the control, but not intervention condition [−1]); individual BCTs were included as potential moderators if they were present in the intervention, but not control condition for at least four effect sizes (63/93 BCTs, see section B of the supplementary files). Two types of sensitivity analyses were performed in which BCTs were entered as binary categorical variables: one in which all observations which had a BCT that was present in the control, but not the intervention condition were dropped, and one where all −1 cases (present in control but not intervention) were recoded to 0 (absence in intervention). The meta-CART analysis used random-effects analysis, with the pruning parameter set to *c* = 1 (Li et al., 2019). The other parameters were kept at their default values (maximum number of splits = 5; minimum number of studies in parent node before splitting = 6; stopping rule for the decrease of between subgroups *Q* = 0.00001; minimum number of studies in a terminal node = 3, 10-fold cross-validation).

#### Sensitivity analyses

Exploratory random-effects meta-regressions were used to examine the robustness of the findings from the meta-CART analysis. For these exploratory meta-regressions, moderators were first entered into their own univariate meta-regressions that controlled for false discovery rate using the Benjamini–Hochberg correction (Benjamini & Hochberg, 1995). If multiple moderators were found to be significant in these corrected univariate meta-regressions, they were added to a multivariate meta-regression.

Sensitivity analyses excluding studies at high risk of bias, using self-reported abstinence as the outcome, with high (>50% (Hartmann-Boyce & Lindson, 2023)) dropout, and representing outliers in effect size were performed for both the conventional meta-analyses and the meta-CART analysis. To explore the robustness of the meta-CART algorithm, analyses were repeated with the pruning parameter set to *c* = 0.5 and *c* = 0.0 to allow for greater power (while controlling the Type 1 error less tightly) (Li et al., 2017, 2019). In further sensitivity analyses of the meta-CART analyses, BCT domains were used instead of BCTs and mode of delivery was left as a categorical non-dichotomised variable.

### Pre-registration

The study protocol was pre-registered on OSF (https://osf.io/4cgdb/) and Prospero (ID: CRD42023398393) after conducting preliminary searches but before starting systematic literature searches.

#### Changes to the pre-registration

There was an error in the pre-registered protocol, which stated that readability would be categorised in the following way: Easy (Gunning-Fog-Index of 6 or below); Average (Gunning-Fog-Index of 9 or below); Difficult (Gunning-Fog-Index of 10 or above). This left a gap between the categorisations for average and difficult, and categorisation was corrected so that the category of difficult started from a Gunning-Fog-Index of above 9. A planned sensitivity analysis excluding studies that were quasi-randomised was not performed since there were no quasi-randomised studies. BCTs were added as numerical, rather than categorical, variables to also account for occasions where the BCTs were present in the control, but not in the intervention condition. However, since entering BCTs as quasi-continuous variables has the – likely not warranted – assumption that the presence of a BCT in the intervention and control is equivalent. It also does not test specifically whether using a certain BCT as part of an intervention is linked to the effectiveness of that intervention. Therefore, two types of sensitivity analyses in which BCTs were entered as binary variables were added as well: one in which all observations which had a BCT that was present in the control, but not the intervention condition were dropped, and one where all −1 cases were recoded to 0 s.

The following unplanned sensitivity analyses were added: Because the pre-planned meta-CART analysis did not find evidence of effect moderation, exploratory meta-regressions were used to examine the robustness of these findings. Conventional meta-analyses were also repeated on an extended dataset including studies for which authors provided sufficient data to estimate differential effect size by SEP, but not sufficient intervention materials to allow for coding of relevant intervention features.

## Results

### Search and sift

**Figure 1.**
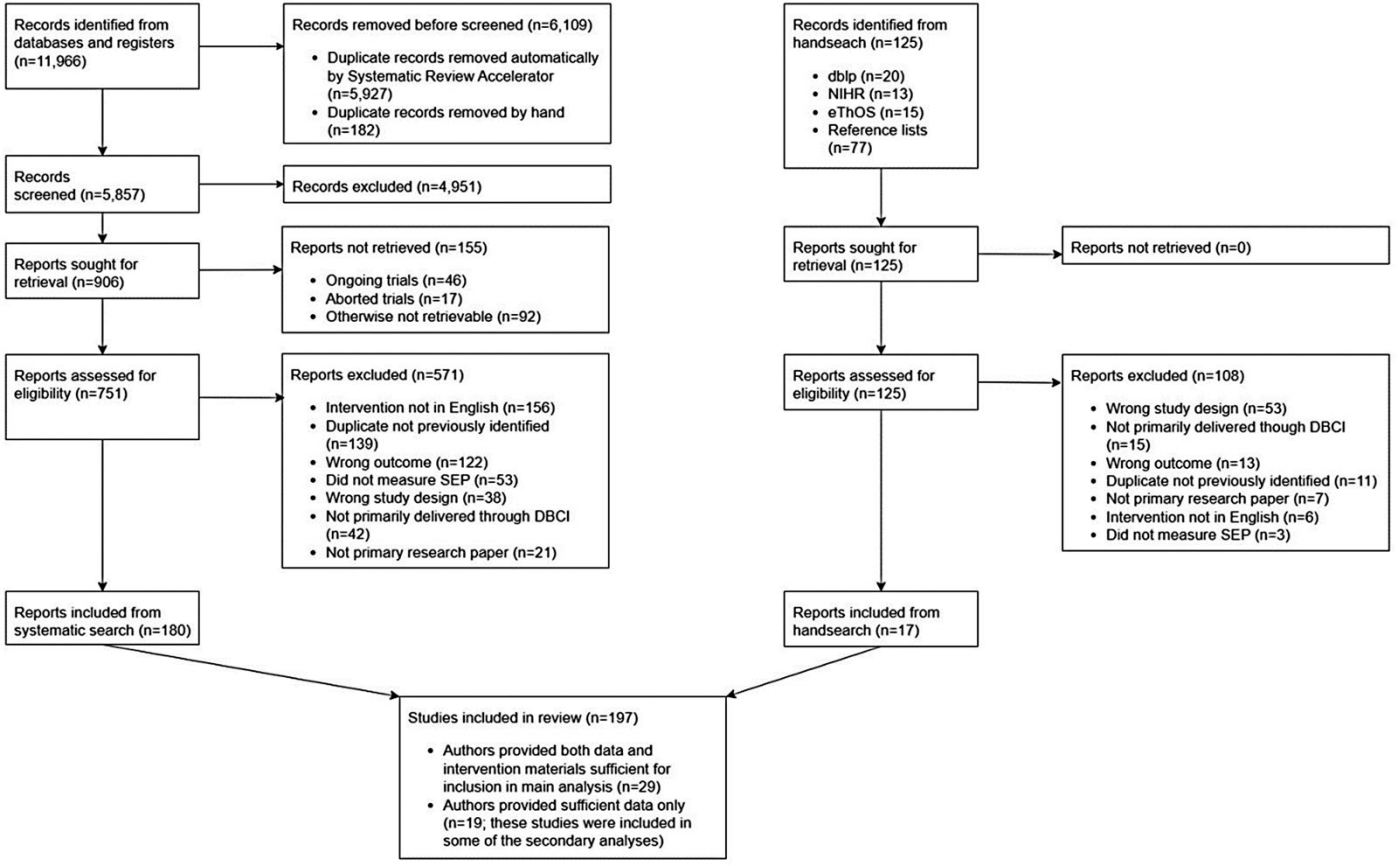
Study selection.

A total of 11,966 studies (5857 after exclusion of duplicates: 5927 duplicates automatically removed using Systematic Review Accelerator (Clark et al., 2020); 182 additional duplicates removed manually) were identified by the systematic searches of the above-mentioned databases, 751 full-text reports were retrieved and screened for eligibility (Figure 1). Of these, 180 met the inclusion criteria. Additionally, 125 records were identified through hand-search, retrieved, and screened for eligibility. Of these, 17 met the inclusion criteria. This yielded an overall sample of 197 studies. For 29 of these studies, authors provided sufficient data and intervention materials to estimate differential effect size by SEP and allow for the coding of relevant intervention features. For an additional 19 studies, authors provided sufficient data to estimate differential effect size by SEP, but not sufficient intervention materials to allow for coding of relevant intervention features. A list of all studies eligible for inclusion can be found in section C of the supplementary files.

### Descriptives

Details on study and intervention characteristics can be found in Table 1 and Table 2, respectively. Details on the extended set of studies used in some of the sensitivity analyses can be found in section D of the supplementary files.

**Table 1.** Study characteristics of the 29 studies included in the main analysis.

Study	Study type	Country	*N* Overall (*N* intervention (I) | *N* control (C))	Inclusion criteria	Age: mean (SD) I|C	% Female I|C	SEP measure	% low SEP I|C	Baseline CPD: mean (SD) I|C	Baseline nicotine dependence measure: mean (SD) I|C	Abstinence measure	Follow-up time	% Followed up I|C
Baskerville (2018)	RCT	Canada	851 (426 | 425)	Ages 19–29, motivated to quit, smoking daily	Only categorical information	45.4 | 48.6	Education	39.6 | 42.6	Only categorical information	HSI: only categorical information	Self-reported sustained abstinence (3 months)	24 weeks (6 months)	43.2 | 47.6
Begh (2015)	RCT	UK	118 (60 | 58)	Ages 18+, signed up to smoking cessation intervention, smoking at least 10 CPD, eCO smoking at least 10 ppm	46.5 (12.7) | 43.0 (12.7)	56.7 | 59.3	Education	51.7 | 63.8	21.8 (9.9) | 19.8 (8.5)	FTND: 5.3 (2.4) | 5.7 (2.1)	Biochemically verified sustained abstinence (Russell Standard)	24 weeks (6 months)	70.0 | 60.3
Brown (2014)	RCT	UK	4613 (2321 | 2292)	Ages 18+, motivated to quit, smoking daily	39.5 (13.0) | 38.8 (12.5)	63.0 | 62.3	Occupational social grade	46.9 | 46.0	18.7 (8.9) | 18.5 (9.0)	FTND [HSI]: 5.1 (2.4) | 5.0 (2.4) [2.9 (1.1) | 2.9 (1.0)]	Biochemically verified sustained abstinence (Russell Standard)	24 weeks (6 months)	70.8 | 72.9
Coleman (2022)	RCT	UK	1002 (501 | 501)	Ages 16+, pregnant, willing to receive smoking cessation advice, smoking at least 5 CPD pre-pregnancy; smoking at least 1 CPD during pregnancy	27.1 (5.6) | 27.5 (5.7)	100 | 100	Education	68.7 | 68.1	8.6 (5.5) | 8.9 (5.5)	HSI: 1.9 (1.4) | 2.0 (1.4)	Biochemically verified sustained abstinence (Russell Standard) AND 7-day PPA	36 weeks of gestation (on average 21 weeks)	61.7 | 67.3
Crane (2019)	RCT	Global (intervention in English)	28,112 (14,228 | 13,884)	Ages 18+, signed up to smoking cessation intervention, current smoking	28.7 (9.0) | 29.1 (9.4)	49.3 | 48.8	Education	62.9 | 62.9	14.6 (7.4) | 14.8 (7.6)	HSI: 2.3 (1.5) | 2.4 (1.5)	Self-reported sustained abstinence (Russell Standard)	12 weeks (3 months)	8.5 | 6.5
Dallery (2017)	RCT	USA	94 (48 | 46)	Ages 18+, motivated to quit, smoking at least 10 CPD, last cigarette within last 24 hours; smoking daily for 2 years or more	36.7 (11.2) | 34.9 (11.1)	56 | 54	Income	34 | 26	17.8(7) | 18.3(7)	FTND: 4.8 (2.0) | 4.8 (2.3)	Biochemically verified 7-day PPA	24 weeks (6 months)	79.2 | 89.1
Dingle (2017)	RCT	Australia	37 (19 | 18)	Ages 18+, signed up to smoking cessation intervention, current smoking	40.4 (9.6) | 41.4 (12.4)	36.8 | 33.3	Education	47.4 | 55.6	Not reported	FTND: 4.9 (2.5) | 4.7 (2.5)	Self-reported 7-day PPA	6 weeks	57.9 | 72.2
Garrison (2020)	RCT	USA	505 (245 | 260)	Ages 18+, motivated to quit, smoking at least 5 CPD, abstinent for less than 3 months in past 12 months	42.9 (11.6) | 40.6 (12.2)	69.0 | 71.2	Education	20.8 | 17.7	17.00 (8.44) | 16.62 (7.89)	None	Biochemically verified 7-day PPA	24 weeks (6 months)	78.4 | 73.9
Haaga (2020)	RCT	USA	278 (146 | 132)	Ages 18+, no motivational requirement, smoking at least 10 CPD, smoking daily, eCO at least 9 ppm	Only overall 49.8 (11.4)	Only overall 47.8	Income	63.0 | 75.0	Only overall 16.62 (8.13)	FTND: only overall 5.4 (2.0)	Biochemically verified 7-day PPA	4 weeks (1 month)	87.7 | 89.4
Herbec (2014)	RCT	UK	200 (99 | 101)	Ages 18+, pregnant, motivated to quit, smoking daily	27.6 (6.0) | 28.1 (5.8)	100 | 100	Occupational social grade	54.6 | 41.6	14.6 (5.2) | 14.7 (7.8)	FTND [HSI]: 4.6 (1.7) | 4.3 (1.6) [2.7 (1.4) | 2.7 (1.4)]	Self-reported 30-day PPA	8 weeks (2 months)	63.6 | 69.3
Herbec (2019)	RCT	UK	425 (208 | 217)	Ages 18+, motivated to quit, smoking daily	33.1 (10.1) | 32.8 (11.4)	44.7 | 46.1	Education	29.8 | 32.7	15.08 (7.32) | 15.58 (7.03)	HSI: 2.6 (1.4) | 2.5 (1.4)	Self-reported sustained abstinence (Russell Standard) AND 7-day PPA	26 weeks (6.5 months)	38.0 | 42.4
Jackson (2023)	RCT	Global (intervention in English)	3143 (1564 | 1579)	Ages 18+, motivated to quit, current smoking	49.2 (11.6) | 48.9 (11.4)	74.0 | 75.5	Education	34.5 | 37.1	18.0 (8.7) | 18.2 (10.0)	Time to first cigarette: only categorical information	Self-reported sustained abstinence (Russell Standard)	28 weeks (7 months)	33.8 | 36.4
Kahler (2020)	RCT	USA	119 (58 | 61)	Ages 18+, drinking at heavy or at-risk levels, signed up to smoking cessation intervention, smoking daily	37.1 (10.6) | 38.6 (12.9)	71 | 69	Education	25.9 | 19.7	18.4 (10.5) | 15.9 (7.4)	FTND: 5.3 (2.3) | 4.9 (2.3)	Self-reported 7-day PPA	24 weeks (6 months)	56.9 | 49.2
King (2022)	RCT	UK	28 (15 | 13)	Ages 16+, pregnant, no motivational requirement, current smoking, eCO at least 4 ppm	27.2 (6.1) | 24.4 (5.6)	100 | 100	Education	33 | 67	Only categorical information	Time to first cigarette: only categorical information	Self-reported sustained abstinence (6 weeks; abstinence since last appointment)	12–13 weeks post due date (approx. 40 weeks (10 months) post randomisation)	40 | 23
McRobbie (2020)	RCT	UK and Australia	234 (116 | 118)	Ages 18+, ex-smoker 7–100 days post quit, abstinent smokers who quit 7–100 days ago	Median between 43 and 44 | Median between 44 and 46	47.4 | 48.3	Income	58.2 | 52.2	0 | 0	HSI: only categorical information	Self-reported sustained abstinence (no more than 7 consecutive days of smoking in past 6 months and not even a puff in last month)	24 weeks (6 months)	87.1 | 89.8
Mutter (2020)	RCT	USA	346 (172 | 174)	Ages 18+, interested in smoking reduction or cessation, smoking at least some days in past 14 days	34.2 (10.5) | 34.5 (10.5)	47.7 | 42.5	Education	12.8 | 17.8	12.06 (9.01) | 10.92 (8.32)	Five-item version of the Cigarette Dependence Scale (CDS-5): 17.0 (4.1) | 16.4 (4.7)	Self-reported 7-day PPA	4 weeks (1 month)	75.6 | 77.0
Naughton (2014)	RCT	UK	602 (299 | 303)	Ages 18–75, motivated to quit, smoking at least one CPD, smoked in last 7-days	42.3 | 41.3	53.2 | 52.1	Occupational social grade	46.8 | 40.9	18.4 (7.9) | 18.2 (8.2)	None	Biochemically verified sustained abstinence	24 weeks (6 months)	76.6 | 78.6
Naughton (2017)	RCT	UK	407 (203 | 204)	Ages 16+, pregnant, willing to receive smoking cessation advice, smoking at least 5 CPD pre-pregnancy, smoking at least one CPD during pregnancy	26.6 (5.7) | 26.4 (5.7)	100 | 100	Education	75.9 | 73.5	9.0 (5.9) | 9.4 (6.1)	HSI: only categorical information	Biochemically verified sustained abstinence (Russell Standard)	36 weeks of gestation (on average 21–22 weeks)	63.6 | 64.7
Naughton (2023)	RCT	UK	209 (104 | 105)	Ages 16+, motivated to quit, smoking at least 7 cigarettes per week	39.6 (10.0) | 42.6 (10.0)	56.7 | 54.3	Occupational social grade	23.1 | 11.4	15.4 (7.6) | 15.5 (6.5)	HSI: only categorical information	Biochemically verified sustained abstinence (Russell Standard)	24 weeks (6 months)	78 | 75
Palmer (2023)	RCT	USA	34 (19 | 15)	Ages 18+, motivated to quit, smoking least 5 CPD smoking at least 25 days in past month; smoking for over 1 year	43 (12) | 45 (13)	36.8 | 40.0	Education	31.6 | 33.3	17.05 (5.89) | 19.44 (9.91)	Time to first cigarette: only categorical information	Self-reported 3 day PPA	4 weeks (1 month)	100 | 100
Pbert (2020)	Matched cluster RCT	USA	96 (48 | 48)	High school students, motivated to quit, smoking at least 5 CPD	16.9 (1.1) | 16.7 (1.1)	60.4 | 54.2	Income	68.8 | 68.8	4.79 (3.11) | 4.93 (4.21)	None	Biochemically verified 7-day PPA	24 weeks (6 months)	92 | 98
Spears (2019)	RCT	USA	36 (3 | 33)	Ages 18+, motivated to quit, abstinent smokers who quit 7–100 days ago	45.6 (12.4) | 45.6 (12.0)	45 | 61	Income	48 | 33	14.4 (9.4) | 18.8 (9.3)	Time to first cigarette: only categorical information	Biochemically verified 7-day PPA	7 weeks	86.8 | 90.9
Sridharan (2019)	RCT	USA	398 (199 | 199)	Ages 18+, motivated to quit, smoking at least 5 CPD, smoking for smoking at least 1 year	42.1 (12.0) | 42.0 (12.5)	59 | 58	Income	31.7 | 29.6	19.1 (15.9) | 19.0 (16.5)	FTND: 5.8 (2.0) | 5.9 (2.1)	Biochemically verified 30-day PPA	8 weeks (2 months)	90.5 | 93.0
Stoops (2009)	RCT	USA	68 (35 | 33)	Ages 18+, no motivational requirement, smoking at least 10 CPD, eCO at least 8 ppm	38 |40	74.3 | 75.8	Education	20.0 | 42.4	30 | 30	FTND: 5 | 5	Biochemically verified 7-day PPA	5 weeks	82.9 | 87.9
Tucker (2021)	Cluster-crossover RCT	USA	81 (43 | 38)	Ages 18–25, homeless, motivated to quit, smoking at least 5 CPD on at least 20 days in past month	22.2 (1.9) | 23.0 (1.7)	22.5 | 13.9	Homelessness	100 | 100	8.17 (7.07) | 9.32 (5.71)	None	Self-reported sustained abstinence	12 weeks (3 months)	83.7 | 79.0
Vilardaga (2020)	RCT	USA	62 (33 | 29)	Ages 18+, diagnosis of serious mental illness, motivated to quit, smoking at least 5 CPD, eCO at least 6 ppm	46.1 (11.3) | 45.6 (10.9)	64 | 55	Education	33.3 | 28.6	21 (15.5) | 14 (6.4)	FTND: 5.2 (2.6) | 4.7 (2.3)	Biochemically verified 7-day PPA	16 weeks (4 months)	100 | 96.6
Villanti (2022)	RCT	USA	437 (229 | 208)	Ages 18–30, socially disadvantaged, motivated to quit, current smoking; smoking at least 100 cigarettes in lifetime	only overall 25.6 (3.3)	86.6 | 82.5	Subjective financial status	100 | 100	12.2 (10.7) | 12.7 (10.3)	FTND: 1.9 (1.3) | 2.1 (1.2)	Biochemically verified (cut-offs not reported) 30-day PPA	12 weeks (3 months)	48.5 | 68.3
Walker (2020)	RCT	UK	73 (50 | 23)	Ages 18–55, female, no motivational requirement, smoking at least one cigarette per week	25.5 (7.8) | 25.3 (7.5)	100 | 100	Education	2 | 0	Only categorical information	None	Self-reported 7-day PPA	12 weeks (3 months)	76.0 | 69.6
White (2019)	RCT	USA	54 (27 | 27)	Ages 18+, overweight or obesity, no motivational requirement, smoking at least 10 CPD, less than three consecutive months of abstinence in past year	46.4 (11.6) | 45.4 (9.6)	74.1 | 70.4	Education	66.7 | 70.4	18.9 (5.7) | 20.5 (10.7)	None	Biochemically verified (substantially more lenient cut-off than standard) sustained abstinence	24 weeks (6 months)	96.3 | 66.7

Notes: CPD = Cigarettes per day, eCO = expired carbon monoxide, FTND = Fagerström Test for Nicotine Dependence, HSI = Heaviness of smoking index, PPM = Parts per million, RCT = Randomised controlled trial, SD = Standard deviation, SEP = Socioeconomic position, UK = United Kingdom of Great Britain and Northern Ireland, USA = United States of America.

**Table 2.** Intervention characteristics of the 29 studies included in the main analysis.

Study	Description of digital intervention	Description of digital control	Non-Digital Intervention Aspect (Intervention Condition Only)	Non-Digital Intervention Aspect (Both Groups)	Ease-of-Use Score	Mode of Delivery	Gunning-Fog-Index
Baskerville (2018)	Crush the Crave: App and text messaging intervention enabled users to personalise their quit plan, set a quit date, and gradually reduce smoking before it or quit abruptly based on their preferences. The app sent reminders of saved money and health benefits and offered rewards. Participants also received delivered tailored text support. They could also track smoking habits, cravings, and identify triggers, with additional online distractions for managing cravings	On the Road to Quitting Self-Help: Self-help guide could be accessed online or as a print copy. It included information on health and financial benefits, triggers, withdrawal strategies, quitting methods, support networks, counselling, and dealing with relapse	None	None	6	Messaging (Mobile digital device); Mobile application	6.5
Begh (2015)	Smoking specific attentional bias retraining through visual probe task	Placebo/sham attentional bias retraining through visual probe task	None	Seven sessions of behavioural support at UK stop smoking services; 8–12-week supply of transdermal nicotine	7	Computer	NA
Brown (2014)	StopAdvisor: Interactive website developed based on PRIME theory, behaviour change techniques, web-design principles, and user testing. Simulated an expert stop smoking advisor. Offered tailored support with interactive menus and tunnelled sessions, focusing on goal setting, action planning, and guidance on use of pharmacotherapy. Support extended up to one month post quit date	Static website: Provided brief, standard advice on setting a quit date, mirroring the interactive site. Encouraged medication use, obtaining it within two weeks, and setting a quit date within the same timeframe, ensuring comparable follow-up times since quit date for smokers in both conditions	None	None	8	Website; Email	8.9
Coleman (2022)	MiQuit: Automated responsive text messaging programme that provided 12-week support for smoking cessation. Tailored messages, utilising 14 recipient characteristics, encouraged quit attempts, and helped set a quit date. Personalised content covered foetal development, motivation, cravings, triggers, and withdrawal. Users could adjust message frequency and access on-demand support	No digital aspect	None	Usual care	8	Messaging (Mobile digital device)	7.1
Crane (2019)	Smoke Free app with daily missions: App offered for free during the study encouraged users to set a quit date and provided advice and motivational support for smoking cessation. Grounded in behaviour change techniques, it provided tools like savings calculator, progress tracking, virtual badges, health indicators, cravings diary, daily missions, chatbot, and 24/7 access to a stop smoking advisor. Users were given daily missions to complete for a month starting from their quit date. These missions were designed to help them resist cravings and improve their chances of staying smoke-free. Users received daily push notifications at 8am local time to remind them to read the day’s mission, but they could customise the notification time if they preferred	Smoke Free app without daily missions: App offered for free during the study encouraged users to set a quit date and provided advice and motivational support for smoking cessation. Grounded in behaviour change techniques, it provided tools like savings calculator, progress tracking, virtual badges, health indicators, cravings diary, daily missions, chatbot, and 24/7 access to a stop smoking advisor. The reduced version of the app was the same as the full version but without the daily missions	None	None	5	Mobile application	8.6
Dallery (2017)	Remote contingency management: Participants could earn up to $480 in rewards for abstinence, verified by video-recorded eCO samples submitted to the study website	Non-contingent control: Participants could earn up to $480 as reward for submission of eCO samples submitted to the study website	None	‘Clear the Air’ packet from Smokefree.gov	5	Website; Electronic environmental object	7.7
Dingle (2017)	MUSIC Smoke into Sound: Music-listening-based emotional regulation programme that aimed to teach participants to substitute smoking with music listening for emotional regulation. Using a two-dimensional emotion model, participants were instructed to select music to achieve desired emotional states, creating a ‘quit’ playlist for overcoming cravings and triggers	No digital aspect	None	Treatment as usual from local telephone quitline	7	Website	NA
Garrison (2020)	Craving to Quit: App providing a 3-week mindfulness-based smoking cessation programme. It included 22 10–15-minute modules with psychoeducational audio/videos, animations, and in vivo exercises. Users monitored habits, identified triggers, and practiced mindfulness, aiming to quit within 3 weeks. Five bonus modules were unlocked post-completion for extra reinforcement	Experience sampling app: 3-week comparator intervention using experience sampling only. Users received six daily prompts for 22 days to help them monitor their smoking habits, moods, and experiences, aiming to quit within 3 weeks	None	Recommendation of pharmacotherapy	7	Mobile application	NA
Haaga (2020)	Looming vulnerability intervention: Four brief audiotape-guided imagery exercises, approximately 3 minutes each. Participants envisioned scenarios wherein smoking accelerates adverse health effects, emphasising the potential mitigation through reduced smoking and cessation as the sole means to halt such consequences	Emotionally neutral comparator intervention: four 3-minute audiotape-guided imagery exercises, devoid of smoking references or consequences. These scenarios, matched in duration to looming vulnerability scenarios, integrated elements of spatial or temporal movement, maintaining emotional neutrality	None	Smoking cessation handout and information about freely accessible web- and phone-based quit support	8	Playable electronic storage	NA
Herbec (2014)	MumsQuit: Interactive website tailored for pregnant women, adapting StopAdvisor’s design and content. Offered personalised quit plans mirroring NHS Stop Smoking Services support. Provided 33 evidence-based techniques, 4 weeks of pre-quit and post-quit support. Specific pregnancy adaptations included medication adjustments, qualified NRT advice, foetal risks, quitting benefits, and imagery changes	Static website: One-page, non-personalised interface, providing standardised smoking cessation advice based on a prevalent manual for practitioners	None	None	8	Website; Email	8.9
Herbec (2019)	Enhanced Bupaquit: App integrated craving management tools, cessation support, and gamification. Users set a quit date, received pre-quit advice, post-quit support for up to 6 weeks, including medication guidance, daily notifications, craving tracking, and optional social sharing. Unlike basic version, enhanced app additionally offered craving management techniques, gamified features, and more motivational resources	Basic Bupaquit: App provided cessation support. Users set a quit date, received pre-quit advice, post-quit support for up to 6 weeks, including medication guidance, daily notifications, craving tracking, and optional social sharing	None	None	5	Mobile application	6.4
Jackson (2023)	Full version of Smoke Free: App offered for free during the study encouraged users to set a quit date and provided advice and motivational support for smoking cessation. Grounded in behaviour change techniques, it provided tools like savings calculator, progress tracking, virtual badges, health indicators, cravings diary, daily missions, chatbot, and 24/7 access to a stop smoking advisor. Participants in the intervention group also received the same static advice as the comparator group	Static advice: Website displayed a concise message urging participants to attempt quitting within same time frame as intervention, emphasising the importance of responding to follow-up requests for progress tracking. The identical message was also emailed to them promptly	None	None	5	Mobile application mode of delivery	8.6
Kahler (2020)	BecomeAnEx (enhanced): Website integrated standard version with supplementary modules addressing alcohol consumption and co-consumption of alcohol and cigarettes. Multi-component-programme was designed as a comprehensive toolbox, including features such as setting quit dates, tracking cigarettes, trigger and social support management, pharmacotherapy planning, social networking, and educational content. Additional modules offered information on the risk of smoking relapse linked to heavy alcohol use, personalised normative feedback on drinking behaviour, insights into the risks of heavy drinking and simultaneous smoking, interactive exercises evaluating the importance of modifying drinking during smoking cessation, articulating personal reasons for change, recognising the benefits of reducing alcohol intake, and setting goals to minimise smoking risk while drinking, along with selecting strategies for limiting alcohol consumption	BecomeAnEx (standard): Multi-component-programme was designed as a comprehensive toolbox, including features such as setting quit dates, tracking cigarettes, trigger and social support management, pharmacotherapy planning, social networking, and educational content. It did not address alcohol consumption or co-consumption of alcohol and cigarettes	None	None	6	Website; Messaging (Mobile digital device)	9.7
King (2022)	SKIP-IT: Automated narrative text messaging programme. Aimed to modify participants’ perceptions of risk, social norms, outcomes, and self-efficacy through three core elements: a fictional story featuring a pregnant woman overcoming smoking barriers, images illustrating foetal development, and a ‘help’ function for supportive cessation messages	No digital aspect	None	Usual care: routine eCO monitoring and referral to NHS smoking cessation services	6	Messaging (Mobile digital device)	5.4
McRobbie (2020)	Structured Planning and Prompting Protocol and text messaging: Participants were provided with a text messaging programme that included warning messages on relapse and encouragement to maintain the quit attempt. They also received an online, personalised plan that focuses on planning strategies to deal with temptations to smoke and encouraged to rehearse these strategies via text messaging	Text messaging only: Participants were provided with a text messaging programme that included warning messages on relapse and encouragement to maintain the quit attempt	None	Provision of NRT or electronic cigarette of choice (part of the sample; this is combined effect size)	7	Website; Email; Messaging (Mobile digital device)	10.8
Mutter (2020)	Mental contrasting with implementation intentions: Thought-based exercise to create firm goal commitments. It comprised two elements: identifying challenges and envisioning successful outcomes, and forming if-then plans to address internal obstacles during goal pursuit	Motivational questioning: Thought-based exercise to reflect on motivations for smoking cessation using adapted versions of five questions derived from Smokefree.gov. Participants were prompted to reflect on their motivations for quitting, thereby fostering preparation for smoking cessation	None	None	6	Website; Email	7.7
Naughton (2014)	iQuit: Personalised computer-tailored four-page advice report based on 25 questionnaire items and a 90-day text message program. Tailored content drew from smoking cessation theories, prior research, and qualitative work. Texts aimed to support quit attempts, provide information, encouragement, self-efficacy, and coping strategies, with participants able to request immediate support by texting HELP or SLIP. The number of messages sent each day varied between zero and two according to the pre-determined schedule, with participants receiving 108 messages overall	No digital aspect	None	Usual care: Routine smoking cessation advice (assessing smoking history, measuring eCO, providing brief cessation advice, setting a quit date, discussing pharmacotherapy options, prescribing, and scheduling follow-up visits)	6	Website; Messaging (Mobile digital device)	10.2
Naughton (2017)	MiQuit: Automated responsive text messaging programme that provided 12-week support for smoking cessation. Tailored messages, utilising 14 recipient characteristics, encouraged quit attempts and helped set quit dates. Personalised content covered foetal development, motivation, cravings, triggers, and withdrawal. Users could adjust message frequency and access on-demand support	No digital aspect	None	Usual care; standard self-help materials; information about additional support	8	Messaging (Mobile digital device)	7.1
Naughton (2023)	Quit Sense: Context-aware app, informed by learning theory and social cognitive theory. Targeted smoking triggers, associations, and social factors. Utilised 21 behaviour change techniques, along with main facet of geofence-triggered support. Featured training, 28-day challenge, and maintenance stages, along with feedback, surveys, profile tracking, quitting advice, and daily support messages.	Usual digital care: Text-message-delivered web link (NHS SmokeFree), deemed analogous to standard online care. NHS SmokeFree facilitates access to digital, telephonic, and in-person cessation support in England	None	None	6	Messaging (Mobile digital device); Mobile application	8.9
Palmer (2023)	Retrieval Extinction Training: 2-day smoking cessation intervention. Participants viewed two 82-minute prerecorded videos, led by a licensed psychologist. The videos featured a smoking cue, initiating memory destabilisation. Subsequently, participants underwent four 15-minute sequences of video, picture, and in vivo smoking cues. The intervention aimed to associate smoking cues with non-rewarding outcomes. The use of NRT was reviewed at the session’s conclusion	Signposting to usual care: Participants were provided contact details for phone, text, email, and web service of the local telephone quitline and informed how to access NRT	14-day supply of transdermal nicotine	None	7	Playable electronic storage; Video call	NA
Pbert (2020)	Craving to Quit: App providing a 3-week mindfulness-based smoking cessation programme. It included 22 10–15-minute modules with psychoeducational audio/videos, animations, and in vivo exercises. Users monitored habits, identified triggers, and practiced mindfulness, aiming to quit within 3 weeks. Five bonus modules were unlocked post-completion for extra reinforcement	Journeyworks pamphlets: Weekly written print materials provided by the school nurse. Pamphlets were selected for clear, attractive, low-literacy design and addressed topics like quitting smoking, social smoking, common challenges, and solutions for teens	None	Weekly school nurse visits to review progress	7	Mobile application	NA
Spears (2019)	iQuit Mindfully: Text messaging programme involved daily pre-scheduled messages (between two and six per day for 8 weeks) providing mindfulness and cognitive-behavioural strategies for smoking cessation. Participants could request additional support by texting CRAVE, STRESS, or SLIP. Post-treatment, they received between one and three texts a week, with the option to use keywords for specific assistance during a 1-month follow-up	No digital aspect	None	Weekly 2-hour sessions of mindfulness-based addiction treatment; standard self-help materials; 6 weeks of transdermal NRT	8	Messaging (Mobile digital device)	7.7
Sridharan (2019)	SmartQuit app plus a growth mindset intervention: An acceptance and commitment therapy based smoking cessation app and growth mindset advice delivered by email. Participants received daily growth mindset tip throughout the study, accompanied by email exercises every three days over 24 days. Tips and lessons were designed to be short and user-friendly, and contain less than 500 words each. Lessons covered withdrawal, genes, brain changes, personality, urges, cravings, failure to quit, and summary. Participants could download PDF files of the lessons upon completion	SmartQuit app only: An acceptance and commitment therapy based smoking cessation app	None	None	5	Website; Email	8.3
Stoops (2009)	Remote contingency management: Participants could earn up to $763 in vouchers (escalating reinforcement period) for abstinence/smoking reduction (within first week where reinforcements could be earned by reduction in eCO by set threshold) verified by video-recorded twice daily (at least 8 hours apart)	Yoked control: Exact same procedure as abstinence-contingent group, except vouchers were not contingent on own abstinence/smoking reduction but by performance of yoked ‘partner’	None	Self-help materials; information about additional support	5	Website; Electronic environmental object	7.4
Tucker (2021)	CRUSH IT!: 6-week text messaging intervention tailored to young adults experiencing homelessness who wish to quit smoking. Comprised 174 messages focusing on key areas: seeking support, financial savings, health and social benefits, managing cravings and moods, and staying motivated. Messages were designed to address specific issues faced by young adults experiencing homelessness, such as resource limitations and peer smoking, and incorporated behavioural economics strategies for enhanced impact	No digital aspect	None	30-minute group smoking cessation counselling session; supply of NRT	8	Messaging (Mobile digital device)	6.7
Vilardaga (2020)	Learn to Quit: Acceptance and Commitment Therapy based app developed specifically to help individuals dealing with severe mental health conditions quit smoking and manage mental health symptoms	NCI’s QuitGuide: App developed to help the general population quit smoking, following US Public Health Service Clinical Practice Guideline. Comprised four sections, aiming to provide health information and motivate cessation, develop a quit plan, manage cravings and replace smoking habits, cope with lapses and maintain smoke-free status	None	8-week supply of transdermal nicotine; instruction on how to use intervention	7	Mobile application	6.1
Villanti (2022)	BecomeAnEx: 12-week comprehensive, multimodal web- and text-based smoking cessation programme based on social cognitive theory and tailored for socially disadvantaged young adults. Programme included education on nicotine addiction, tools for setting quit dates, monitoring cigarette usage, recognising triggers, information on smoking cessation medication, aids in identifying social support. It also included an online community to facilitate connections and peer-to-peer advice for smoking cessation	No digital aspect	None	Referral to a telephone quitline	6	Website; Messaging (Mobile digital device)	9.8
Walker (2020)	Age progression facial morphing: Software aged a photograph of the participant to 72 years old in two different ways, one if the participant continues smoking and one if the participant stops smoking, with side-by-side presentation. Participants interacted with these images to compare aging effects. Half received reassuring instructions, while half had minimal guidance	Computerised ‘spot the difference’ game that uses comparison between pictures presented on two sides of the screen similar to intervention as an attention-matched comparator	None	Standard self-help materials	7	Computer	NA
White (2019)	QUIT + CBT: Standardised evidence-based smoking cessation programme delivered through a website. It included 12 weekly lessons addressing different aspects of smoking cessation aspects and instructed participants to choose a quit date during the second week, aligning with patch use. Additional content based on cognitive-behavioural therapy addressed weight concerns without dietary restrictions, emphasising cognitive restructuring to reduce dysfunctional thoughts about weight and shape. Weekly clinician contact via email supported CBT assignments completion on the study website	QUIT + HE: Standardised evidence-based smoking cessation programme delivered through a website. It included 12 weekly lessons addressing different aspects of smoking cessation aspects and instructed participants to choose a quit date during the second week, aligning with patch use. Additionally, to attention-match conditions, participants received general health improvement information, with online delivery and weekly clinician email support. Topics covered encompassed stress management, sleep hygiene, nutrition, physical activity, economic stress, alcohol use, and medication/drug usage	None	10-week supply of transdermal nicotine	4	Website; Email	8.7

Notes: Abbreviations: CBT = cognitive-behavioural therapy; eCO = expired carbon monoxide; HE = Health education; NCI = National Cancer Institute; NHS = National Health Service; NRT = nicotine replacement therapy; UK = United Kingdom of Great Britain and Northern Ireland; US = United States of America.

#### Study characteristics

A total of 29 studies providing *K* = 55 effect sizes split by SEP (27 for high SEP and 28 for low SEP) were included in the analyses. The studies were published between 2009 and 2023, with the majority (*k* = 19) being published in the last five years (since 2019).

All included studies were randomised trials. Sample sizes of included studies ranged from 28 to 28,112, with a mean of 1471 (SD = 5217) and a median of 209 participants. Across studies, 63.4% (SD = 21.6) of participants were female, with a minimum of 18.5% and a maximum of 100% female participants. The grand mean age of participants across the 27 studies that reported mean age was 36.2 (SD = 9.0). Across the 25 studies that reported mean cigarettes per day at baseline, the grand mean was 15.3 (SD = 4.9). Of the 29 included studies, 17 (58.6%) required participants to have some level of interest in quitting smoking as an inclusion criterion, while 2 (6.9%) required participants to be willing to receive cessation advice, and 5 (17.2%) recruited participants from a pool of users of an existing smoking cessation intervention; the remaining 5 studies (17.2%) did not have a motivation criterion. Follow-up durations raged from 4 to 40 weeks, with a mean of 17.6 (SD = 9.3) and a median of 21 weeks. Overall, 14 studies (48.3%) had a follow-up duration of at least 26 weeks (6 months). The outcome measure was self-reported abstinence for 13 studies (44.8%), while 14 studies (48.3%) used biochemically verified abstinence in line with the Russell Standard (West et al., 2005). Additionally, one study (3.5%) used biochemically verified abstinence but did not report the cut-offs used, and one (3.5%) used biochemically verified abstinence, but with substantially more lenient cut-offs than recommended in the Russell Standard. Point-prevalence abstinence was used in 15 studies (51.7%), while sustained abstinence 14 studies (48.3%). The control condition was active in 14 studies (48.3%) and inactive in the remaining 15 studies (51.7%). Active controls included reduced versions of the intervention, alternative interventions, and interventions offered through non-digital means.

#### Socioeconomic position

Measures of SEP were varied. The majority of studies (17; 58.6%) used education as an indicator. Income (personal or household) was used as an indicator of SEP in six studies (20.7%), while occupational social grade was used in four studies (13.8%). Homelessness and subjective financial status were used in one study (3.5%) each. Exact cut-offs for different categorisations also differed between studies, in part reflecting the different times and places in which the studies were conducted. The percentage of participants classified as low SEP in each study ranged from 1.4% to 100%, with a mean of 46.1% (SD = 23.8) and a median of 43.9%.

#### Study quality

**Figure 2.**
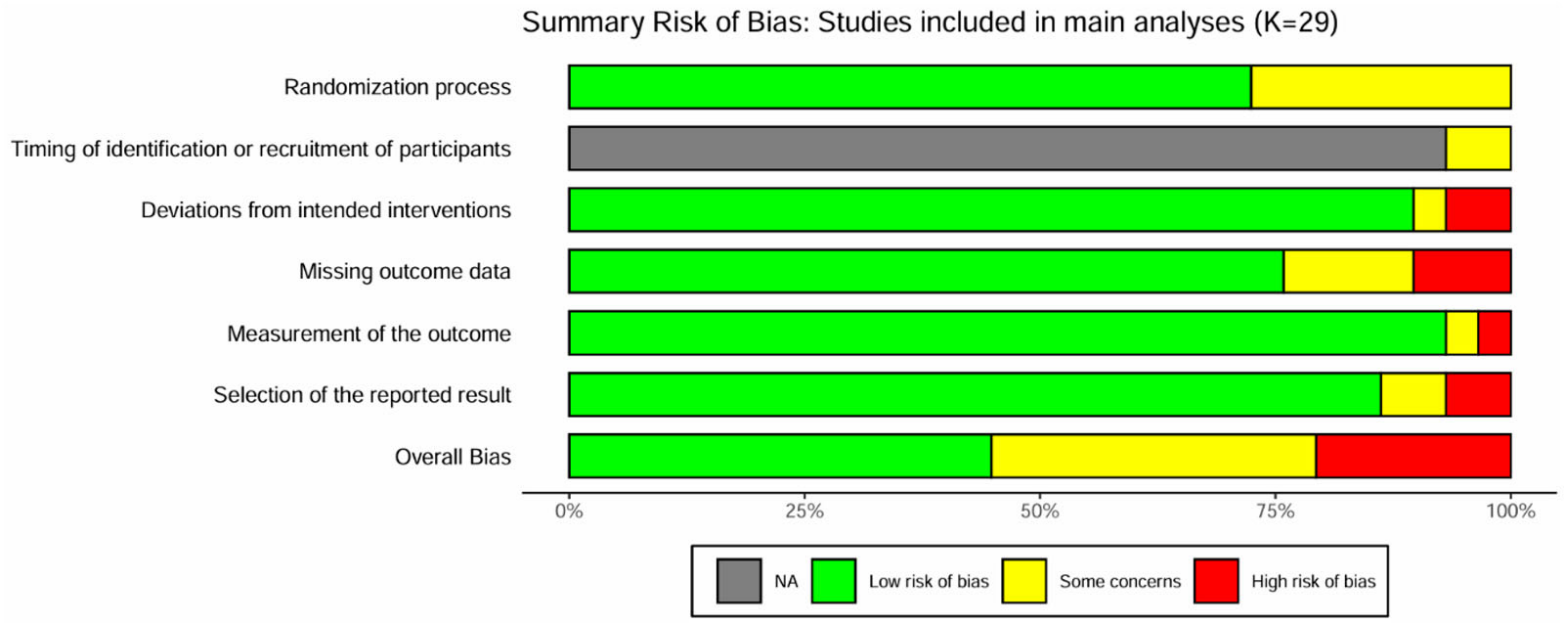
Risk of bias for the studies included in the main analyses. The domain ‘Timing of identification or recruitment of participants’ is only applicable to cluster randomised trials and therefore was relevant for only two of the included studies (as indicated by the grey colour for the remaining studies).

A total of 13 studies (44.8%) were judged to be at low risk of bias, with 10 studies (34.5%) raising some concerns in terms of risk of bias, and 6 studies (20.7%) being judged to be at high risk of bias (Figure 2). A table showing the risk of bias for individual studies and domains can be found in section E of the supplementary files. An overview of the risk of bias for all 48 studies included in the extended analysis can be found in section F of the supplementary files.

#### Intervention characteristics

##### Delivery features

Regarding readability, seven interventions (24.1%) were not in writing (i.e., they used videos or other visual or spoken modalities), so readability was not an applicable measure for them. For the remaining 22 interventions, the mean GFI was 8.06 (SD = 1.41; median = 8.02). In categorical terms, 17 interventions (58.6%) were classified as being of average readability, 4 (13.8%) as being difficult to read, and only one (3.5%) as being easy to read.

Ease-of-use was generally high with a mean score of 6.5 (SD = 1.2; median = 7; range: 4–8) out of 8. Seven interventions (24.1%) met the ease-of-use criteria across all ease-of-use dimensions. All interventions met the ease-of-use criteria in the dimension of Pattern Recognition. Across the remaining seven dimensions, most, but not all studies, met the criteria: Page Names (27; 93.1%), Text Formatting (26; 89.7%), Font Size (25; 86.2%), Clear and Consistent Language (22; 75.9%), Layout (21; 72.4%), Aesthetics (20; 69.0%), and Minimum Text (18; 62.1%).

Modes of delivery were varied. Overall, 14 (48.3%) interventions used more than one mode of delivery. The three most common modes of delivery were exclusively by mobile application (6; 20.7%), exclusively by text messaging (5; 17.2%), and by a combination of website and email (5; 17.2%). A total of 17 interventions (58.6%) were at least partly mobile-phone-based.

##### Intervention content

A list of the individual BCTs used in the intervention and control conditions of each study can be found in section G of the supplementary files. Interventions used a range of BCTs, although some were present in most interventions. The most common BCTs used in the intervention conditions were 1.1 Goal Setting (Behaviour) and 3.1 Social Support (Unspecified) (25; 86.2% each); 5.1 Information about Health Consequences and 11.2 Reduce Negative Emotions (22; 75.8% each); and 4.2 Information about Antecedents (21; 72.4%). The mean number of BCTs in intervention conditions was 26.0 (SD = 14.9, Median = 26, Range = 4–48). The most common BCTs used in the control conditions were similar: 1.1 Goal Setting (Behaviour) (18; 62.1%); 3.1 Social Support (Unspecified) (17; 58.6% each); 11.1 Pharmacological Support (15; 51.7%); 5.1 Information about Health Consequences (13; 44.8%) and 11.2 Reduce Negative Emotions (11; 37.9% each). The mean number of BCTs in control conditions was 10.2 (SD = 10.5, Median = 5, Range = 0–34). The most common BCTs appearing in interventions, but not controls were: 1.2 Problem Solving (15; 51.7%); 5.2 Salience of Consequences (14; 48.3%); 5.6 Information about Emotional Consequences, 6.2 Social Comparison, 9.1 Credible Source, and 13.2 Framing/Reframing (13; 44.8% each). The mean number of BCTs in the intervention, but not control condition was 16.4 (SD = 15.4, Median = 8, Range = −7 to 48). The negative integer indicates that there were more BCTs in the control than in the intervention condition. Histograms showing the distribution of number of BCTs across intervention and control conditions can be found in section H of the supplementary files.

### Meta-analyses

A summary of the findings can be found in Table 3.

**Table 3.** Summary of findings table.

Population	Main analysis (*K* = 29)			Extended analysis (*K* = 48)		
	Effect (95% CI)	*N* ()	Quality of evidence (GRADE)	Effect (95% CI)	*N* (studies)	Quality of evidence (GRADE)
All	OR = 1.29 (95% CI: 1.10–1.51)	*N* = 42,662 (*K* = 29)	Moderate^a^	OR = 1.48 (95% CI: 1.30–1.68)	*N* = 62,606 (*K* = 48)	Moderate^a^
Low SEP	OR = 1.25 (95% CI: 1.00–1.57)	*N* = 24,263 (*K* = 26)	Low^b,c^	OR = 1.53 (95% CI: 1.31–1.80)	*N* = 30,826 (*K* = 46)	Moderate^b^
High SEP	OR = 1.36 (95% CI: 1.06–1.76)	*N* = 18,226 (*K* = 27)	Low^a,b^	OR = 1.43 (95% CI: 1.18–1.74)	*N* = 30,248 (*K* = 41)	Low^a,b^

Notes: Patient or population: adults and adolescents who smoke combustible tobacco Setting: any (community and clinical) Intervention: digital behaviour change intervention delivered in English Comparison: active and inactive comparators.

^a^ Downgraded one level due to statistical heterogeneity.

^b^ Downgraded one level due to indirectness of SEP measure.

^c^ Downgraded one level due to imprecision.

#### Main analyses

**Figure 3.**
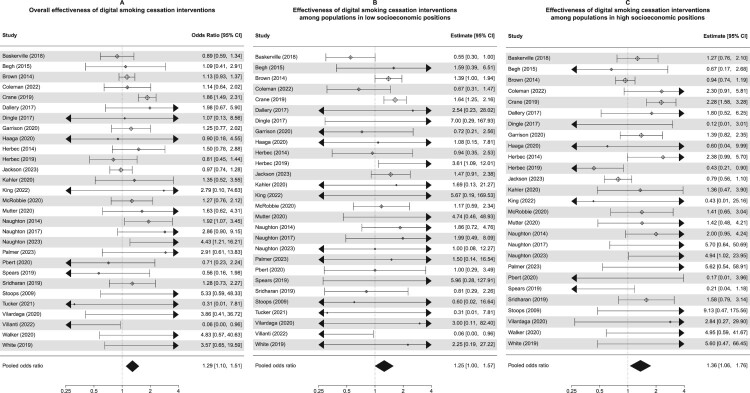
Forest plot showing the individual and pooled effect sizes of the *k* = 29 studies for which authors provided sufficient materials for inclusion in the meta-CART and meta-regressions: overall (A), among populations in low SEPs (B), among populations in high SEPs (C).

The main meta-analysis using only the 29 studies for which we have sufficient information to characterise the intervention in terms of content and delivery features indicates that overall, DCBIs significantly improved abstinence rates (OR = 1.29, 95% CI: 1.10–1.51, *p *= .002; *I*^2^ = 32.7%, *Q*(df = 28) = 45.54, *p *= .019; Figure 3(A)). The main effect of the intervention remained consistent in sensitivity analyses excluding studies deemed at high risk of bias, excluding studies using self-reported abstinence as outcome measure, excluding studies representing outliers in effect size, and excluding studies with low follow-up rates.

When running separate subgroup meta-analyses by SEP, the effect sizes were similar for low (OR = 1.25, 95% CI: 1.00–1.57, *p *= .048; *I*^2^ = 22.3%, *Q*(df = 27) = 30.16, *p *= .307; Figure 3(B)) and high (OR = 1.36, 95% CI: 1.06–1.76, *p *= .017; *I*^2^ = 52.5%, *Q*(df = 26) = 57.74, *p *< .001; Figure 3(C)) SEP. The effect sizes were also similar for studies using an active control (OR = 1.30, 95% CI: 1.06–1.59, *p *= .013; *I*^2^ = 42.7%, *Q*(df = 13) = 23.91, *p *= .032) and inactive control (OR = 1.29, 95% CI: 0.98–1.69, *p *= .069; *I*^2^ = 21.1%, *Q*(df = 14) = 20.69, *p *= .110). Neither SEP (*p *= .659) nor activeness of control (*p *= .958) were significant moderators of effect size in meta-regressions, which is in line with the similarity in effect sizes seen in the subgroup meta-analyses.

Visual inspection of the funnel plot (section I in the supplementary files) and an Egger’s test (*z* = 0.903, *p *= .367) gave no indication of publication bias.

#### Extended set of studies

**Figure 4.**
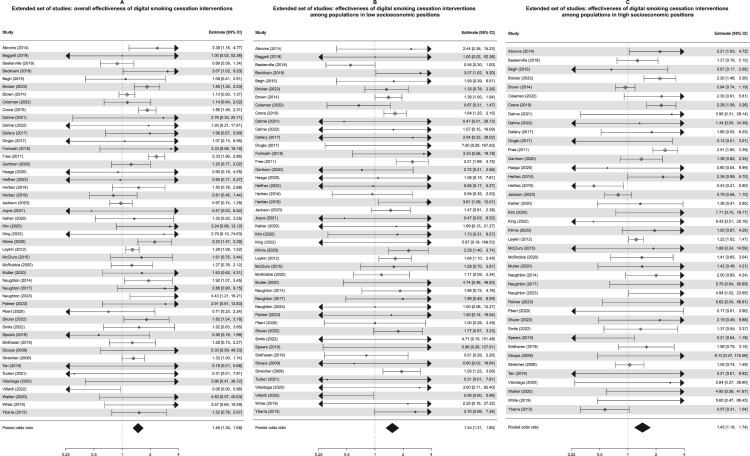
Forest plot showing the individual and pooled effect sizes of the *k* = 48 studies for which authors provided sufficient materials for inclusion in the meta-CART and meta-regressions: overall (A), among populations in low SEPs (B), among populations in high SEPs (C).

When repeating the meta-analyses on the extended set of studies that also includes the 19 studies for which we do not have sufficient materials to characterise the interventions in terms of content and delivery features, the results mirror those of the main analysis. Overall, DCBIs significantly improve quit rates (OR = 1.48, 95% CI: 1.30–1.68, *p *< .001; *I*^2^ = 46.4%, *Q*(df = 47) = 89.95, *p *< .001; Figure 4(A)). This remained consistent in sensitivity analyses excluding studies deemed at high risk of bias, excluding studies using self-reported abstinence as outcome measure, excluding studies representing outliers in effect size, and excluding studies with low follow-up rates.

When running separate subgroup meta-analyses by SEP, the effect sizes were similar for low (OR = 1.53, 95% CI: 1.31–1.80, *p *< .001; *I*^2^ = 23.5%, *Q*(df = 45) = 48.58, *p *= .331; Figure 4(B)) and high (OR = 1.43, 95% CI: 1.18–1.74, *p *< .001; *I*^2^ = 61.5%, *Q*(df = 40) = 100.00, *p *< .001; Figure 4(C)) SEP. The effect sizes were somewhat greater for studies using an inactive (OR = 1.67, 95% CI: 1.34–2.09, *p *< .001; *I*^2^ = 39.8%, *Q*(df = 26) = 49.56, *p *= .004) compared to an active (OR = 1.36, 95% CI: 1.18–1.57, *p *< .001; *I*^2^ = 39.8%, *Q*(df = 20) = 30.89, *p *= .057). However, neither SEP (*p *= .614) nor activeness of control (*p *= .119) were significant moderators of effect size in meta-regressions, which is in line with the findings on the smaller set of studies and the overlapping confidence intervals in the subgroup meta-analyses.

Visual inspection of the funnel plot (section I in the supplementary files) and an Egger’s test (*z* = 0.321, *p *= .748) gave no indication of a publication bias.

### Moderators of effectiveness using meta-CART

Despite substantial heterogeneity in effect sizes (*Q*(df = 54) = 88.81, *p *= .002, *τ*^2^ = 0.102), the main meta-CART analysis did not detect any moderation by SEP, intervention content, or delivery features. This remained consistent across the following sensitivity analyses: entering BCTs as binary categorical variables, using BCT domains instead of individual BCTs as moderators, leaving mode of delivery as a categorical non-dichotomised variable, excluding studies deemed to be at high risk of bias, excluding studies using self-reported abstinence as outcome measure, excluding studies representing outliers in effect size, and excluding studies with high attrition rates. The findings remained consistent when setting the pruning parameter to *c* = 0.5 and *c* = 0.0 to allow for greater power (while controlling the Type 1 error less tightly) and when running the tree separately by SEP. Details of the initial unpruned three and the cross-validation tables of the main meta-CART analysis and the separate trees by SEP can be found in section J of the supplementary files.

### Exploratory meta-regressions

To explore the nature of the null effect found in the main meta-CART analysis, we ran a series of meta-regressions on the overall effect size, and the effect sizes stratified by SEP.

In univariate meta-regressions using a false discovery rate controlled significance level of *p *= .05, only two BCTs, ‘Commitment’ (*b* = 0.49, 95% CI: 0.27–0.71, *p *< .001) and ‘Credible Source’ (*b* = −0.38, 95% CI: −0.61 to −0.15, *p *= .001) were significantly associated with effectiveness. When entering both moderators into a multivariate meta-regression, the effect of ‘Commitment’ remained (*b* = 0.39, 95% CI: 0.09–0.69, *p *= .012), while the effect of ‘Credible Source’ was attenuated (*b* = −0.14, 95% CI: −0.43 to 0.15, *p *= .329).

Exploratory meta-regressions stratified by SEP revealed somewhat divergent results. When running univariate meta-regressions on low SEP effect sizes, no moderators were significant. The association between the BCT ‘Commitment’ and effectiveness is non-significant, but the point estimate is in the same direction (*b* = 0.11, 95% CI: −0.38 to 0.61, *p *= .656). Among high SEP groups, only the BCT ‘Commitment’ (*b* = 0.79, 95% CI: 0.35–1.24, *p *= .001) was associated with effectiveness. Multivariate meta-regressions were therefore not warranted in these stratified meta-regressions. Results from sensitivity analyses entering BCTs into the meta-regressions as binary variables largely mirrored those from the main analyses (see section K of the supplementary files).

## Discussion

DBCIs for smoking cessation improve the odds of smoking abstinence and are equally effective across high and low SEP groups. Interventions tended to be easy to use and used various modes of delivery. However, text-based interventions were largely not easy to read. The DBCIs included in this review used a variety of BCTs, but certain BCTs, such as ‘Goal Setting (Behaviour)’ and ‘Social Support (Unspecified)’, were present in the vast majority of interventions. Delivery mode, ease-of-use, and readability were not significantly associated with intervention effectiveness. Meta-CART analyses did not detect any interactions between potential moderators of effectiveness. Exploratory meta-regression indicated that one BCT, ‘Commitment’ (i.e., asking the participant to (re)affirm pledges to quit smoking), was associated with larger effect sizes, both in general and among populations with high SEP, but not among populations with low SEP.

This review used broader inclusion criteria compared with previous reviews (but see Amiri & Khan, 2023) which have often focussed on particular types of digital interventions such as apps/text messaging (Kingkaew, 2018; Whittaker et al., 2019) or internet-based interventions alone (Graham et al., 2016; McCrabb et al., 2019; Taylor et al., 2017), or specific subpopulations such as pregnant people (Griffiths et al., 2018).

Our finding that, overall and in comparison to inactive controls, DBCIs are effective for increasing smoking cessation rates is in line with previous research (Amiri & Khan, 2023; Graham et al., 2016; Griffiths et al., 2018; Kingkaew, 2018; Liu et al., 2023; McCrabb et al., 2019; Whittaker et al., 2019). However, unlike some previous reviews, we also found evidence that DBCIs are effective when compared to active controls (Graham et al., 2016; Griffiths et al., 2018; Taylor et al., 2017; Whittaker et al., 2019).

Previous research on the equity impact of DBCIs for smoking cessation has been equivocal. Some previous research has indicated that quit success is lower among smokers in lower SEP (Kotz & West, 2009; Reid et al., 2010) and that smoking cessation interventions may be less effective for smokers in low SEPs (T. Brown et al., 2014; Hiscock et al., 2015). However, a previous observational population study has indicated that websites in particular may potentially be (more) effective for smoking cessation for people in low SEPs (Jackson et al., 2019). We found that DBCIs are effective for populations in both high and low SEPs and no indication for differences in effectiveness by SEP. Our review also extends the findings from two previous reviews. One previous review of individual-level behavioural smoking cessation interventions delivered through various means found that overall, these interventions are about equally effective for participants with high and low SEP (Kock et al., 2019). Another review found targeted technology-mediated interventions are effective for disadvantaged groups (Boland et al., 2018). Our review extends these findings to non-targeted DBCIs and by exploring whether SEP, intervention, or delivery features moderate the effectiveness of DBCIs. While our review finds no evidence of that DBCIs are less effective in helping smokers in low SEPs quit, they might still contribute to health inequalities if there are disparities in access to them. Although rates of smartphone ownership and home internet access are increasing, a digital divide still persists, with those who are younger, in higher SEPs, and residents in higher income countries having higher rates of internet access and smartphone ownership (Schumacher & Kent, 2020).

In moderator analyses, positive associations between BCT ‘Commitment’ and effectiveness were observed in the overall population and when restricting the analyses to high SEP populations. This is not in line with previous research, which has not shown a link between this BCT and effectiveness in DBCIs for smoking cessation but has found associations between other BCTs and effectiveness (Griffiths et al., 2018; Kingkaew, 2018; McCrabb et al., 2019). However, previous studies of this type have also been inconsistent with each other. A reason for this may be that the reviews have used different inclusion criteria for studies (in terms of intervention type, study type, population), potential moderators of effect size, and data extraction and meta-analytic methods. Interpretation of the finding that the BCT ‘Commitment’ significantly moderates effectiveness in the high SEP group only is complicated by the fact that the meta-CART analyses did not find evidence that SEP moderates the effectiveness of the BCT ‘Commitment’. Additionally, the confidence intervals of the moderation effect estimates of ‘Commitment’ in the meta-regressions using high and low SEP samples overlap, suggesting that while the effect of ‘Commitment’ is only statistically significant in the high SEP group, the difference between the effects in the high and low SEP groups may not in itself be significant (Gelman & Stern, 2006). However, the differential findings of its impact on effectiveness when running SEP-separated meta-regressions are nevertheless interesting. It suggests that it is possible that different intervention aspects may be differentially effective for different populations. It also provides further support for the idea that SEP shapes the kind of support people need or would benefit from most when it comes to smoking cessation. A potential reason why high SEP populations may particularly benefit from interventions utilising the BCT ‘Commitment’ is the socioeconomic pattering of smoking-related social norms and beliefs. The mechanism of action through which ‘Commitment’ is believed to aid behaviour change is by highlighting and linking the behaviour to certain principles and values that outline what is good, desirable, and important for someone and their relevant others (Carey et al., 2019). Previous research has highlighted that anti-smoking norms and high smoking risk perceptions are more prevalent in more socioeconomically advantaged communities (Hiscock et al., 2012; Huisman et al., 2012; van Wijk et al., 2019). Therefore, reaffirming one’s commitment not to smoke may be of greater relevance for people in high SEPs.

The finding that the ease-of-use of DBCIs for smoking cessation is high is in line with previous research on the ease-of-use features of apps (Ubhi, Kotz, et al., 2016). Furthermore, there is some overlap with the most common BCTs identified in this study and those identified in previous studies, such as the prevalence of the BCTs ‘Goal Setting (Behaviour)’, ‘Social Support (Unspecified)’, ‘Information about Antecedents’, and ‘Information about Health Consequences’ (Griffiths et al., 2018; Kingkaew, 2018; McCrabb et al., 2019). The finding that DBCIs for smoking cessation are overwhelmingly not easy to read is in line with previous research indicating low readability of smoking cessation apps and websites (Bock et al., 2004; Ferron et al., 2017). We are not aware of any previous research characterising DBCIs for smoking cessation in terms of how they map onto the mode of delivery ontology (Marques et al., 2020), which means that we cannot gauge to what extent the modes of delivery used in the interventions included in this review are reflective of any trends or changes over time.

The null finding of the current meta-CART analysis and the limited associations between intervention features and effectiveness might represent a genuine lack of a relationship, but it might also be the result of insufficient statistical power. While meta-CART analyses have greater statistical power than conventional multivariate meta-analyses, it is possible that *k* = 55 effect sizes are insufficient to find a genuine effect in our sample. A previous simulation study has shown that the recovery rate/power improves with more studies, if more/all moderators are binary, and that although a larger number of moderators do not drastically reduce the power, it is still somewhat reduced (Li et al., 2019). Another potential reason for the null finding might be that our measures of intervention features were insufficiently detailed. A major limitation of the BCTTv1 is that there is no way to control for the ‘dose’ of BCTs delivered. However, the intensity of a BCT is likely to differ within and between studies. Our coding of BCTs as −1 if present in control, but not intervention, 0 if present in both control and intervention or neither, and 1 if present in intervention, but not control and entering that into the analysis as a continuous/numeric variable implicitly assumes that BCTs are equivalent regardless of whether they occur in the control or intervention condition. However, this is not always the case. For many studies, even when there was a large overlap in the BCTs delivered in the intervention and control groups, the intervention group tended to receive a more intensive version of the BCT. For example, one study compared a personalised and tailored interactive website to a static control website (J. Brown et al., 2014). Both the intervention and the control website contained the BCT ‘Pharmacological Support’ (i.e., supplying or promoting use of nicotine replacement therapy or other smoking cessation medication). However, the intervention website contained much more extensive descriptions outlining the pros and cons of different forms of pharmacotherapy and more detailed descriptions of how to use them. In another study, which compared two apps, both the intervention and the control app contained the BCT ‘Distraction’ (i.e., recommend or provide distraction from potential behavioural cues) (Baskerville et al., 2018). However, the intervention app had an integration with social media and integrated interactive social distractions, while the control app merely advised using an alternative focus for attention.

Moreover, our approach to coding intervention features was unable to account for the way that participants actually engaged with the intervention. Previous studies investigating usage of DBCIs found that users of may pick and choose to use only specific features of interventions (Morrison et al., 2018). Participants in the reviewed studies might have been able to pick out content that they needed or was useful for them from the intervention even when the interventions were not highly useable or easy to read, or contained content that was inaccessible or irrelevant to them. This might also explain why the interventions appeared to be equally effective across socioeconomic groups despite their low readability. A third potential reason for the null findings could be a failure to account for confounding factors. There are likely to be remaining unmeasured confounds and intervention features that could not be fully accounted for by using random-effects models.

In addition to the above-mentioned issues, there are other limitations of this review. The measures used to operationalise readability and ease-of-use are limited. The ease-of-use taxonomy may apply unequally across intervention platforms and has so far only been validated for apps (Ubhi, Kotz, et al., 2016; Ubhi, Michie, et al., 2016). Additionally, judgements for dimensions such as aesthetics are likely influenced by the current aesthetic norms of digital media and platforms. Although the coders tried to account for this, they were probably unable to fully equivalise this measure. Additionally, some intervention features which might influence effectiveness such as personalisation were not measured. Furthermore, the commensurability between different measures of SEP or even the nominally same measure of SEP across different countries and times is likely to be limited. A further limitation of this review is its limited generalisability. Since, for pragmatic reasons, only studies evaluating English-language interventions were included, most studies included in this review were conducted in Western, predominantly English-speaking countries, and particularly the UK and the US. Therefore, these findings might not generalise to other populations. Additionally, only authors of 29 out of 197 studies (14.7%) provided sufficient data and intervention materials to estimate differential effect size by SEP and allow for the coding of relevant intervention features. While for 48 out of 197 studies (24.4%), authors provided sufficient data to estimate differential effect size by SEP. While this response rate was in line with our expectations, it is still fairly low. It likely also contributes to a selection bias because authors of older or lower quality studies may have been less likely to provide data and materials.

In terms of implications, this review provides further evidence that DBCIs are a promising modality for smoking cessation for people from across socioeconomic strata. The similarity in effectiveness by SEP means DBCIs for smoking cessation may be able to reduce health inequalities if they can be rolled out in an equitable manner. However, insight into the active ingredients of these interventions remains limited. Our evidence suggests that high SEP populations may particularly benefit from the inclusion of the BCT ‘Commitment’. This review does highlight that future research on DBCIs for smoking cessations might benefit from factorial designs to identify and establish causal links between intervention features and effectiveness more clearly. This may aid in our understanding of how and why DBCIs for smoking cessation work.

## Conclusions

In conclusion, this review suggests that DBCIs for smoking cessation are effective across socioeconomic groups, although there is some indication based on exploratory analyses that specific BCTs may be more or less effective depending on the target population’s SEP. It also highlights that the currently available research literature offers only limited insights into the active ingredients of DBCIs for smoking cessation, how they interact with each other, and how they are moderated by the target population.

## Supplementary Material

Supplemental Material

## Data Availability

Data and syntax used for analysis are available on OSF (https://osf.io/4cgdb/) once the results are published.
