# Supplementary material for: Does socioeconomic position moderate the associations between the content and delivery features of digital behaviour change interventions for smoking cessation and intervention effectiveness? A systematic review and meta-analysis
Source: Health Psychol Rev. 2024 Jun 18;18(4):790–823. doi: 10.1080/17437199.2024.2366189 (PMC11614050; doi:10.1080/17437199.2024.2366189)
Supplement: Supplemental Material [file RHPR_A_2366189_SM0655.pdf]

Supplementary Files

A. Search strategy

A.1. MEDLINE (Ovid)

1. cell phone* or cellular phone* or mobile phone* or mobile device* or smart device* or phone-based or phonebased or smartphone*).tw.
2. (app or apps).tw.
3. (telecommunication or telehealth or telemedicine).tw.
4. (ehealth or e health).tw.
5. (internet or online or on line).tw.
6. digital*.tw.
7. (computer* or laptop*).tw.
8. (iPad* or (tablet adj5 (internet or computer or digital or mobile or internet or electronic or cellular or web or online or on line or smart* or mobile*))).tw.
9. (m health or mhealth).tw.
10. (u health or uhealth).tw.
11. (email or e mail or electronic mail).tw.
12. (text messag* or SMS).tw.
13. (chat-based or text-based).tw.
14. (multimedia messag* or MMS).tw.
15. social media.tw.
16. cell phone/ or smartphone/ or text messaging/
17. microcomputers/ or computers, handheld/ or smartphone/ or smart glasses/ or minicomputers/
18. gamification/ or mobile applications/ or video games/
19. Social Media/
20. or/1-19
21. ((quit* or stop* or give* or ceas* or cess*) adj3 (smok* or tobacco* or cigar* or pipe* or shisha* or hookah* or nargile* or narghile* or waterpipe*)).tw.
22. smoking cessation/ or "tobacco use cessation"/
23. "Tobacco Use Disorder"/
24. or/21-23
25. (compare or compared or comparison).ti.
26. randomized controlled trial.pt.
27. controlled clinical trial.pt.
28. placebo.ti,ab.
29. drug therapy.fs.
30. random*.ti,ab.
31. controlled clinical trial/ or randomized controlled trial/ or equivalence trial/ or pragmatic clinical trial/ or comparative study/
32. groups.ti,ab.
33. trial.ti,ab.
34. experiment*.ti,ab.
35. quasiexperiment*.ti,ab.

Formatted: Line spacing: At least 1.15 pt

Formatted: Indent: Left: 0.63 cm, Hanging: 0.63 cm, Line spacing: Multiple 1.15 li

36. (pilot adj2 stud*).ti,ab.
37. (feasib* adj2 stud*).ti,ab.
38. or/25-37
39. exp animals/ not humans.sh.
40. 38 not 39
41. 20 and 24 and 40
42. limit 41 to yr="2004 -Current"
43. limit 41 to (english language or no language specified)

A.2. PsycINFO (Ovid)

Formatted: Line spacing: At least 1.15 pt

1. (cell phone* or cellular phone* or mobile phone* or mobile device* or smart device* or smartphone*).tw.
2. (app or apps).tw.
3. (telecommunication or telehealth or telemedicine or tele-communication or tele-health or tele-medicine).tw.
4. (ehealth or e health).tw.
5. (internet or online or on line).tw.
6. digital*.tw.
7. (computer* or laptop*).tw.
8. (iPad* or (tablet* adj5 (internet or computer* or digital or mobile or internet or electronic or cellular or web or online or on line or smart* or mobile*))).tw.
9. (m health or mhealth).tw.
10. (u health or uhealth).tw.
11. (email or e mail or electronic mail).tw.
12. (text messag* or SMS).tw.
13. (chat-based or text-based).tw.
14. (multimedia messag* or MMS).tw.
15. social media.tw.
16. digital technology/ or computer applications/ or computer games/ or digital marketing/ or digital video/ or electronic communication/ or mobile technology/ or wireless technologies/ or digital media/ or digital mental health resources/ or electronic health services/ or human technology interaction/ or wearable devices/
17. exp mobile devices/
18. mobile applications/ or computer applications/ or digital gaming/ or electronic communication/ or mobile health/ or mobile learning/ or mobile phones/ or smartphones/ or tablet computers/ or mobile assessment/
19. social media/ or electronic communication/ or online social networks/ or blog/ or computer mediated communication/ or digital marketing/ or digital storytelling/ or internet/ or social interaction/ or websites/
20. or/1-19
21. ((quit* or stop* or give* or ceas* or cess*) adj3 (smok* or tobacco* or cigar* or pipe* or shisha* or hookah* or nargile* or narghile* or waterpipe*)).tw.
22. smoking cessation/
23. or/21-22
24. exp clinical trials/ or exp clinical trial/ or exp controlled clinical trials/
25. exp crossover procedure/ or exp cross over studies/ or exp crossover design/
26. exp double blind procedure/ or exp double blind method/ or exp double blind studies/ or exp single blind procedure/ or exp single blind method/ or exp single blind studies/
27. exp random allocation/ or exp randomization/ or exp random assignment/ or exp random sample/ or exp random sampling/

28. exp randomized controlled trials/ or exp randomized controlled trial/ or randomized controlled trials as topic/
29. (clinical adj2 trial$).tw.
30. (crossover or cross over).tw.
31. (((single$ or doubl$ or trebl$ or tripl$) adj5 (blind$ or mask$ or dummy)) or (singleblind$ or doubleblind$ or trebleblind$)).tw.
32. (placebo$ or random$).mp.
33. (clinical trial$ or random$).pt. or treatment outcome$.md.
34. experiment*.ti,ab.
35. quasiexperiment*.ti,ab.
36. (pilot adj2 stud*).ti,ab.
37. (feasib* adj2 stud*).ti,ab.
38. or/24-37
39. animals/ not (animals/ and human$.mp.)
40. (animal/ or animals/) not ((animal/ and human/) or (animals/ and humans/))
41. (animal not (animal and human)).po.
42. or/39-41
43. 38 not 42
44. 20 and 23 and 43
45. limit 44 to yr="2004 -Current"
46. limit 44 to (english language or no language specified)
47. 45 and 46

A.3. EMABSE (Ovid)

Formatted: Line spacing: At least 1.15 pt

1. (cell phone* or cellular phone* or mobile phone* or mobile device* or smart device* or smartphone*).tw.
2. (app or apps).tw.
3. (telecommunication or telehealth or telemedicine or tele-communication or tele-health or tele-medicine).tw.
4. (ehealth or e health).tw.
5. (internet or online or on line).tw.
6. digital*.tw.
7. (computer* or laptop*).tw.
8. (iPad* or (tablet* adj5 (internet or computer* or digital or mobile or internet or electronic or cellular or web or online or on line or smart* or mobile*))).tw.
9. (m health or mhealth).tw.
10. (u health or uhealth).tw.
11. (email or e mail or electronic mail).tw.
12. (text messag* or SMS).tw.
13. (chat-based or text-based).tw.
14. (multimedia messag* or MMS).tw.
15. social media.tw.
16. mobile phone/
17. smartphone/
18. microcomputer/
19. personal digital assistant/
20. mobile application/
21. social media/
22. or/1-21
23. ((quit* or stop* or give* or ceas* or cess*) adj3 (smok* or tobacco* or cigar* or pipe* or shisha* or hookah* or nargile* or narghile* or waterpipe*)).tw.
24. smoking cessation/

25. smoking cessation program/
26. tobacco dependence/
27. or/23-26
28. Randomized controlled trial/
29. Controlled clinical study/
30. Random$.ti,ab.
31. randomization/
32. intermethod comparison/
33. placebo.ti,ab.
34. (compare or compared or comparison).ti.
35. (open adj label).ti,ab.
36. ((double or single or doubly or singly) adj (blind or blinded or blindly)).ti,ab.
37. double blind procedure/
38. parallel group$1.ti,ab.
39. (crossover or cross over).ti,ab.
40. ((assign$ or match or matched or allocation) adj5 (alternate or group$1 or intervention$1 or patient$1 or subject$1 or participant$1)).ti,ab.
41. (assigned or allocated).ti,ab.
42. (controlled adj7 (study or design or trial)).ti,ab.
43. experiment*.ti,ab.
44. quasiexperiment*.ti,ab.
45. (pilot adj2 stud*).ti,ab.
46. (feasib* adj2 stud*).ti,ab.
47. or/28-46
48. (exp animal/ or animal.hw. or nonhuman/) not (exp human/ or human cell/ or (human or humans).ti.)
49. 47 not 48
50. 22 and 27 and 49
51. limit 50 to yr="2004 -Current"
52. limit 50 to (english language or no language specified)
53. 51 and 52

A.4. CINAHL

S1: ((TI "cell phone*" OR AB "cell phone*") OR (TI "cellular phone*" OR AB "cellular phone*") OR (TI "mobile phone*" OR AB "mobile phone*") OR (TI "mobile device*" OR AB "mobile device*") OR (TI "smart device*" OR AB "smart device*") OR (TI phone-based OR AB phone-based) OR (TI phonebased OR AB phonebased) OR (TI smartphone* OR AB smartphone*))

S2: ((TI app OR AB app) OR (TI apps OR AB apps))

S3: ((TI telecommunication OR AB telecommunication) OR (TI telehealth OR AB telehealth) OR (TI telemedicine OR AB telemedicine) OR (TI "tele communication" OR AB "tele communication") OR (TI "tele health" OR AB "tele health") OR (TI "tele medicine" OR AB "tele medicine"))

S4: ((TI ehealth OR AB ehealth) OR (TI "e health" OR AB "e health"))

S5: ((TI internet OR AB internet) OR (TI online OR AB online) OR (TI "on line" OR AB "on line"))

S6: (TI digital* OR AB digital*)

S7: ((TI computer* OR AB computer*) OR (TI laptop* OR AB laptop*))

S8: ((TI iPad* OR AB iPad*) OR ((TI tablet* OR AB tablet*) N5 ((TI internet OR AB internet) OR (TI computer* OR AB computer*) OR (TI digital OR AB digital) OR (TI mobile OR AB mobile) OR (TI internet OR AB internet) OR (TI electronic OR AB electronic) OR (TI cellular OR AB cellular) OR (TI web

OR AB web) OR (TI online OR AB online) OR (TI "on line" OR AB "on line") OR (TI smart* OR AB smart*) OR (TI mobile* OR AB mobile*))))

S9: ((TI "m health" OR AB "m health") OR (TI mhealth OR AB mhealth))

S10: ((TI "u health" OR AB "u health") OR (TI uhealth OR AB uhealth))

S11: ((TI email OR AB email) OR (TI "e mail" OR AB "e mail") OR (TI "electronic mail" OR AB "electronic mail"))

S12: ((TI "text messag*" OR AB "text messag*") OR (TI SMS OR AB SMS))

S13: ((TI chat-based OR AB chat-based) OR (TI text-based OR AB text-based))

S14: ((TI "multimedia messag*" OR AB "multimedia messag*") OR (TI MMS OR AB MMS))

S15: (TI "social media" OR AB "social media")

S16: (MH "Cellular Phone+") OR (MH "Text Messaging+") OR (MH "Smartphone")

S17: (MH "Microcomputers+") OR (MH "Computers, Portable+") OR (MH "IBM Compatible Microcomputers") OR (MH "Macintosh Microcomputers") OR (MH "Smart Glasses")

S18: (MH "Gamification")

S19: (MH "Mobile Applications") OR (MH "Patient Portals")

S20: (MH "Video Games+") OR (MH "Virtual Reality+") OR (MH "Virtual Reality Exposure Therapy")

S21: (MH "Internet+") OR (MH "Email") OR (MH "Internet Access") OR (MH "Internet-Based Intervention") OR (MH "Social Media+") OR (MH "Facebook") OR (MH "Twitter") OR (MH "World Wide Web Applications")

S22: S1 OR S2 OR S3 OR S4 OR S5 OR S6 OR S7 OR S8 OR S9 OR S10 OR S11 OR S12 OR S13 OR S14 OR S15 OR S16 OR S17 OR S18 OR S19 OR S20 OR S21

S23: (((TI quit* OR AB quit*) OR (TI stop* OR AB stop*) OR (TI give* OR AB give*) OR (TI ceas* OR AB ceas*) OR (TI cess* OR AB cess*)) N3 ((TI smok* OR AB smok*) OR (TI tobacco* OR AB tobacco*) OR (TI cigar* OR AB cigar*) OR (TI pipe* OR AB pipe*) OR (TI shisha* OR AB shisha*) OR (TI hookah* OR AB hookah*) OR (TI nargile* OR AB nargile*) OR (TI narghile* OR AB narghile*) OR (TI waterpipe* OR AB waterpipe*)))

S24: (MH "Smoking Cessation") OR (MH "Smoking Cessation Programs") OR (MH "Tobacco Use Cessation Products+")

S25: S23 OR S24

S26: MH randomized controlled trials

S27: MH double-blind studies

S28: MH single-blind studies

S29: MH random assignment

S30: MH pretest-posttest design

S31: MH cluster sample

S32: TI (randomised OR randomized)

S33: AB (random*)

S34: TI (trial)

S35: MH (sample size) AND AB (assigned OR allocated OR control)

S36: MH (placebos)

S37: PT (randomized controlled trial)

S38: AB (control W5 group)

S39: MH (crossover design) OR MH (comparative studies)

S40: AB (cluster W3 RCT)

S41: (TI experiment* OR AB experiment*)

S42: (TI quasiexperiment* OR AB quasiexperiment*)

S43: ((TI pilot OR AB pilot) N2 (TI stud* OR AB stud*))

S44: ((TI feasib* OR AB feasib*) N2 (TI stud* OR AB stud*))

S45: S26 OR S27 OR S28 OR S29 OR S30 OR S31 OR S32 OR S33 OR S34 OR S35 OR S36 OR S37 OR S38 OR S39 OR S40 OR S41 OR S42 OR S43 OR S44

S46: MH animals+

S47: MH (animal studies)

S48: TI (animal model*)

S49: S46 OR S47 OR S48

S50: MH (human)

S51: S49 not S50

S52: S45 not S51

S53:S22 AND S25 AND S52 Limiters - Publication Year: 2004-

A.5. ASSIA

((TI(compare) OR TI(compared) OR TI(comparison)) OR NOFT("randomized controlled trial") OR NOFT("controlled clinical trial") OR TI,AB(placebo) OR "Drug Therapy" OR TI,AB(random*) OR MESH.EXACT("controlled clinical trial") OR MESH.EXACT("randomized controlled trial") OR MESH.EXACT("equivalence trial") OR MESH.EXACT("pragmatic clinical trial") OR MESH.EXACT("comparative study") OR TI,AB(groups) OR TI,AB(trial) OR TI,AB(experiment*) OR TI,AB(quasiexperiment*) OR (TI,AB(pilot) NEAR/2 TI,AB(stud*)) OR (TI,AB(feasib*) NEAR/2 TI,AB(stud*)) OR RCT) AND (((TI,AB(quit*) OR TI,AB(stop*) OR TI,AB(give*) OR TI,AB(ceas*) OR TI,AB(cess*)) NEAR/3 (TI,AB(smok*) OR TI,AB(tobacco*) OR TI,AB(cigar*) OR TI,AB(pipe*) OR TI,AB(shisha*) OR TI,AB(hookah*) OR TI,AB(nargile*) OR TI,AB(narghile*) OR TI,AB(waterpipe*))) OR (SU("smoking cessation") OR SU("tobacco use cessation")) OR SU("Tobacco Use Disorder")) AND (((TI,AB(("cell phone")) OR TI,AB(("cellular phone" OR "cellular phones")) OR TI,AB(("mobile phone" OR "mobile phones")) OR TI,AB(("mobile device" OR "mobile devices")) OR TI,AB(("smart device" OR "smart devices")) OR TI,AB(phone-based) OR TI,AB(phonebased) OR TI,AB(smartphone*)) OR (TI,AB(app) OR TI,AB(apps)) OR (TI,AB(telecommunication) OR TI,AB(telehealth) OR TI,AB(telemedicine) OR TI,AB(tele-communication) OR TI,AB(tele-health) OR TI,AB(tele-medicine)) OR (TI,AB(ehealth) OR TI,AB("e health")) OR (TI,AB(internet) OR TI,AB(online) OR TI,AB("on line")) OR TI,AB(digital*) OR (TI,AB(computer*) OR TI,AB(laptop*)) OR (TI,AB(iPad*) OR (TI,AB(tablet) NEAR/5

(TI,AB(internet) OR TI,AB(computer) OR TI,AB(digital) OR TI,AB(mobile) OR TI,AB(internet) OR TI,AB(electronic) OR TI,AB(cellular) OR TI,AB(web) OR TI,AB(online) OR TI,AB("on line") OR TI,AB(smart*) OR TI,AB(mobile*)))) OR (TI,AB("m health") OR TI,AB(mhealth)) OR (TI,AB("u health") OR TI,AB(uhealth))) OR ((TI,AB(email) OR TI,AB("e mail") OR TI,AB("electronic mail")) OR (TI,AB(("text message" OR "text messages" OR "text messaging")) OR TI,AB(SMS)) OR (TI,AB(chat-based) OR TI,AB(text-based)) OR (TI,AB(("multimedia messages" OR "multimedia messaging")) OR TI,AB(MMS)) OR TI,AB("social media")) OR ((SU("cell phone") OR SU(smartphone) OR SU("text messaging")) OR (SU(microcomputers) OR SU("computers, handheld") OR SU(smartphone) OR SU("smart glasses") OR SU(minicomputers)) OR (SU(gamification) OR SU("mobile applications") OR SU("video games")) OR SU("Social Media"))) AND pd(>20031231)

A.6. Web of Science Core Collection

#1: TS=(compare OR compared OR comparison OR trial OR placebo OR random* OR groups OR experiment* OR quasiexperiment* OR (pilot NEAR/2 stud*) OR (feasib* NEAR/2 stud*) OR RCT)

#2: TS=((quit* OR stop* OR give* OR ceas* OR cess*) NEAR/3 (smok* OR tobacco* OR cigar* OR pipe* OR shisha* OR hookah* OR nargile* OR narghile* OR waterpipe*))

#3: TS=("cell phone" OR "cellular phone" OR "cellular phones" OR "mobile phone" OR "mobile phones" OR "mobile device" OR "mobile devices" OR "smart device" OR "smart devices" OR phone-based OR phonebased OR smartphone* OR app OR apps OR telecommunication OR telehealth OR telemedicine OR tele-communication OR tele-health OR tele-medicine OR ehealth OR "e health" OR internet OR online OR "on line" OR digital* OR computer* OR laptop* OR iPad* OR ((tablet) NEAR/5 (internet OR computer OR digital OR mobile OR internet OR electronic OR cellular OR web OR online OR "on line" OR smart* OR mobile*)) OR "m health" OR mhealth OR "u health" OR uhealth OR email OR "e mail" OR "electronic mail" OR "text message" OR "text messages" OR "text messaging" OR SMS OR chat-based OR text-based OR "multimedia messages" OR "multimedia messaging" OR MMS OR "social media") Timespan: 2003-12-31 to 2023-02-21

#4: #3 AND #2 AND #1 Timespan: 2003-12-31 to 2023-02-21

A.7. ACM Digital Library

(Abstract:((((quit* OR stop* OR give* OR ceas* OR cess*) AND (smok* OR tobacco* OR cigar* OR pipe? OR shisha* OR hookah* OR nargile*)) OR "smoking cessation" OR "tobacco use cessation" OR "Tobacco Use Disorder")) OR Title:((((quit* OR stop* OR give* OR ceas* OR cess*) AND (smok* OR tobacco* OR cigar* OR pipe? OR shisha* OR hookah* OR nargile*)) OR "smoking cessation" OR "tobacco use cessation" OR "Tobacco Use Disorder"))) AND ((Title:((compare OR compared OR comparison) OR "randomized controlled trial" OR "controlled clinical trial" OR placebo OR "Drug Therapy" OR random* OR "controlled clinical trial" OR "randomized controlled trial" OR "equivalence trial" OR "pragmatic clinical trial" OR "comparative study" OR groups OR trial OR experiment* OR quasiexperiment* OR (pilot AND stud*) OR (feasib* AND stud*))) OR Abstract:(((compare OR compared OR comparison) OR "randomized controlled trial" OR "controlled clinical trial" OR placebo OR "Drug Therapy" OR random* OR "controlled clinical trial" OR "randomized controlled trial" OR "equivalence trial" OR "pragmatic clinical trial" OR "comparative study" OR groups OR trial OR experiment* OR quasiexperiment* OR (pilot AND stud*) OR (feasib* AND stud*)))) AND ((Title:(("cell phone*" OR "cellular phone*" OR "mobile phone*" OR "mobile device*" OR "smart device*" OR phone-based OR phonebased OR smartphone*) OR (app OR apps) OR (telecommunication OR telehealth OR telemedicine OR "tele communication" OR "tele health" OR "tele medicine") OR (ehealth OR "e health") OR (internet OR online OR "on line") OR digital* OR (computer* OR laptop*) OR (iPad* OR (tablet* AND (internet OR computer* OR digital OR mobile OR internet OR electronic OR cellular OR web OR online OR "on line" OR smart* OR mobile*))) OR ("m health" OR mhealth) OR ("u health" OR uhealth) OR (email OR "e mail" OR "electronic mail") OR ("text messag*" OR SMS) OR (chat-based OR text-based) OR ("multimedia messag*" OR MMS) OR "social media" OR "cell phone"

OR smartphone OR "text messaging" OR microcomputers OR "computers, handheld" OR smartphone OR "smart glasses" OR minicomputers OR gamification OR "mobile applications" OR "video games" OR "Social Media")) OR Abstract:(("cell phone*" OR "cellular phone*" OR "mobile phone*" OR "mobile device*" OR "smart device*" OR phone-based OR phonebased OR smartphone*) OR (app OR apps) OR (telecommunication OR telehealth OR telemedicine OR "tele communication" OR "tele health" OR "tele medicine") OR (ehealth OR "e health") OR (internet OR online OR "on line") OR digital* OR (computer* OR laptop*) OR (iPad* OR (tablet AND (internet OR computer OR digital OR mobile OR internet OR electronic OR cellular OR web OR online OR "on line" OR smart* OR mobile*))) OR ("m health" OR mhealth) OR ("u health" OR uhealth) OR (email OR "e mail" OR "electronic mail") OR ("text messag*" OR SMS) OR (chat-based OR text-based) OR ("multimedia messag*" OR MMS) OR "social media" OR "cell phone" OR smartphone OR "text messaging" OR microcomputers OR "computers, handheld" OR smartphone OR "smart glasses" OR minicomputers OR gamification OR "mobile applications" OR "video games" OR "Social Media")))

A.8. IEEE Digital Library

((("All Metadata":quit OR "All Metadata":quitting OR "All Metadata":stop OR "All Metadata":stopping OR "All Metadata":give OR "All Metadata":giving OR "All Metadata":cease OR "All Metadata":ceasing OR "All Metadata":cessation OR "All Metadata":dependent OR "All Metadata":dependence) NEAR/3 ("All Metadata":smoke OR "All Metadata":smoking OR "All Metadata":tobacco OR "All Metadata":cigar* OR "All Metadata":pipe OR "All Metadata":pipes OR "All Metadata":shisha OR "All Metadata":shishas OR "All Metadata":hookah OR "All Metadata":hookahs OR "All Metadata":nargile OR "All Metadata":nargiles OR "All Metadata":narghile OR "All Metadata":narghiles OR "All Metadata":waterpipe OR "All Metadata":waterpipes)))

You Refined By: Year: 2004-2023

A.9. Cochrane Central Register of Controlled Trials (CENTRAL)

#1 ("cell phone*" OR "cellular phone*" OR "mobile phone*" OR "mobile device*" OR "smart device*" OR phone-based OR phonebased OR smartphone*):ti,ab,kw

#2 (app OR apps):ti,ab,kw

#3 (telecommunication OR telehealth OR telemedicine OR "tele-medicine" OR "tele-communication" OR "tele-health"):ti,ab,kw

#4 (ehealth OR "e health"):ti,ab,kw

#5 (internet OR online OR "on line"):ti,ab,kw

#6 (digital*):ti,ab,kw

#7 (computer* OR laptop*):ti,ab,kw

#8 (iPad*):ti,ab,kw

#9 (tablet* NEAR/4 (internet OR computer* OR digital OR mobile OR internet OR electronic OR cellular OR web OR online OR "on line" OR smart* OR mobile*)):ti,ab,kw

#10 ("m health" OR mhealth):ti,ab,kw

#11 ("u health" OR uhealth):ti,ab,kw

#12 (email OR "e mail" OR "electronic mail"):ti,ab,kw

#13 ("text messag*" OR SMS):ti,ab,kw

#14 (chat-based OR text-based):ti,ab,kw

#15 ("multimedia messag*" OR MMS):ti,ab,kw

#16 ("social media"):ti,ab,kw

#17 MeSH descriptor: [Cell Phone] explode all trees

#18 MeSH descriptor: [Smartphone] explode all trees

#19 MeSH descriptor: [Text Messaging] explode all trees

#20 MeSH descriptor: [Microcomputers] explode all trees

#21 MeSH descriptor: [Computers, Handheld] explode all trees

#22 MeSH descriptor: [Smart Glasses] explode all trees

#23 MeSH descriptor: [Virtual Reality] explode all trees

#24 MeSH descriptor: [Minicomputers] explode all trees

#25 MeSH descriptor: [Gamification] explode all trees

#26 MeSH descriptor: [Mobile Applications] explode all trees

#27 MeSH descriptor: [Video Games] explode all trees

#28 MeSH descriptor: [Social Media] explode all trees

#29 {OR #1-#28}

#30 ((quit* OR stop* OR give* OR ceas* OR cess*) NEAR/2 (smok* OR tobacco* OR cigar* OR pipe* OR shisha* OR hookah* OR nargile* OR narghile* OR waterpipe*)):ti,ab,kw

#31 MeSH descriptor: [Smoking Cessation] explode all trees

#32 MeSH descriptor: [Tobacco Use Cessation] explode all trees

#33 MeSH descriptor: [Tobacco Use Disorder] explode all trees

#34 {OR #30-#33}

#35 #29 and #34 with Publication Year from 2004 to 2023, in Trials (Word variations have been searched)

A.10. ProQuest Theses Dissertations

(((TI,AB(quit*) OR TI,AB(stop*) OR TI,AB(give*) OR TI,AB(ceas*) OR TI,AB(cess*)) NEAR/3 (TI,AB(smok*) OR TI,AB(tobacco*) OR TI,AB(cigar*) OR TI,AB(pipe*) OR TI,AB(shisha*) OR TI,AB(hookah*) OR TI,AB(nargile*) OR TI,AB(narghile*) OR TI,AB(waterpipe*))) OR (SU("smoking cessation") OR SU("tobacco use cessation")) OR SU("Tobacco Use Disorder")) AND (((TI,AB("cell phone*") OR TI,AB("cellular phone*") OR TI,AB("mobile phone*") OR TI,AB("mobile device*") OR TI,AB("smart device*") OR TI,AB(phone-based) OR TI,AB(phonebased) OR TI,AB(smartphone*)) OR (TI,AB(app) OR TI,AB(apps)) OR (TI,AB(telecommunication) OR TI,AB(telehealth) OR TI,AB(telemedicine) OR TI,AB(tele-communication) OR TI,AB(tele-health) OR TI,AB(tele-medicine)) OR (TI,AB(ehealth) OR TI,AB("e health")) OR (TI,AB(internet) OR TI,AB(online) OR TI,AB("on line")) OR TI,AB(digital*) OR (TI,AB(computer*) OR TI,AB(laptop*)) OR (TI,AB(iPad*) OR (TI,AB(tablet) NEAR/5 (TI,AB(internet) OR TI,AB(computer) OR TI,AB(digital) OR TI,AB(mobile) OR TI,AB(internet) OR TI,AB(electronic) OR TI,AB(cellular) OR TI,AB(web) OR TI,AB(online) OR TI,AB("on line") OR TI,AB(smart*) OR TI,AB(mobile*)))) OR (TI,AB("m health") OR TI,AB(mhealth)) OR (TI,AB("u health") OR TI,AB(uhealth))) OR ((TI,AB(email) OR TI,AB("e mail") OR TI,AB("electronic mail")) OR (TI,AB("text messag*") OR TI,AB(SMS)) OR (TI,AB(chat-based) OR TI,AB(text-based)) OR (TI,AB("multimedia

messag*") OR TI,AB(MMS)) OR TI,AB("social media")) OR ((SU("cell phone") OR SU(smartphone) OR SU("text messaging")) OR (SU(microcomputers) OR SU("computers, handheld") OR SU(smartphone) OR SU("smart glasses") OR SU(minicomputers)) OR (SU(gamification) OR SU("mobile applications") OR SU("video games")) OR SU("Social Media"))) AND pd(>20031231)

A.11. dblp computer science library

Hand searches using the following terms: smoking, smoke, smoker, cigarettes, cigarette, tobacco

A.12. British Library e-theses online service (eThOS)

Hand searches using the following terms: smoking, smoke, smoker, cigarettes, cigarette, tobacco, digital intervention, smartphone, computer, mobile app

A.13. National Institute for Health Research UK Be Part of Research (formerly Clinical Trials Gateway)

Hand searches using the following terms: smoking, smoke, smoker, cigarettes, cigarette, tobacco

A.14. Reference list searches

The reference lists of the following reviews were searched:

1. Amiri, Sohrab, & Khan, Moien A. B. (2023). Digital interventions for smoking abstinence: A systematic review and meta-analysis of randomized control trials. *Journal of Addictive Diseases*, *41*(1), 4–29. https://doi.org/10.1080/10550887.2022.2058300
2. Aveyard, P., Madan, J., Chen, Y., Wang, D., Yahaya, I., Munafo, M., Bauld, L., & Welton, N. (2012). Effectiveness and cost-effectiveness of computer and other electronic aids for smoking cessation: A systematic review and network meta-analysis. *Health Technology Assessment*, *16*(38). https://doi.org/10.3310/hta16380
3. Barnett, A., Yang, I., Hay, K., Ding, H., Bowman, R., Fong, K., & Marshall, H. (2019). A meta-analysis of the effectiveness of smart phone applications to aid smoking cessation. *European Respiratory Journal*, *54*(suppl 63), OA5134. https://doi.org/10.1183/13993003.congress-2019.OA5134
4. Bock, B., Heron, K., Jennings, E., Morrow, K., Cobb, V., Magee, J., Fava, J., Deutsch, C., & Foster, R. (2013). A text message delivered smoking cessation intervention: The initial trial of TXT-2-Quit: Randomized controlled trial. *JMIR mHealth and uHealth*, *1*(2). https://dx.doi.org/10.2196/mhealth.2522
5. Boland, V. C., Stockings, E. A., Mattick, R. P., McRobbie, H., Brown, J., & Courtney, R. J. (2018). The Methodological Quality and Effectiveness of Technology-Based Smoking Cessation Interventions for Disadvantaged Groups: A Systematic Review and Meta-analysis. *Nicotine & Tobacco Research*, *20*(3), 276–285. https://doi.org/10.1093/ntr/ntw391
6. Brown, J. (2013). A Review of the Evidence on Technology-Based Interventions for the Treatment of Tobacco Dependence in College Health. *Worldviews on Evidence-Based Nursing*, *10*(3), 150–162. https://doi.org/10.1111/wvn.12000
7. Calvaresi, D., Calbimonte, J.-P., Dubosson, F., Najjar, A., & Schumacher, M. (2019). Social Network Chatbots for Smoking Cessation: Agent and Multi-Agent Frameworks. *IEEE/WIC/ACM International Conference on Web Intelligence*, 286–292. https://doi.org/10.1145/3350546.3352532
8. Cha, H. J., Cho, Y. S., Kim, K. J., Oh, E. J., Park, J. M., Choi, J. K., Kweon, H. J., & Cho, D. Y. (2019). Mobile Text Messaging Interventions for Smoking Cessation: A Systematic Review. *Korean Journal of Family Practice*, *9*(4), 394–400. https://doi.org/10.21215/kjfp.2019.9.4.394
9. Chebli, J.-L., Blaszczynski, A., & Gainsbury, S. M. (2016). Internet-Based Interventions for Addictive Behaviours: A Systematic Review. *Journal of Gambling Studies*, *32*(4), 1279–1304. https://doi.org/10.1007/s10899-016-9599-5

10. Chu, K.-H., Matheny, S. J., Escobar-Viera, C. G., Wessel, C., Notier, A. E., & Davis, E. M. (2021). Smartphone health apps for tobacco cessation: A systematic review. *Addictive Behaviors*, *112*, 106616. https://doi.org/10.1016/j.addbeh.2020.106616
11. Civljak, M., Stead, L. F., Hartmann-Boyce, J., Sheikh, A., & Car, J. (2013). Internet-based interventions for smoking cessation. *Cochrane Database of Systematic Reviews*, *7*. Cochrane Library. https://doi.org/10.1002/14651858.CD007078.pub4
12. Cobos-Campos, R., Sáez de Lafuente, A., Apiñaniz, A., Parraza, N., Pérez Llanos, I., & Orive, G. (2020). Effectiveness of mobile applications to quit smoking: Systematic review and meta-analysis. *Tobacco Prevention and Cessation*, *6*(November), 1–11. https://doi.org/10.18332/tpc/127770
13. Crocamo, C., Carretta, D., Ferri, M., Dias, S., Bartoli, F., & Carrá, G. (2018). Web- and text-based interventions for smoking cessation: Meta-analysis and meta-regression. *Drugs: Education, Prevention and Policy*, *25*(3), 207–216. https://doi.org/10.1080/09687637.2017.1285867
14. Danielsson, A.-K., Eriksson, A.-K., & Allebeck, P. (2014). Technology-based support via telephone or web: A systematic review of the effects on smoking, alcohol use and gambling. *Addictive Behaviors*, *39*(12), 1846–1868. https://doi.org/10.1016/j.addbeh.2014.06.007
15. Derksen, M. E., Strijp, S. van, Kunst, A. E., Daams, J. G., Jaspers, M. W. M., & Fransen, M. P. (2020). Serious games for smoking prevention and cessation: A systematic review of game elements and game effects. *J. Am. Medical Informatics Assoc.*, *27*(5), 818–833. https://doi.org/10.1093/jamia/ocaa013
16. Do, H. P., Tran, B. X., Le Pham, Q., Nguyen, L. H., Tran, T. T., Latkin, C. A., Dunne, M. P., & Baker, P. R. (2018). Which eHealth interventions are most effective for smoking cessation? A systematic review. *Patient Preference and Adherence*, *12*(null), 2065–2084. https://doi.org/10.2147/PPA.S169397
17. Fjeldsoe, B. S., Marshall, A. L., & Miller, Y. D. (2009). Behavior change interventions delivered by mobile telephone short-message service. *American Journal of Preventive Medicine*, *36*(2), 165–173. https://doi.org/10.1016/j.amepre.2008.09.040
18. Graham, A. L., Carpenter, K. M., Cha, S., Cole, S., Jacobs, M. A., Raskob, M., & Cole-Lewis, H. (2016). Systematic review and meta-analysis of Internet interventions for smoking cessation among adults. *Substance Abuse and Rehabilitation*, *7*(null), 55–69. https://doi.org/10.2147/SAR.S101660
19. Griffiths, S. E., Parsons, J., Naughton, F., Fulton, E. A., Tombor, I., & Brown, K. E. (2018). Are digital interventions for smoking cessation in pregnancy effective? A systematic review and meta-analysis. *Health Psychology Review*, *12*(4), 333–356. https://doi.org/10.1080/17437199.2018.1488602
20. Hartmann-Boyce, J., Livingstone-Banks, J., Ordóñez-Mena, J., Fanshawe, T., Lindson, N., Freeman, S., Sutton, A., Theodoulou, A., & Aveyard, P. (2021). Behavioural interventions for smoking cessation: An overview and network meta-analysis. *Cochrane Database of Systematic Reviews*, *1*. https://doi.org/10.1002/14651858.CD013229.pub2
21. Haskins, B. L., Lesperance, D., Gibbons, P., & Boudreaux, E. D. (2017). A systematic review of smartphone applications for smoking cessation. *Translational Behavioral Medicine*, *7*(2), 292–299. https://doi.org/10.1007/s13142-017-0492-2
22. He, L., Balaji, D., Wiers, R. W., Antheunis, M. L., & Krahmer, E. (2023). Effectiveness and Acceptability of Conversational Agents for Smoking Cessation: A Systematic Review and Meta-analysis. *Nicotine & Tobacco Research*, *25*(7), 1241–1250. https://doi.org/10.1093/ntr/ntac281
23. Hutton, H. E., Wilson, L. M., Apelberg, B. J., Avila Tang, E., Odelola, O., Bass, E. B., & Chander, G. (2011). A Systematic Review of Randomized Controlled Trials: Web-Based Interventions for Smoking Cessation Among Adolescents, College Students, and Adults. *Nicotine & Tobacco Research*, *13*(4), 227–238. https://doi.org/10.1093/ntr/ntq252

24. Kant, R., Yadav, P., & Bairwa, M. (2021). Effectiveness of the internet-based versus face-to-face interaction on reduction of tobacco use among adults: A meta-analysis. *Cureus*, *13*(11). https://doi.org/10.7759/cureus.19380
25. Kee, L., Anderson, C., Perez, D., & Freeman, B. (2019). The Development and Evaluation of Online Smoking Cessation Services: A Narrative Literature Review. *Journal of Smoking Cessation*, *14*(1), 12–20. Cambridge Core. https://doi.org/10.1017/jsc.2018.8
26. Kingkaew, P. (2018). *Optimising the development of effective mobile health behaviour change interventions: Text messages to support smoking cessation in Thailand* [PhD Thesis, University of Leeds]. https://etheses.whiterose.ac.uk/22922/
27. Kong, G., Ells, D. M., Camenga, D. R., & Krishnan-Sarin, S. (2014). Text messaging-based smoking cessation intervention: A narrative review. *Addictive Behaviors*, *39*(5), 907–917. https://doi.org/10.1016/j.addbeh.2013.11.024
28. Krishnan, N., Gu, J., & Abroms, L. C. (2021). Mobile phone-based messaging for tobacco cessation in low and middle-income countries: A systematic review. *Addictive Behaviors*, *113*, 106676. https://doi.org/10.1016/j.addbeh.2020.106676
29. Liu, L., Zhao, Y., Li, J., Zhang, N., Lan, Z., & Liu, X. (2023). Efficacy of digital therapeutics in smoking cessation: A systematic review and meta-analysis. *Medicine in Novel Technology and Devices*, *17*, 100209. https://doi.org/10.1016/j.medntd.2023.100209
30. Luo, T., Li, M., Williams, D., Phillippi, S., Yu, Q., Kantrow, S., Kao, Y.-H., Celestin, M., Lin, W., & Tseng, T.-S. (2021). Using social media for smoking cessation interventions: A systematic review. *Perspectives in Public Health*, *141*(1), 50–63. https://doi.org/10.1177/1757913920906845
31. McCrabb, S., Baker, A. L., Attia, J., Skelton, E., Twyman, L., Palazzi, K., McCarter, K., Ku, D., & Bonevski, B. (2019). Internet-based programs incorporating behavior change techniques are associated with increased smoking cessation in the general population: A systematic review and meta-analysis. *Annals of Behavioral Medicine*, *53*(2), 180–195. https://doi.org/10.1093/abm/kay026
32. Naslund, J. A., Kim, S. J., Aschbrenner, K. A., McCulloch, L. J., Brunette, M. F., Dallery, J., Bartels, S. J., & Marsch, L. A. (2017). Systematic review of social media interventions for smoking cessation. *Addictive Behaviors*, *73*, 81–93. https://doi.org/10.1016/j.addbeh.2017.05.002
33. Ortis, A., Caponnetto, P., Polosa, R., Urso, S., & Battiato, S. (2020). A report on smoking detection and quitting technologies. *International Journal of Environmental Research and Public Health*, *17*(7), 2614. https://doi.org/10.3390/ijerph17072614
34. Regmi, K., Kassim, N., Ahmad, N., & Tuah, N. A. A. (2017). Effectiveness of Mobile Apps for Smoking Cessation: A Review. *Tobacco Prevention and Cessation*, *3*(April). https://doi.org/10.18332/tpc/70088
35. Rooke, S., Thorsteinsson, E., Karpin, A., Copeland, J., & Allsop, D. (2010). Computer-delivered interventions for alcohol and tobacco use: A meta-analysis. *Addiction*, *105*(8), 1381–1390. https://doi.org/10.1111/j.1360-0443.2010.02975.x
36. Sampson, A., Bhochhibhoya, A., Digeralamo, D., & Branscum, P. (2015). The Use of Text Messaging for Smoking Cessation and Relapse Prevention: A Systematic Review of Evidence. *Journal of Smoking Cessation*, *10*(1), 50–58. Cambridge Core. https://doi.org/10.1017/jsc.2013.36
37. Scott-Sheldon, L. A. J., Lantini, R., Jennings, E. G., Thind, H., Rosen, R. K., Salmoirago-Blotcher, E., & Bock, B. C. (2016). Text Messaging-Based Interventions for Smoking Cessation: A Systematic Review and Meta-Analysis. *JMIR mHealth uHealth*, *4*(2), e49. https://doi.org/10.2196/mhealth.5436
38. Shah, A., Chaiton, M., Baliunas, D., & Schwartz, R. (2020). Tailored Web-Based Smoking Interventions and Reduced Attrition: Systematic Review and Meta-Analysis. *Journal of Medical Internet Research*, *22*(10), e16255. https://doi.org/10.2196/16255

39. Spohr, S. A., Nandy, R., Gandhiraj, D., Vemulapalli, A., Anne, S., & Walters, S. T. (2015). Efficacy of SMS Text Message Interventions for Smoking Cessation: A Meta-Analysis. *Journal of Substance Abuse Treatment*, *56*, 1–10. https://doi.org/10.1016/j.jsat.2015.01.011
40. Staiger, P. K., O'Donnell, R., Liknaitzky, P., Bush, R., & Milward, J. (2020). Mobile Apps to Reduce Tobacco, Alcohol, and Illicit Drug Use: Systematic Review of the First Decade. *Journal of Medical Internet Research*, *22*(11), e17156. https://doi.org/10.2196/17156
41. Taylor, G., Dalili, M., Semwal, M., Civljak, M., Sheikh, A., & Car, J. (2017). Internet-based interventions for smoking cessation. *Cochrane Database of Systematic Reviews*, *9*. https://doi.org/10.1002/14651858.CD007078.pub5
42. Thrul, J., Tormohlen, K. N., & Meacham, M. C. (2019). Social media for tobacco smoking cessation intervention: A review of the literature. *Current Addiction Reports*, *6*, 126–138. https://doi.org/10.1007/s40429-019-00246-2
43. Uthman, O. A., Nduka, C. U., Abba, M., Enriquez, R., Nordenstedt, H., Nalugoda, F., Kengne, A. P., & Ekström, A. M. (2019). Comparison of mHealth and Face-to-Face Interventions for Smoking Cessation Among People Living With HIV: Meta-Analysis. *JMIR Mhealth Uhealth*, *7*(1), e203. https://doi.org/10.2196/mhealth.9329
44. Vilardaga, R., Casellas-Pujol, E., McClernon, J. F., & Garrison, K. A. (2019). Mobile applications for the treatment of tobacco use and dependence. *Current Addiction Reports*, *6*, 86–97. https://doi.org/10.1007/s40429-019-00248-0
45. Whitaker, R., McRobbie, H., Bullen, C., Rodgers, A., Gu, Y., & Dobson, R. (2019). Mobile phone text messaging and app-based interventions for smoking cessation. *Cochrane Database of Systematic Reviews*, *10*. https://doi.org/10.1002/14651858.CD006611.pub5

~~A.~~ Ybarra, M. L., Jiang, Y., Free, C., Abroms, L. C., & Whittaker, R. (2016). Participant-level meta-analysis of mobile phone-based interventions for smoking cessation across different countries. *Preventive Medicine*, *89*, 90–97. https://doi.org/10.1016/j.ypmed.2016.05.002

~~A.~~B. BCTs included in the moderator analysis

Domain 1: Goals and planning

1.1 Goal setting (behaviour)

1.2 Problem solving

1.4 Action planning

1.5 Review behaviour goal(s)

1.6 Discrepancy between current behaviour and goal

1.7 Review outcome goal(s)

1.8 Behavioural contract

1.9 Commitment

Domain 2: Feedback and monitoring

2.2 Feedback on behaviour

2.3 Self-monitoring of behaviour

2.4 Self-monitoring of outcome(s) of behaviour

2.7 Feedback on outcome(s) of behaviour

Domain 3: Social support

3.1 Social support (unspecified)

3.3 Social support (emotional)

Domain 4: Shaping knowledge

4.1 Instruction on how to perform the behaviour

4.2 Information about antecedents

4.3 Re-attribution

4.4 Behavioural experiments

Domain 5: Natural consequences

5.1 Information about health consequences

5.2 Salience of consequences

Formatted: Underline

Formatted: Underline

Formatted: Underline

Formatted: Underline

Formatted: Underline

5.3 Information about social and environmental consequences

5.4 Monitoring of emotional consequences

5.5 Anticipated regret

5.6 Information about emotional consequences

Domain 6: Comparison of behaviour

6.2 Social comparison

6.3 Information about others' approval

Domain 7: Associations

7.1 Prompts cues

7.7 Exposure

7.8 Associative learning

Domain 8: Repetition and substitution

8.1 Behavioural practice/rehearsal

8.2 Behaviour substitution

8.4 Habit reversal

8.7 Graded tasks

Domain 9: Comparison of outcomes

9.1 Credible source

9.2 Pros and cons

9.3 Comparative imagining of future outcomes

Domain 10: Reward and threat

10.1 Material incentive (behaviour)

10.2 Material reward (behaviour)

10.4 Social reward

10.7 Self-incentive

10.9 Self-reward

Domain 11: Regulation

11.1 Pharmacological support

11.2 Reduce negative emotions

11.3 Conserving mental resources

Domain 12: Antecedents

12.1 Restructuring the physical environment

12.2 Restructuring the social environment

12.3 Avoidance/reducing exposure to cues for the behaviour

12.4 Distraction

12.5 Adding objects to the environment

12.6 Body changes

Domain 13: Identity

13.1 Identification of self as role model

13.2 Framing/reframing

13.4 Valued self-identify

13.5 Identity associated with changed behaviour

Domain 14: Scheduled consequences

14.3 Remove reward

14.4 Reward approximation

14.9 Reduce reward frequency

Domain 15: Self-belief

15.1 Verbal persuasion about capability

15.2 Mental rehearsal of successful performance

15.3 Focus on past success

15.4 Self-talk

Domain 16: Covert learning

16.1 Imaginary punishment

16.2 Imaginary reward

~~B.~~C. Studies eligible for inclusion in systematic review

Studies in bold and with an asterisk at the beginning are included in the primary and extended analyses, studies in bold without an asterisk are included in the extended analysis only. The remaining studies were not included because the authors did not provide sufficient data or materials.

Abroms, L. C., Boal, A. L., Simmens, S. J., Mendel, J. A., & Windsor, R. A. (2014). A randomized trial of Text2Quit: A text messaging program for smoking cessation. *American Journal of Preventive Medicine*, *47*(3), 242–250. https://dx.doi.org/10.1016/j.amepre.2014.04.010

Abroms, L. C., Chiang, S., Macherelli, L., Leavitt, L., & Montgomery, M. (2017). Assessing the National Cancer Institute's SmokefreeMOM text-messaging program for pregnant smokers: Pilot randomized trial. *Journal of Medical Internet Research*, *19*(10), e333. https://dx.doi.org/10.2196/jmir.8411

Abroms, L. C., Johnson, P. R., Leavitt, L. E., Cleary, S. D., Bushar, J., Brandon, T. H., & Chiang, S. C. (2017). A randomized trial of text messaging for smoking cessation in pregnant women. *American Journal of Preventive Medicine*, *53*(6), 781–790. https://doi.org/10.1016/j.amepre.2017.08.002

Abroms, L. C., Wu, K.-C., Krishnan, N., Long, M., Belay, S., Sherman, S., & McCarthy, M. (2021). A pilot randomized controlled trial of text messaging to increase tobacco treatment reach in the emergency department. *Nicotine & Tobacco Research*, *23*(9), 1597–1601. https://doi.org/10.1093/ntr/ntab036

An, L. C., Betzner, A., Schillo, B., Luxenberg, M. G., Christenson, M., Wendling, A., Saul, J. E., & Kavanaugh, A. (2010). The comparative effectiveness of clinic, work-site, phone, and Web-based tobacco treatment programs. *Nicotine & Tobacco Research*, *12*(10), 989–996. https://doi.org/10.1093/ntr/ntq133

An, L. C., Demers, M. R. S., Kirch, M. A., Considine-Dunn, S., Nair, V., Dasgupta, K., Narisetty, N., Resnicow, K., & Ahluwalia, J. (2013). A randomized trial of an avatar-hosted multiple behavior change intervention for young adult smokers. *JNCI Monographs*, *2013*(47), 209–215. https://dx.doi.org/10.1093/jncimonographs/lgt021

An, L. C., Klatt, C., Perry, C. L., Lein, E. B., Hennrikus, D. J., Pallonen, U. E., Bliss, R. L., Lando, H. A., Farley, D. M., Ahluwalia, J. S., & Ehlinger, E. P. (2008). The RealU online cessation intervention for college smokers: A randomized controlled trial. *Preventive Medicine*, *47*(2), 194–199. https://dx.doi.org/10.1016/j.ypmed.2008.04.011

Baggett, T. P., McGlave, C., Kruse, G. R., Yaqubi, A., Chang, Y., & Rigotti, N. A. (2019). SmokefreeTXT for homeless smokers: Pilot randomized controlled trial. *JMIR mHealth and uHealth*, *7*(6). https://dx.doi.org/10.2196/13162

Baskerville, N. B., Azagba, S., Norman, C., McKeown, K., & Brown, K. S. (2015). Effect of a digital social media campaign on young adult smoking cessation. *Nicotine & Tobacco Research*, *18*(3), 351–360. https://dx.doi.org/10.1093/ntr/ntv119

***Baskerville, N. B., Struik, L. L., Guindon, G. E., Norman, C. D., Whittaker, R., Burns, C., Hammond, D., Dash, D., & Brown, K. S. (2018). Effect of a mobile phone intervention on quitting smoking in a young adult population of smokers: Randomized controlled trial. *JMIR mHealth and uHealth*, *6*(10). https://dx.doi.org/10.2196/10893**

Battaglia, C., Peterson, J., Whitfield, E., Min, S.-J., Benson, S. L., Maddox, T. M., & Prochazka, A. V. (2016). Integrating motivational interviewing into a home telehealth program for veterans with posttraumatic stress disorder who smoke: A randomized controlled trial. *Journal of Clinical Psychology*, *72*(3), 194–206. https://doi.org/10.1002/jclp.22252

Beckham, J. C. (2019). ***Abstinence reinforcement therapy (ART) for homeless veteran smokers*** **(NCT02245308). https://clinicaltrials.gov/study/NCT02245308**

Begh, R., Munafò, M. R., Shiffman, S., Ferguson, S. G., Nichols, L., Mohammed, M. A., Holder, R. L., Sutton, S., & Aveyard, P. (2015). Lack of attentional retraining effects in cigarette smokers attempting cessation: A proof of concept double-blind randomised controlled trial.** ***Drug and Alcohol Dependence**,** ***149*****, 158–165. https://dx.doi.org/10.1016/j.drugalcdep.2015.01.041**

Bernstein, S. L., Dziura, J., Weiss, J., Brooks, A. H., Miller, T., Vickerman, K. A., Grau, L. E., Pantalon, M. V., Abroms, L., Collins, L. M., & Toll, B. (2023). Successful optimization of tobacco dependence treatment in the emergency department: A randomized controlled trial using the multiphase optimization strategy. *Annals of Emergency Medicine*, *81*(2), 209–221. https://dx.doi.org/10.1016/j.annemergmed.2022.08.018

BinDhim, N. F., Kevin McGeechan, & Lyndal Trevena. (2018). Smartphone Smoking Cessation Application (SSC App) trial: A multicountry double-blind automated randomised controlled trial of a smoking cessation decision-aid 'app'. *BMJ Open*, *8*(1), e017105. https://dx.doi.org/10.1136/bmjopen-2017-017105

Bloom, E. L., Japuntich, S. J., Pierro, A., Dallery, J., Leahey, T. M., & Rosen, J. (2022). Pilot trial of QuitBet: A digital social game that pays you to stop smoking. *Experimental and Clinical Psychopharmacology*, *30*(5). https://dx.doi.org/10.1037/pha0000487

Bock, B., Heron, K., Jennings, E., Morrow, K., Cobb, V., Magee, J., Fava, J., Deutsch, C., & Foster, R. (2013). A text message delivered smoking cessation intervention: The initial trial of TXT-2-Quit: Randomized controlled trial. *JMIR mHealth and uHealth*, *1*(2). https://dx.doi.org/10.2196/mhealth.2522

Borland, R., Balmford, J., & Benda, P. (2013). Population-level effects of automated smoking cessation help programs: A randomized controlled trial. *Addiction*, *108*(3), 618–628. https://doi.org/10.1111/j.1360-0443.2012.04091.x

Borland, R., Balmford, J., & Swift, E. (2015). Effects of encouraging rapid implementation and/or structured planning of quit attempts on smoking cessation outcomes: A randomized controlled trial. *Annals of Behavioral Medicine*, *49*(5), 732–742. https://dx.doi.org/10.1007/s12160-015-9706-3

Bos, J., Staiger, P. K., Hayden, M. J., Hughes, L. K., Youssef, G., & Lawrence, N. S. (2019). A randomized controlled trial of inhibitory control training for smoking cessation and reduction. *Journal of Consulting and Clinical Psychology*, *87*(9), 831–843. https://dx.doi.org/10.1037/ccp0000424

Boudreaux, E. D., Abar, B., Haskins, B., Bauman, B., & Grissom, G. (2015). Health evaluation and referral assistant: A randomized controlled trial to improve smoking cessation among emergency department patients. *Addiction Science & Clinical Practice*, *10*(1), 24. https://doi.org/10.1186/s13722-015-0045-2

Bramley, D., Riddell, T., Whittaker, R., Corbett, T., Lin, R.-B., Wills, M., Jones, M., & Rodgers, A. (2005). Smoking cessation using mobile phone text messaging is as effective in Maori as non-Maori. *The New Zealand Medical Journal*, *118*(1216). https://pubmed.ncbi.nlm.nih.gov/15937529/

Bricker, J. B., Mull, K. E., Kientz, J. A., Vilardaga, R., Mercer, L. D., Akioka, K. J., & Heffner, J. L. (2014). Randomized, controlled pilot trial of a smartphone app for smoking cessation using acceptance and commitment therapy. *Drug and Alcohol Dependence*, *143*, 87–94. https://dx.doi.org/10.1016/j.drugalcdep.2014.07.006

Bricker, J. B., Mull, K. E., McClure, J. B., Watson, N. L., & Heffner, J. L. (2018). Improving quit rates of web-delivered interventions for smoking cessation: Full-scale randomized trial of WebQuit.org versus Smokefree.gov. *Addiction*, *113*(5). https://dx.doi.org/10.1111/add.14127

Bricker, J. B., Mull, K. E., Santiago-Torres, M., Miao, Z., Perski, O., & Di, C. (2022). Smoking cessation smartphone app use over time: Predicting 12-month cessation outcomes in a 2-arm randomized trial. *Journal of Medical Internet Research*, *24*(8), e39208. https://dx.doi.org/10.2196/39208

Bricker, J. B., Watson, N. L., Heffner, J. L., Sullivan, B., Mull, K., Kwon, D., Westmaas, J. L., & Ostroff, J. (2020). A smartphone app designed to help cancer patients stop smoking: Results from a pilot randomized trial on feasibility, acceptability, and effectiveness. *JMIR Formative Research*, *4*(1). https://dx.doi.org/10.2196/16652

Bricker, J., Wyszynski, C., Comstock, B., & Heffner, J. L. (2013). Pilot randomized controlled trial of web-based acceptance and commitment therapy for smoking cessation. *Nicotine & Tobacco Research*, *15*(10). https://dx.doi.org/10.1093/ntr/ntt056

***Brown, J., Michie, S., Geraghty, A. W., Yardley, L., Gardner, B., Shahab, L., Stapleton, J. A., & West, R. (2014). Internet-based intervention for smoking cessation (StopAdvisor) in people with low and high socioeconomic status: A randomised controlled trial. *The Lancet Respiratory Medicine*, *2*(12), 997–1006. https://dx.doi.org/10.1016/S2213-2600%2814%2970195-X**

Brown, J., Michie, S., Walmsley, M., & West, R. (2016). An online documentary film to motivate quit attempts among smokers in the general population (4Weeks2Freedom): A randomized controlled trial. *Nicotine & Tobacco Research*, *18*(5), 1093–1100. https://dx.doi.org/10.1093/ntr/ntv161

Brunette, M. F., Ferron, J. C., McGurk, S. R., Williams, J. M., Harrington, A., Devitt, T., & Xie, H. (2020). Brief, web-based interventions to motivate smokers with schizophrenia: Randomized controlled trial. *JMIR Mental Health*, *7*(2), e16524. https://dx.doi.org/10.2196/16524

Brunette, M. F., Ferron, J. C., Robinson, D., Coletti, D., Geiger, P., Devitt, T., Klodnick, V., Gottlieb, J., Xie, H., Greene, M. A., Ziedonis, D., Drake, R. E., & McHugo, G. J. (2018). Brief web-based interventions for young adult smokers with severe mental illnesses: A randomized, controlled pilot study. *Nicotine & Tobacco Research*, *20*(10), 1206–1214. https://doi.org/10.1093/ntr/ntx190

Buller, D. B., Borland, R., Bettinghaus, E. P., Shane, J. H., & Zimmerman, D. E. (2014). Randomized trial of a smartphone mobile application compared to text messaging to support smoking cessation. *Telemedicine and E-Health*, *20*(3), 206–214. https://doi.org/10.1089/tmj.2013.0169

Burford, O., Jiwa, M., Carter, O., Parsons, R., & Hendrie, D. (2013). Internet-based photoaging within Australian pharmacies to promote smoking cessation: Randomized controlled trial. *Journal of Medical Internet Research*, *15*(3), e2337. https://dx.doi.org/10.2196/jmir.2337

Calhoun, P. S. (2019). *Abstinence reinforcement therapy (ART) for rural veteran smokers* (NCT01723163). https://clinicaltrials.gov/study/NCT01723163

Calhoun, P. S., Datta, S., Olsen, M., Smith, V. A., Moore, S. D., Hair, L. P., Dedert, E. A., Kirby, A., Dennis, M., Beckham, J. C., & Bastian, L. A. (2016). Comparative effectiveness of an internet-based

smoking cessation intervention versus clinic-based specialty care for veterans. *Journal of Substance Abuse Treatment*, *69*, 19–27. https://dx.doi.org/10.1016/j.jsat.2016.06.004

Camenga, M., Deepa R., Bernstein, M., Steven L., Dziura, P., James, Fiellin, M., Lynn, & Krishnan-Sarin, P., Suchitra. (2021). Feasibility of text messaging to augment brief advice and nicotine replacement therapy for smoking cessation in college students. *Journal of American College Health*, *69*(1), 1–8. https://doi.org/10.1080/07448481.2019.1643730

***Coleman, T., Clark, M., Welch, C., Whitemore, R., Leonardi-Bee, J., Cooper, S., Hewitt, C., Jones, M., Sutton, S., Watson, J., Daykin, K., Ussher, M., Parrott, S., & Naughton, F. (2022). Effectiveness of offering tailored text message, self-help smoking cessation support to pregnant women who want information on stopping smoking: MiQuit3 randomised controlled trial and meta-analysis. *Addiction*, *117*(4), 1079–1094. https://doi.org/10.1111/add.15715**

***Crane, D., Ubhi, H. K., Brown, J., & West, R. (2019). Relative effectiveness of a full versus reduced version of the 'Smoke Free' mobile application for smoking cessation: An exploratory randomised controlled trial [version 2; peer review: 2 approved]. *F1000Research*, *7*(1542). https://doi.org/10.12688/f1000research.16148.2**

Dahne, J., Player, M., Carpenter, M. J., Ford, D. W., & Diaz, V. A. (2021). Evaluation of a proactive smoking cessation electronic visit to extend the reach of evidence-based cessation treatment via primary care. *Telemedicine and E-Health*, *27*(3). https://dx.doi.org/10.1089/tmj.2020.0167

Dahne, J., Player, M. S., Strange, C., Carpenter, M. J., Ford, D. W., King, K., Miller, S., Kruis, R., Hawes, E., & Hidalgo, J. E. (2022). Proactive electronic visits for smoking cessation and chronic obstructive pulmonary disease screening in primary care: Randomized controlled trial of feasibility, acceptability, and efficacy. *Journal of Medical Internet Research*, *24*(8), e38663. https://dx.doi.org/10.2196/38663

***Dallery, J., Raiff, B. R., Kim, S. J., Marsch, L. A., Stitzer, M., & Grabinski, M. J. (2017). Nationwide access to an internet-based contingency management intervention to promote smoking cessation: A randomized controlled trial. *Addiction*, *112*(5), 875–883. https://dx.doi.org/10.1111/add.13715**

Danaher, B. G., Tyler, M. S., Crowley, R. C., Brendryen, H., & Seeley, J. R. (2019). Outcomes and device usage for fully automated internet interventions designed for a smartphone or personal computer: The MobileQuit smoking cessation randomized controlled trial. *Journal of Medical Internet Research*, *21*(6), e13290. https://dx.doi.org/10.2196/13290

DeZee, K. J., Wink, J. S., & Cowan, C. M. (2013). Internet versus in-person counseling for patients taking varenicline for smoking cessation. *Military Medicine*, *178*(4), 401–405. https://doi.org/10.7205/MILMED-D-12-00272

***Dingle, G. A., & Carter, N. A. (2017). Smoke into Sound: A pilot randomised controlled trial of a music cravings management program for chronic smokers attempting to quit. *Musicae Scientiae*, *21*(2), 151–177. https://dx.doi.org/10.1177/1029864916682822**

Emmons, K. M., Puleo, E., Sprunck-Harrild, K., Ford, J., Ostroff, J. S., Hodgson, D., Greenberg, M., Diller, L., de Moor, J., & Tyc, V. (2013). Partnership for health-2, a web-based versus print smoking cessation intervention for childhood and young adult cancer survivors: Randomized comparative effectiveness study. *Journal of Medical Internet Research*, *15*(11), e218. https://dx.doi.org/10.2196/jmir.2533

Faro, J. M., Chen, J., Flahive, J., Nagawa, C. S., Orvek, E. A., Houston, T. K., Allison, J. J., Person, S. D., Smith, B. M., & Blok, A. C. (2023). Effect of a machine learning recommender system and viral peer marketing intervention on smoking cessation: A randomized clinical trial. *JAMA Network Open*, *6*(1), e2250665–e2250665. https://dx.doi.org/10.1001/jamanetworkopen.2022.50665

Ferguson, S. (2015). *Cognitive and behavioural support for cigarette smokers trying to quit* (ACTRN12611000325909). https://anzctr.org.au/Trial/Registration/TrialReview.aspx?ACTRN=12611000325909

Forinash, A. B., Yancey, A., Chamness, D., Koerner, J., Inteso, C., Miller, C., Gross, G., & Mathews, K. (2018). Smoking cessation following text message intervention in pregnant women. *Annals of Pharmacotherapy*, *52*(11), 1109–1116. https://doi.org/10.1177/1060028018780448

Fraser, D., Kobinsky, K., Smith, S. S., Kramer, J., Theobald, W. E., & Baker, T. B. (2014). Five population-based interventions for smoking cessation: A MOST trial. *Translational Behavioral Medicine*, *4*(4), 382–390. https://dx.doi.org/10.1007/s13142-014-0278-8

Free, C., Knight, R., Robertson, S., Whittaker, R., Edwards, P., Zhou, W., Rodgers, A., Cairns, J., Kenward, M. G., & Roberts, I. (2011). Smoking cessation support delivered via mobile phone text messaging (txt2stop): A single-blind, randomised trial. *The Lancet*, *378*(9785), 49–55. https://dx.doi.org/10.1016/S0140-6736%2811%2960701-0

Free, C., Whittaker, R., Knight, R., Abramsky, T., Rodgers, A., & Roberts, I. G. (2009). Txt2stop: A pilot randomised controlled trial of mobile phone-based smoking cessation support. *Tobacco Control*, *18*(2), 88. https://dx.doi.org/10.1136/tc.2008.026146

Gallagher, R., Chow, C. K., Parker, H., Neubeck, L., Celermajer, D. S., Redfern, J., Tofler, G., Buckley, T., Schumacher, T., Hyun, K., Boroumand, F., & Figtree, G. (2023). The effect of a game-based mobile app 'MyHeartMate' to promote lifestyle change in coronary disease patients: A randomized controlled trial. *European Heart Journal - Digital Health*, *4*(1), 33–42. https://doi.org/10.1093/ehjdh/ztac069

Garey, L., Smit, T., Neighbors, C., Gallagher, M. W., & Zvolensky, M. J. (2021). Personalized Feedback for Smoking and Anxiety Sensitivity: A Randomized Controlled Trial. *Substance Use & Misuse*, *56*(7), 929–940. https://dx.doi.org/10.1080/10826084.2021.1900255

***Garrison, K. A., Pal, P., O'Malley, S. S., Pittman, B. P., Gueorguieva, R., Rojiani, R., Scheinost, D., Dallery, J., & Brewer, J. A. (2020). Craving to Quit: A randomized controlled trial of smartphone app–based mindfulness training for smoking cessation. *Nicotine & Tobacco Research*, *22*(3), 324–331. https://dx.doi.org/10.1093/ntr/nty126**

Gordon, J., Bell, M., Armin, J., Giacobbi, P., & Nair, U. (2021). A telephone-based guided imagery tobacco cessation intervention: Results of a randomized feasibility trial. *Translational Behavioral Medicine*, *11*(2). https://dx.doi.org/10.1093/tbm/ibaa052

Gore, M. O., Krantz, M. J., Albright, K., Beaty, B., Coronel-Mockler, S., Bull, S., & Estacio, R. O. (2019). A controlled trial of mobile short message service among participants in a rural cardiovascular disease prevention program. *Preventive Medicine Reports*, *13*, 126–131. https://doi.org/10.1016/j.pmedr.2018.11.021

Graham, A. L., Cobb, N. K., Papandonatos, G. D., Moreno, J. L., Kang, H., Tinkelman, D. G., Bock, B. C., Niaura, R. S., & Abrams, D. B. (2011). A randomized trial of internet and telephone treatment for smoking cessation. *Archives of Internal Medicine*, *171*(1), 46–53. https://dx.doi.org/10.1001/archinternmed.2010.451

Graham, A. L., Papandonatos, G. D., Cha, S., Amato, M. S., Jacobs, M. A., Cohn, A. M., Abroms, L. C., & Whittaker, R. (2022). Effectiveness of an optimized text message and Internet intervention for smoking cessation: A randomized controlled trial. *Addiction*, *117*(4), 1035–1046. https://doi.org/10.1111/add.15677

Graham, A. L., Papandonatos, G. D., Cha, S., Erar, B., & Amato, M. S. (2018). Improving adherence to smoking cessation treatment: Smoking outcomes in a web-based randomized trial. *Annals of Behavioral Medicine*, *52*(4), 331–341. https://doi.org/10.1093/abm/kax023

Graham, A. L., Papandonatos, G. D., Cobb, C. O., Cobb, N. K., Niaura, R. S., Abrams, D. B., & Tinkelman, D. G. (2015). Internet and telephone treatment for smoking cessation: Mediators and moderators of short-term abstinence. *Nicotine & Tobacco Research*, *17*(3), 299–308. https://dx.doi.org/10.1093/ntr/ntu144

Gram, I. T., Larbi, D., & Wangberg, S. C. (2019). Comparing the efficacy of an identical, tailored smoking cessation intervention delivered by mobile text messaging versus email: Randomized controlled trial. *JMIR mHealth and uHealth*, *7*(9), e12137. https://doi.org/10.2196/12137

***Haaga, D. A. F., Kaufmann, A., & Malloy, E. J. (2020). Looming vulnerability and smoking cessation attempts. *Nicotine & Tobacco Research*, *22*(9), 1439–1445. https://doi.org/10.1093/ntr/ntaa034**

Harrington, K. F., Kim, Y., Chen, M., Ramachandran, R., Pisu, M., Sadasivam, R. S., Houston, T. K., & Bailey, W. C. (2016). Web-based intervention for transitioning smokers from inpatient to outpatient care: An RCT. *American Journal of Preventive Medicine*, *51*(4), 620–629. https://dx.doi.org/10.1016/j.amepre.2016.04.008

Hébert, E. T., Ra, C. K., Alexander, A. C., Helt, A., Moisiuc, R., Kendzor, D. E., Vidrine, D. J., Funk-Lawler, R. K., & Businelle, M. S. (2020). A mobile just-in-time adaptive intervention for smoking cessation: Pilot randomized controlled trial. *Journal of Medical Internet Research*, *22*(3), e16907. https://dx.doi.org/10.2196/16907

Heffner, J. L., Kelly, M. M., Reilly, E. D., Reece, S. G., Claudio, T., Serfozo, E., Baker, K., Watson, N. L., & Karekla, M. (2023). An avatar-led web-based and sms text message smoking cessation program for socioeconomically disadvantaged veterans: Pilot randomized controlled trial. *JMIR Formative Research*, *7*(1), e44503. https://dx.doi.org/10.2196/44503

Heffner, J. L., Kelly, M. M., Waxmonsky, J., Mattocks, K., Serfozo, E., Bricker, J. B., Mull, K. E., Watson, N. L., & Ostacher, M. (2020). Pilot randomized controlled trial of web-delivered acceptance and commitment therapy versus Smokefree.gov for smokers with bipolar disorder. *Nicotine & Tobacco Research*, *22*(9). https://dx.doi.org/10.1093/ntr/ntz242

Heffner, J. L., Mull, K. E., Watson, N. L., McClure, J. B., Bricker, J. L. ; :, & Babor. (2020). Long-term smoking cessation outcomes for sexual minority versus nonminority smokers in a large randomized controlled trial of two web-based interventions. *Nicotine & Tobacco Research*, *22*(9), 1596–1604. https://dx.doi.org/10.1093/ntr/ntz112

***Herbeć, A. A. (2019). Use of smartphone-based interventions to support smoking cessation and pharmacotherapy use [Ph.D., University of London, University College London (United Kingdom)]. In *PQDT - UK & Ireland* (2307312480). ProQuest Dissertations & Theses Global. https://www.proquest.com/dissertations-theses/use-smartphone-based-interventions-support/docview/2307312480/se-2?accountid=14511**

***Herbeć, A. A., Brown, J., Tombor, I., Michie, S., & West, R. (2014). Pilot randomized controlled trial of an internet-based smoking cessation intervention for pregnant smokers ('MumsQuit'). *Drug and Alcohol Dependence*, *140*, 130–136. https://dx.doi.org/10.1016/j.drugalcdep.2014.04.010**

Hernández-Torres, R., Cartujano-Barrera, F., Chavez-Iñiguez, A., Ossip, D., Rivera, M. P., Castro-Figueroa, E. M., & Cupertino, A. P. (2023). Decídetexto: A mobile smoking cessation intervention among Puerto Ricans in the US. *Cancer Epidemiology, Biomarkers & Prevention*, *32*(1_Supplement), B004–B004. https://doi.org/10.1158/1538-7755.DISP22-B004

Hertzberg, J. S., Carpenter, V. L., Kirby, A. C., Calhoun, P. S., Moore, S. D., Dennis, M. F., Dennis, P. A., Dedert, E. A., & Beckham, J. C. (2013). Mobile contingency management as an adjunctive smoking cessation treatment for smokers with posttraumatic stress disorder. *Nicotine & Tobacco Research*, *15*(11), 1934–1938. https://doi.org/10.1093/ntr/ntt060

Hicks, T. A., Thomas, S. P., Wilson, S. M., Calhoun, P. S., Kuhn, E. R., & Beckham, J. C. (2017). A preliminary investigation of a relapse prevention mobile application to maintain smoking abstinence among individuals with posttraumatic stress disorder. *Journal of Dual Diagnosis*, *13*(1), 15–20. https://dx.doi.org/10.1080/15504263.2016.1267828

Hollis, J. F., Polen, M. R., Whitlock, E. P., Lichtenstein, E., Mullooly, J. P., Velicer, W. F., & Redding, C. A. (2005). Teen Reach: Outcomes from a randomized, controlled trial of a tobacco reduction program for teens seen in primary medical care. *Pediatrics*, *115*(4), 981–989. https://doi.org/10.1542/peds.2004-0981

Hors-Fraile, S., Candel, M. J. J. M., Schneider, F., Malwade, S., Nunez-Benjumea, F. J., Syed-Abdul, S., Fernandez-Luque, L., & de Vries, H. (2022). Applying collective intelligence in health recommender systems for smoking cessation: A comparison trial. *Electronics*, *11*(8). https://doi.org/10.3390/electronics11081219

Houston, T. K., Chen, J., Amante, D. J., Blok, A. C., Nagawa, C. S., Wijesundara, J. G., Kamberi, A., Allison, J. J., Person, S. D., & Flahive, J. (2022). Effect of technology-assisted brief abstinence game on long-term smoking cessation in individuals not yet ready to quit: A randomized clinical trial. *JAMA Internal Medicine*, *182*(3), 303–312. https://dx.doi.org/10.1001/jamainternmed.2021.7866

Houston, T. K., Sadasivam, R. S., Allison, J. J., Ash, A. S., Ray, M. N., English, T. M., Hogan, T. P., & Ford, D. E. (2015). Evaluating the QUIT-PRIMO clinical practice ePortal to increase smoker engagement with online cessation interventions: A national hybrid type 2 implementation study. *Implementation Science*, *10*(1), 154. https://dx.doi.org/10.1186/s13012-015-0336-8

Hughes, S. L., Seymour, R. B., Campbell, R. T., Shaw, J. W., Fabiyi, C., & Sokas, R. (2011). Comparison of two health-promotion programs for older workers. *American Journal of Public Health*, *101*(5). https://dx.doi.org/10.2105/AJPH.2010.300082

Humfleet, G. L., Hall, S. M., Delucchi, K. L., & Dilley, J. W. (2013). A randomized clinical trial of smoking cessation treatments provided in hiv clinical care settings. *Nicotine & Tobacco Research*, *15*(8), 1436–1445. https://dx.doi.org/10.1093/ntr/ntt005

Iacoviello, B. M. (2022). *Innovative digital therapeutic for smoking cessation* (NCT03694327). https://clinicaltrials.gov/study/NCT03694327

Jackson, S. E., Kale, D. E., Beard, E., Perski, O., West, R., & Brown, J. (2023). *Effectiveness of the offer of the Smoke Free smartphone application compared with no intervention for smoking

***cessation: A pragmatic randomised controlled trial*. medRxiv. https://doi.org/10.1101/2023.01.12.23284463**

Japuntich, S. J., Zehner, M. E., Smith, S. S., Jorenby, D. E., Valdez, J. A., Fiore, M. C., Baker, T. B., & Gustafson, D. H. (2006). Smoking cessation via the internet: A randomized clinical trial of an internet intervention as adjuvant treatment in a smoking cessation intervention. *Nicotine & Tobacco Research*, *8*(Suppl_1), S59–S67. https://dx.doi.org/10.1080/14622200601047900

Johnson, K. C., Thomas, F., Richey, P., Tran, Q. T., Tylavsky, F., Miro, D., & Coday, M. (2017). The primary results of the Treating Adult Smokers at Risk for Weight Gain with Interactive Technology (TARGIT) study. *Obesity*, *25*(10), 1691–1698. https://doi.org/10.1002/oby.21968

Jones, H. A., Heffner, J. L., Mercer, L., Wyszynski, C. M., Vilardaga, R., & Bricker, J. B. (2015). Web-based acceptance and commitment therapy smoking cessation treatment for smokers with depressive symptoms. *Journal of Dual Diagnosis*, *11*(1), 56–62. https://doi.org/10.1080/15504263.2014.992588

Joyce, C., Saulsgiver, K., Mohanty, S., Bachireddy, C., Molfetta, C., Steffy, M., Yoder, A., & Buttenheim, A. (2021). Remote patient monitoring and incentives to support smoking cessation among pregnant and postpartum Medicaid members: Three randomized controlled pilot studies. *JMIR Formative Research*, *5*(9). https://dx.doi.org/10.2196/27801

***Kahler, C. W., Cohn, A. M., Costantino, C., Toll, B. A., Spillane, N. S., & Graham, A. L. (2020). A digital smoking cessation program for heavy drinkers: Pilot randomized controlled trial. *JMIR Formative Research*, *4*(6), e7570. https://dx.doi.org/10.2196/formative.7570**

Keller-Hamilton, B., Stevens, E. M., Villanti, A. C., Leshner, G., Wagener, T. L., & Mays, D. (2023). Messaging to prevent and reduce young adults' waterpipe tobacco smoking: A randomized trial. *Addictive Behaviors*, *138*, 107546. https://doi.org/10.1016/j.addbeh.2022.107546

Keogan, S., Li, S., & Clancy, L. (2019). Allen Carr's Easyway to Stop Smoking-A randomised clinical trial. *Tobacco Control*, *28*(4), 414–419. https://dx.doi.org/10.1136/tobaccocontrol-2018-054243

Kierstead, E. C., Harvey, E., Sanchez, D., Horn, K., Abroms, L. C., Spielberg, F., Stanton, C. A., Debnam, C., Cohn, A. M., Gray, T., Magnus, M., Patel, M., Niaura, R., & Elf, J. L. (2021). A pilot randomized controlled trial of a tailored smoking cessation program for people living with HIV in the Washington, D.C. metropolitan area. *BMC Research Notes*, *14*(1), 2. https://doi.org/10.1186/s13104-020-05417-3

Kim, S. S., Lee, S. A., Mejia, J., Cooley, M. E., & Demarco, R. F. (2020). Pilot randomized controlled trial of a digital storytelling intervention for smoking cessation in women living with HIV. *Annals of Behavioral Medicine*, *54*(6), 447–454. https://dx.doi.org/10.1093/abm/kaz062

***King, E., Cheyne, H., Abhyankar, P., Elders, A., Grindle, M., Hapca, A., Jones, C., O'Carroll, R., Steele, M., & Williams, B. (2022). Promoting smoking cessation during pregnancy: A feasibility and pilot trial of a digital storytelling intervention delivered via text-messaging. *Patient Education and Counseling*, *105*(7), 2562–2572. https://dx.doi.org/10.1016/j.pec.2021.12.019**

Klimis, H., Marschner, S., Von Huben, A., Thiagalingam, A., & Chow, C. K. (2020). Predictors of smoking cessation in a lifestyle-focused text-message support programme delivered to people with coronary heart disease: An analysis from the Tobacco Exercise and Diet Messages (TEXTME) randomised clinical trial. *Tobacco Use Insights*, *13*(101608659). https://dx.doi.org/10.1177/1179173X20901486

Klimis, H., Thiagalingam, A., McIntyre, D., Marschner, S., Von Huben, A., & Chow, C. K. (2021). Text messages for primary prevention of cardiovascular disease: The TextMe2 randomized clinical trial. *American Heart Journal*, *242*, 33–44. https://doi.org/10.1016/j.ahj.2021.08.009

Krebs, P., Burkhalter, J., Fiske, J., Snow, H., Schofield, E., Iocolano, M., Borderud, S., & Ostroff, J. S. (2019). The QuitIT coping skills game for promoting tobacco cessation among smokers diagnosed with cancer: Pilot randomized controlled trial. *JMIR mHealth and uHealth*, *7*(1). https://dx.doi.org/10.2196/10071

Krishnan, N., Elf, J. L., Chon, S., & Golub, J. E. (2019). COach2Quit: A pilot randomized controlled trial of a personal carbon monoxide monitor for smoking cessation. *Nicotine & Tobacco Research*, *21*(11), 1573–1577. https://doi.org/10.1093/ntr/nty182

Kruse, G. R., Park, E. R., Chang, Y., Haberer, J. E., Abroms, L. C., Shahid, N. N., Howard, S., Haas, J. S., & Rigotti, N. A. (2020). Proactively offered text messages and mailed nicotine replacement therapy for smokers in primary care practices: A pilot randomized trial. *Nicotine & Tobacco Research*, *22*(9), 1509–1514. https://doi.org/10.1093/ntr/ntaa050

Kurti, A. N., Nighbor, T. D., Tang, K., Bolívar, H. A., Evemy, C. G., Skelly, J., & Higgins, S. T. (2022). Effect of smartphone-based financial incentives on peripartum smoking among pregnant individuals: A randomized clinical trial. *JAMA Network Open*, *5*(5), e2211889–e2211889. https://dx.doi.org/10.1001/jamanetworkopen.2022.11889

Kurti, A. N., Tang, K., Bolivar, H. A., Evemy, C., Medina, N., Skelly, J., Nighbor, T., & Higgins, S. T. (2020). Smartphone-based financial incentives to promote smoking cessation during pregnancy: A pilot study. *Preventive Medicine*, *140*, 106201. https://doi.org/10.1016/j.ypmed.2020.106201

Lawrence, T., Aveyard, P., Cheng, K. K., Griffin, C., Johnson, C., & Croghan, E. (2005). Does stage-based smoking cessation advice in pregnancy result in long-term quitters? 18-month postpartum follow-up of a randomized controlled trial. *Addiction*, *100*(1), 107–116. https://doi.org/10.1111/j.1360-0443.2005.00936.x

Lear, S. A., Singer, J., Banner-Lukaris, D., Horvat, D., Park, J. E., Bates, J., & Ignaszewski, A. (2014). Randomized trial of a virtual cardiac rehabilitation program delivered at a distance via the Internet. *Circulation: Cardiovascular Quality and Outcomes*, *7*(6), 952–959. https://doi.org/10.1161/CIRCOUTCOMES.114.001230

Lenert, L., Muñoz, R. F., Perez, J. E., & Bansod, A. (2004). Automated e-mail messaging as a tool for improving quit rates in an internet smoking cessation intervention. *Journal of the American Medical Informatics Association*, *11*(4), 235–240. https://doi.org/10.1197/jamia.M1464

Leykin, Y., Aguilera, A., Torres, L. D., Pérez-Stable, E. J., & Muñoz, R. F. (2012). Interpreting the outcomes of automated internet-based randomized trials: Example of an international smoking cessation study. *Journal of Medical Internet Research*, *14*(1), e5. https://dx.doi.org/10.2196/jmir.1829

Loughead, J., Falcone, M., Wileyto, E. P., Albelda, B., Audrain-McGovern, J., Cao, W., Kurtz, M. M., Gur, R. C., & Lerman, C. (2016). Can brain games help smokers quit?: Results of a randomized clinical trial. *Drug and Alcohol Dependence*, *168*, 112–118. https://dx.doi.org/10.1016/j.drugalcdep.2016.08.621

Maddison, R., Jiang, Y., Stewart, R., Scott, T., Kerr, A., Whittaker, R., Benatar, J., Rolleston, A., Estabrooks, P., & Dale, L. (2021). An intervention to improve medication adherence in people with

heart disease (Text4HeartII): Randomized controlled trial. *JMIR mHealth and uHealth*, *9*(6), e24952. https://doi.org/10.2196/24952

Marler, J. D., Fujii, C. A., Utley, M. T., Balbierz, D. J., Galanko, J. A., & Utley, D. S. (2022). Outcomes of a comprehensive mobile smoking cessation program with nicotine replacement therapy in adult smokers: Pilot randomized controlled trial. *JMIR mHealth and uHealth*, *10*(11), e41658. https://dx.doi.org/10.2196/41658

Mason, D., Gilbert, H., & Sutton, S. (2012). Effectiveness of web-based tailored smoking cessation advice reports (iQuit): A randomized trial. *Addiction*, *107*(12), 2183–2190. https://doi.org/10.1111/j.1360-0443.2012.03972.x

Mays, D., Johnson, A. C., Phan, L., Sanders, C., Shoben, A., Tercyak, K. P., Wagener, T. L., Brinkman, M. C., & Lipkus, I. M. (2021). Tailored mobile messaging intervention for waterpipe tobacco cessation in young adults: A randomized trial. *American Journal of Public Health*, *111*(9), 1686–1695. https://dx.doi.org/10.2105/AJPH.2021.306389

McClure, E. A., Campbell, A. N. C., Pavlicova, M., Hu, M., Winhusen, T., Vandrey, R. G., Ruglass, L. M., Covey, L. S., Stitzer, M. L., Kyle, T. L., & Nunes, E. V. (2015). Cigarette smoking during substance use disorder treatment: Secondary outcomes from a national drug abuse treatment clinical trials network study. *Journal of Substance Abuse Treatment*, *53*, 39–46. https://doi.org/10.1016/j.jsat.2014.12.007

McClure, J. B., Anderson, M. L., Bradley, K., An, L. C., & Catz, S. L. (2016). Evaluating an adaptive and interactive mhealth smoking cessation and medication adherence program: A randomized pilot feasibility study. *JMIR mHealth and uHealth*, *4*(3). https://dx.doi.org/10.2196/mhealth.6002

McClure, J. B., Peterson, D., Derry, H., Riggs, K., Saint-Johnson, J., Nair, V., An, L., & Shortreed, S. M. (2014). Exploring the "active ingredients" of an online smoking intervention: A randomized factorial trial. *Nicotine & Tobacco Research*, *16*(8), 1129–1139. https://dx.doi.org/10.1093/ntr/ntu057

McDonnell, D. D., Kazinets, G., Lee, H.-J., & Moskowitz, J. M. (2011). An internet-based smoking cessation program for Korean Americans: Results from a randomized controlled trial. *Nicotine & Tobacco Research*, *13*(5), 336–343. https://doi.org/10.1093/ntr/ntq260

McKay, H., Danaher, B., Seeley, J., Lichtenstein, E., & Gau, J. (2008). Comparing two web-based smoking cessation programs: Randomized controlled trial. *Journal of Medical Internet Research*, *10*(5), e993. https://dx.doi.org/10.2196/jmir.993

McNaughton, B., Frohlich, J., Graham, A., & Young, Q.-R. (2013). Extended interactive voice response telephony (IVR) for relapse prevention after smoking cessation using varenicline and IVR: A pilot study. *BMC Public Health*, *13*(1), 824. https://doi.org/10.1186/1471-2458-13-824

***McRobbie, H. J., Phillips-Waller, A., El Zerbi, C., McNeill, A., Hajek, P., Pesola, F., Balmford, J., Ferguson, S. G., Li, L., & Lewis, S. (2020). Nicotine replacement treatment, e-cigarettes and an online behavioural intervention to reduce relapse in recent ex-smokers: A multinational four-arm RCT. *Health Technology Assessment*, *24*(68), 1. https://dx.doi.org/10.3310/hta24680**

Meacham, M. C., Ramo, D. E., Prochaska, J. J., Maier, L. J., Delucchi, K. L., Kaur, M., & Satre, D. D. (2021). A Facebook intervention to address cigarette smoking and heavy episodic drinking: A pilot randomized controlled trial. *Journal of Substance Abuse Treatment*, *122*. https://doi.org/10.1016/j.jsat.2020.108211

Field Code Changed

Minami, H., Nahvi, S., Arnsten, J., Brinkman, H., Rivera-Mindt, M., Wetter, D., Bloom, E., Price, L., Richman, E., Betzler, T., Stockmal, C., Donnelly, R., McClain, L., Kennedy, K., Vieira, C., Fine, M., McCarthy, D., Thomas, J., Hecht, J., & Brown, R. (2022). A pilot randomized controlled trial of smartphone-assisted mindfulness-based intervention with contingency management for smokers with mood disorders. *Experimental and Clinical Psychopharmacology*, *30*(5). https://dx.doi.org/10.1037/pha0000506

Moskowitz, J. M., McDonnell, D. D., Kazinets, G., & Lee, H.-J. (2016). Online smoking cessation program for Korean Americans: Randomized trial to test effects of incentives for program completion and interim surveys. *Preventive Medicine*, *86*, 70–76. https://dx.doi.org/10.1016/j.ypmed.2016.01.019

Muramoto, M. L., Hopkins, A., Bell, M., Allen, A., Nair, U., & Connolly, T. E. (2021). Results of a feasibility study of helpers stay quit training for smoking relapse prevention. *Nicotine & Tobacco Research*, *23*(4), 711–715. https://doi.org/10.1093/ntr/ntaa176

***Mutter, E. R., Oettingen, G., & Gollwitzer, P. M. (2020). An online randomised controlled trial of mental contrasting with implementation intentions as a smoking behaviour change intervention. *Psychology & Health*, *35*(3), 318–345. https://dx.doi.org/10.1080/08870446.2019.1634200**

***Naughton, F., Cooper, S., Foster, K., Emery, J., Leonardi-Bee, J., Sutton, S., Jones, M., Ussher, M., Whitemore, R., & Leighton, M. (2017). Large multi-centre pilot randomized controlled trial testing a low-cost, tailored, self-help smoking cessation text message intervention for pregnant smokers (MiQuit). *Addiction*, *112*(7), 1238–1249. https://doi.org/10.1111/add.13802**

***Naughton, F., Hope, A., Siegele-Brown, C., Grant, K., Barton, G., Notley, C., Mascolo, C., Coleman, T., Shepstone, L., Sutton, S., Prevost, A. T., Crane, D., Greaves, F., & High, J. (2023). An automated, online feasibility randomized controlled trial of a just-in-time adaptive intervention for smoking cessation (Quit Sense). *Nicotine & Tobacco Research*, *25*(7), 1319–1329. https://doi.org/10.1093/ntr/ntad032**

***Naughton, F., Jamison, J., Boase, S., Sloan, M., Gilbert, H., Prevost, A. T., Mason, D., Smith, S., Brimicombe, J., Evans, R., & Sutton, S. (2014). Randomized controlled trial to assess the short-term effectiveness of tailored web- and text-based facilitation of smoking cessation in primary care (iQuit in Practice). *Addiction*, *109*(7), 1184–1193. https://doi.org/10.1111/add.12556**

Naughton, F., Prevost, A. T., Gilbert, H., & Sutton, S. (2012). Randomized controlled trial evaluation of a tailored leaflet and SMS text message self-help intervention for pregnant smokers (MiQuit). *Nicotine & Tobacco Research*, *14*(5), 569–577. https://dx.doi.org/10.1093/ntr/ntr254

Noonan, D., Silva, S., Njuru, J., Bishop, T., Fish, L. J., Simmons, L. A., Choi, S. H., & Pollak, K. I. (2018). Feasibility of a text-based smoking cessation intervention in rural older adults. *Health Education Research*, *33*(1), 81–88. https://dx.doi.org/10.1093/ntr/nty208

O'Connor, M., Whelan, R., Bricker, J., & McHugh, L. (2020). Randomized controlled trial of a smartphone application as an adjunct to acceptance and commitment therapy for smoking cessation. *Behavior Therapy*, *51*(1), 162–177. https://doi.org/10.1016/j.beth.2019.06.003

***Palmer, A. M., Carpenter, M. J., Baker, N. L., Froeliger, B., Foster, M. G., Garland, E. L., Saladin, M. E., & Toll, B. A. (2023). Development of two novel treatments to promote smoking cessation: Savor and retrieval-extinction training pilot clinical trial findings. *Experimental and Clinical Psychopharmacology*. https://doi.org/10.1037/pha0000644**

Pandian, J. D., Kate, M. P., Sylaja, P. N., Khurana, D., Pamidimukkala, V., Ray, B. K., Nambiar, V. K., Aaron, S., Mittal, G. K., Nagarjunakonda, S., Pai, A. R., Gorthi, S. P., Kumaravelu, S., Reddy, Y. M., Narayan, S., Borah, N. C., Das, R., Kulkarni, G. B., Huded, V., … George, J. (2023). Secondary prevention with a structured semi-interactive stroke prevention package in INDIA (SPRINT INDIA): A multicentre, randomised controlled trial. *The Lancet Global Health*, *11*(3), e425–e435. https://dx.doi.org/10.1016/S2214-109X(22)00544-7

Papandonatos, G. D., Erar, B., Stanton, C. A., & Graham, A. L. (2016). Online community use predicts abstinence in combined Internet/phone intervention for smoking cessation. *Journal of Consulting and Clinical Psychology*, *84*(7), 633. https://dx.doi.org/10.1037/ccp0000099

Park, E., Choi, S., & Duffy, S. (2016). The effect of re-randomization in a smoking cessation trial. *American Journal of Health Behavior*, *40*(5), 667-674(8). https://doi.org/10.5993/AJHB.40.5.14

Patten, C. A., Croghan, I. T., Meis, T. M., Decker, P. A., Pingree, S., Colligan, R. C., Dornelas, E. A., Offord, K. P., Boberg, E. W., Baumberger, R. K., Hurt, R. D., & Gustafson, D. H. (2006). Randomized clinical trial of an Internet-based versus brief office intervention for adolescent smoking cessation. *Patient Education and Counseling*, *64*(1), 249–258. https://dx.doi.org/10.1016/j.pec.2006.03.001

Patten, C. A., Koller, K. R., Sinicrope, P. S., Prochaska, J. J., Young, C., Resnicow, K., Decker, P. A., Hughes, C. A., Merritt, Z. T., McConnell, C. R., Huang, M., & Thomas, T. K. (2023). Facebook intervention to connect Alaska Native people with resources and support to quit smoking: CAN quit pilot randomized controlled trial. *Nicotine & Tobacco Research*, *25*(4), 803–813. https://doi.org/10.1093/ntr/ntac221

***Pbert, L., Druker, S., Crawford, S., Frisard, C., Trivedi, M., Osganian, S. K., & Brewer, J. (2020). Feasibility of a smartphone app with mindfulness training for adolescent smoking cessation: Craving to Quit (C2Q)-teen. *Mindfulness*, *11*(3). https://dx.doi.org/10.1007/s12671-019-01273-w**

Pechmann, C., Delucchi, K., Lakon, C. M., & Prochaska, J. J. (2017). Randomised controlled trial evaluation of Tweet2Quit: A social network quit-smoking intervention. *Tobacco Control*, *26*(2), 188. https://dx.doi.org/10.1136/tobaccocontrol-2015-052768

Pechmann, C., Pan, L., Delucchi, K., Lakon, C. M., & Prochaska, J. J. (2015). Development of a Twitter-based intervention for smoking cessation that encourages high-quality social media interactions via automessages. *Journal of Medical Internet Research*, *17*(2). https://dx.doi.org/10.2196/jmir.3772

Peek, J., Hay, K., Hughes, P., Kostellar, A., Kumar, S., Bhikoo, Z., Serginson, J., & Marshall, H. M. (2021). Feasibility and acceptability of a smoking cessation smartphone app (My QuitBuddy) in older persons: Pilot randomized controlled trial. *JMIR Formative Research*, *5*(4). https://dx.doi.org/10.2196/24976

Peiris, D., Wright, L., News, M., Rogers, K., Redfern, J., Chow, C., & Thomas, D. (2019). A smartphone app to assist smoking cessation among Aboriginal Australians: Findings from a pilot randomized controlled trial. *JMIR mHealth and uHealth*, *7*(4). https://dx.doi.org/10.2196/12745

Pfaeffli Dale, L., Whittaker, R., Jiang, Y., Stewart, R., Rolleston, A., & Maddison, R. (2015). Text message and internet support for coronary heart disease self-management: Results from the Text4Heart randomized controlled trial. *Journal of Medical Internet Research*, *17*(10), e237. https://dx.doi.org/10.2196/jmir.4944

Pike, K. J., Rabius, V., McAlister, A., & Geiger, A. (2007). American Cancer Society's QuitLink: Randomized trial of internet assistance. *Nicotine & Tobacco Research*, *9*(3), 415–420. https://doi.org/10.1080/14622200701188877

Pollak, K. I., Lyna, P., Bilheimer, A., Farrell, D., Gao, X., Swamy, G. K., & Fish, L. J. (2013). A pilot study testing SMS text delivered scheduled gradual reduction to pregnant smokers. *Nicotine & Tobacco Research*, *15*(10), 1773–1776. https://doi.org/10.1093/ntr/ntt045

Pollak, K. I., Lyna, P., Gao, X., Noonan, D., Bejarano Hernandez, S., Subudhi, S., Swamy, G. K., & Fish, L. J. (2020). Efficacy of a texting program to promote cessation among pregnant smokers: A randomized control trial. *Nicotine & Tobacco Research*, *22*(7), 1187–1194. https://dx.doi.org/10.1093/ntr/ntz174

Pollak, K. I., Oliver, J. A., Pieper, C., Davis, J. M., Gao, X., Noonan, D., Kennedy, D., Granados, I., & Fish, L. J. (2021). Cue-based treatment for light smokers: A proof of concept pilot. *Addictive Behaviors*, *114*, 106717. https://doi.org/10.1016/j.addbeh.2020.106717

Prochaska, J. J., Brown-Johnson, C., Baiocchi, M., Lazaro, A. S., Chieng, A., Stinson, S., & Anzai, N. (2020). Treating tobacco dependence to aid re-employment among job-seekers: A randomized controlled trial. *Preventive Medicine*, *141*, 106259. https://doi.org/10.1016/j.ypmed.2020.106259

Prochaska, J. J., Hall, S. E., Delucchi, K., & Hall, S. M. (2014). Efficacy of initiating tobacco dependence treatment in inpatient psychiatry: A randomized controlled trial. *American Journal of Public Health*, *104*(8), 1557–1565. https://doi.org/10.2105/AJPH.2013.301403

Prochaska, J. O., Butterworth, S., Redding, C. A., Burden, V., Perrin, N., Leo, M., Flaherty-Robb, M., & Prochaska, J. M. (2008). Initial efficacy of MI, TTM tailoring and HRI's with multiple behaviors for employee health promotion. *Preventive Medicine*, *46*(3), 226–231. https://doi.org/10.1016/j.ypmed.2007.11.007

Rabius, V., Pike, K. J., Wiatrek, D., & McAlister, A. (2008). Comparing internet assistance for smoking cessation: 13-month follow-up of a six-arm randomized controlled trial. *Journal of Medical Internet Research*, *10*(5), e1008. https://dx.doi.org/10.2196/jmir.1008

Ramo, D. E., Thrul, J., Chavez, K., Delucchi, K. L., & Prochaska, J. J. (2015). Feasibility and quit rates of the tobacco status project: A Facebook smoking cessation intervention for young adults. *Journal of Medical Internet Research*, *17*(12). https://dx.doi.org/10.2196/jmir.5209

Ramo, D. E., Thrul, J., Delucchi, K. L., Hall, S., Ling, P. M., Belohlavek, A., & Prochaska, J. J. (2018). A randomized controlled evaluation of the tobacco status project, a Facebook intervention for young adults. *Addiction*, *113*(9), 1683–1695. https://dx.doi.org/10.1111/add.14245

Ray, M. N. (2014). Predictors of smoking cessation among dental patients participating in a web-assisted tobacco intervention: A national dental practice-based research network study [Ph.D., The University of Alabama at Birmingham]. In *ProQuest Dissertations and Theses* (1645768019). ProQuest Central; ProQuest Dissertations & Theses Global. https://www.proquest.com/dissertations-theses/predictors-smoking-cessation-among-dental/docview/1645768019/se-2?accountid=14511

Ray, M. N., Funkhouser, E., Williams, J. H., Sadasivam, R. S., Gilbert, G. H., Coley, H. L., Rindal, D. B., & Houston, T. K. (2014). Smoking-cessation e-referrals: A national dental practice–based research network randomized controlled trial. *American Journal of Preventive Medicine*, *46*(2), 158–165. https://dx.doi.org/10.1016/j.amepre.2013.10.018

Redfern, J., Coorey, G., Mulley, J., Scaria, A., Neubeck, L., Hafiz, N., Pitt, C., Weir, K., Forbes, J., Parker, S., Bampi, F., Coenen, A., Enright, G., Wong, A., Nguyen, T., Harris, M., Zwar, N., Chow, C. K., Rodgers, A., … Peiris, D. (2020). A digital health intervention for cardiovascular disease management in primary care (CONNECT) randomized controlled trial. *Npj Digital Medicine*, *3*(1), 117. https://doi.org/10.1038/s41746-020-00325-z

Reid, R. D., Pipe, A. L., Quinlan, B., & Oda, J. (2007). Interactive voice response telephony to promote smoking cessation in patients with heart disease: A pilot study. *Patient Education and Counseling*, *66*(3), 319–326. https://dx.doi.org/10.1016/j.pec.2007.01.005

Reitzel, L. R., McClure, J. B., Cofta-Woerpel, L., Mazas, C. A., Cao, Y., Cinciripini, P. M., Vidrine, J. I., Li, Y., & Wetter, D. W. (2011). The efficacy of computer-delivered treatment for smoking cessation. *Cancer Epidemiology, Biomarkers & Prevention*, *20*(7), 1555–1557. https://doi.org/10.1158/1055-9965.EPI-11-0390

Rigotti, N. A., Chang, Y., Davis, E. M., Regan, S., Levy, D. E., Ylioja, T., Kelley, J. H. K., Notier, A. E., Gilliam, K., Douaihy, A. B., Singer, D. E., & Tindle, H. A. (2022). Comparative effectiveness of postdischarge smoking cessation interventions for hospital patients: The Helping HAND 4 randomized clinical trial. *JAMA Internal Medicine*, *182*(8), 814–824. https://doi.org/10.1001/jamainternmed.2022.2300

Rigotti, N. A., Regan, S., Levy, D. E., Japuntich, S., Chang, Y., Park, E. R., Viana, J. C., Kelley, J. H. K., Reyen, M., & Singer, D. E. (2014). Sustained care intervention and postdischarge smoking cessation among hospitalized adults: A randomized clinical trial. *JAMA*, *312*(7), 719–728. https://doi.org/10.1001/jama.2014.9237

Rigotti, N. A., Tindle, H. A., Regan, S., Levy, D. E., Chang, Y., Carpenter, K. M., Park, E. R., Kelley, J. H. K., Streck, J. M., Reid, Z. Z., Ylioja, T., Reyen, M., & Singer, D. E. (2016). A post-discharge smoking-cessation intervention for hospital patients: Helping HAND 2 randomized clinical trial. *American Journal of Preventive Medicine*, *51*(4), 597–608. https://doi.org/10.1016/j.amepre.2016.04.00510.1016/j.amepre.2016.04.005

Robinson, J. D., Cui, Y., Linares Abrego, P., Engelmann, J. M., Prokhorov, A. V., Vidrine, D. J., Shete, S., & Cinciripini, P. M. (2022). Sustained reduction of attentional bias to smoking cues by smartphone-delivered attentional bias modification training for smokers. *Psychology of Addictive Behaviors*, *36*(7), 906–919. https://dx.doi.org/10.1037/adb0000805

Rodgers, A., Corbett, T., Bramley, D., Riddell, T., Wills, M., Lin, R.-B., & Jones, M. (2005). Do u smoke after txt? Results of a randomised trial of smoking cessation using mobile phone text messaging. *Tobacco Control*, *14*(4), 255–261. https://doi.org/10.1136/tc.2005.011577

Rowan, P. J., Cofta-Woerpel, L., Mazas, C. A., Vidrine, J. I., Reitzel, L. R., Cinciripini, P. M., & Wetter, D. W. (2007). Evaluating reactivity to ecological momentary assessment during smoking cessation. *Experimental and Clinical Psychopharmacology*, *15*(4), 382. https://dx.doi.org/10.1037/1064-1297.15.4.382

Ruffin, M. T., Nease, D. E., Sen, A., Pace, W. D., Wang, C., Acheson, L. S., Rubinstein, W. S., O'Neill, S., & Gramling, R. (2011). Effect of preventive messages tailored to family history on health behaviors: The Family Healthware Impact Trial. *The Annals of Family Medicine*, *9*(1), 3–11. https://doi.org/10.1370/afm.1197

Sadasivam, R. S., Borglund, E. M., Adams, R., Marlin, B. M., & Houston, T. K. (2016). Impact of a Collective Intelligence Tailored Messaging System on Smoking Cessation: The Perspect Randomized Experiment. *J Med Internet Res*, *18*(11), e285. https://dx.doi.org/10.2196/jmir.6465

Satre, D. D., D.E. Ramo, M. Kaur, L.J. Maier, J.J. Prochaska, K.L. Delucchi, & M.C. Meacham. (2021). Efficacy of a Facebook intervention to address cigarette smoking and heavy episodic drinking: A pilot randomized controlled trial. *Alcoholism: Clinical and Experimental Research*, *45*. https://dx.doi.org/10.1111/acer.14617

Schlam, T. R., & Baker, T. B. (2020). Playing around with quitting smoking: A randomized pilot trial of mobile games as a craving response strategy. *Games for Health Journal*, *9*(1), 64–70. https://dx.doi.org/10.1089/g4h.2019.0030

Schlam, T. R., Fiore, M. C., Smith, S. S., Fraser, D., Bolt, D. M., Collins, L. M., Mermelstein, R., Piper, M. E., Cook, J. W., & Jorenby, D. E. (2016). Comparative effectiveness of intervention components for producing long-term abstinence from smoking: A factorial screening experiment. *Addiction*, *111*(1). https://doi.org/10.1111/add.13153

Schnall, R., Liu, J., Alvarez, G., Porras, T., Ganzhorn, S., Boerner, S., Huang, M.-C., Trujillo, P., & Cioe, P. (2022). A smoking cessation mobile app for persons living with HIV: Preliminary efficacy and feasibility study. *JMIR Formative Research*, *6*(8). https://dx.doi.org/10.2196/28626

Seidman, D. F., Westmaas, J. L., Goldband, S., Rabius, V., Katkin, E. S., Pike, K. J., Wiatrek, D., & Sloan, R. P. (2010). Randomized Controlled Trial of an Interactive Internet Smoking Cessation Program with Long-Term Follow-up. *Annals of Behavioral Medicine*, *39*(1), 48–60. https://doi.org/10.1007/s12160-010-9167-7

Shuter, J., Chander, G., Graham, A. L., Kim, R. S., & Stanton, C. A. (2022). Randomized trial of a web-based tobacco treatment and online community support for people with HIV attempting to quit smoking cigarettes. *Journal of Acquired Immune Deficiency Syndromes*, *90*(2). https://dx.doi.org/10.1097/QAI.0000000000002936

Shuter, J., Kim, R. S., An, L. C., & Abroms, L. C. (2020). Feasibility of a smartphone-based tobacco treatment for HIV-infected smokers. *Nicotine & Tobacco Research*, *22*(3), 398–407. https://dx.doi.org/10.1093/ntr/nty208

Shuter, J., Morales, D. A., Considine-Dunn, S. E., An, L. C., & Stanton, C. A. (2014). Feasibility and preliminary efficacy of a web-based smoking cessation intervention for HIV-infected smokers: A randomized controlled trial. *Journal of Acquired Immune Deficiency Syndromes*, *67*(1). https://dx.doi.org/ 10.1097/QAI.0000000000000226

Smits, J. A. J., Rinck, M., Rosenfield, D., Beevers, C. G., Brown, R. A., Conroy Busch, H. E., Dutcher, C. D., Perrone, A., Zvolensky, M. J., & Garey, L. (2022). Approach bias retraining to augment smoking cessation: A pilot randomized controlled trial. *Drug and Alcohol Dependence*, *238*, 109579. https://doi.org/10.1016/j.drugalcdep.2022.109579

***Spears, C. A., Abroms, L. C., Glass, C. R., Hedeker, D., Eriksen, M. P., Cottrell-Daniels, C., Tran, B. Q., & Wetter, D. W. (2019). Mindfulness-based smoking cessation enhanced with mobile technology (iQuit Mindfully): Pilot randomized controlled trial. *JMIR mHealth and uHealth*, *7*(6). https://dx.doi.org/10.2196/13059**

Squiers, L., Augustson, E., Brown, D., Kelly, B., Southwell, B., Dever, J., Dolina, S., Tzeng, J., Parvanta, S., Holt, S., Sanders, A., Zulkiewicz, B., & Hunt, Y. (2017). An experimental comparison of mobile

Field Code Changed

texting programs to help young adults quit smoking. *Health Systems*, *6*(1). https://doi.org/10.1057/s41306-016-0014-3

***Sridharan, V., Shoda, Y., Heffner, J., & Bricker, J. (2019). A pilot randomized controlled trial of a web-based growth mindset intervention to enhance the effectiveness of a smartphone app for smoking cessation. *JMIR mHealth and uHealth*, *7*(7). https://dx.doi.org/10.2196/14602**

Stoddard, J., Augustson, E., & Moser, R. (2008). Effect of adding a virtual community (bulletin board) to smokefree. Gov: Randomized controlled trial. *Journal of Medical Internet Research*, *10*(5), e1124. https://dx.doi.org/10.2196/jmir.1124

***Stoops, W. W., Dallery, J., Fields, N. M., Nuzzo, P. A., Schoenberg, N. E., Martin, C. A., Casey, B., & Wong, C. J. (2009). An internet-based abstinence reinforcement smoking cessation intervention in rural smokers. *Drug and Alcohol Dependence*, *105*(1), 56–62. https://doi.org/10.1016/j.drugalcdep.2009.06.010**

Strecher, V. J., McClure, J. B., Alexander, G. L., Chakraborty, B., Nair, V. N., Konkel, J. M., Greene, S. M., Collins, L. M., Carlier, C. C., Wiese, C. J., Little, R. J., Pomerleau, C. S., & Pomerleau, O. F. (2008). Web-based smoking-cessation programs: Results of a randomized trial. *American Journal of Preventive Medicine*, *34*(5), 373–381. https://dx.doi.org/10.1016/j.amepre.2007.12.024

Swan, G. E., McClure, J. B., Jack, L. M., Zbikowski, S. M., Javitz, H. S., Catz, S. L., Deprey, M., Richards, J., & McAfee, T. A. (2010). Behavioral counseling and varenicline treatment for smoking cessation. *American Journal of Preventive Medicine*, *38*(5), 482–490. https://doi.org/10.1016/j.amepre.2010.01.024

Tan, R. (2019). *Examining Cigarette and Craving Rates Using ACT-Based (2Morrow Quit™) and Mindfulness-Based (Craving to Quit®) Mobile Health Applications* [The University of Waikato]. https://hdl.handle.net/10289/12493

Tombor, I., Beard, E., Brown, J., Shahab, L., Michie, S., & West, R. (2019). Randomized factorial experiment of components of the SmokeFree Baby smartphone application to aid smoking cessation in pregnancy. *Translational Behavioral Medicine*, *9*(4), 583–593. https://doi.org/10.1093/tbm/iby073

Tseng, T.-Y., Krebs, P., Schoenthaler, A., Wong, S., Sherman, S., Gonzalez, M., Urbina, A., Cleland, C. M., & Shelley, D. (2017). Combining text messaging and telephone counseling to increase varenicline adherence and smoking abstinence among cigarette smokers living with HIV: A randomized controlled study. *AIDS and Behavior*, *21*(7), 1964–1974. https://doi.org/10.1007/s10461-016-1538-z

Tsoh, J., Kohn, M., & Gerbert, B. (2010). Promoting smoking cessation in pregnancy with Video Doctor plus provider cueing: A randomized trial. *Acta Obstetricia et Gynecologica Scandinavica*, *89*(4), 515–523. https://doi.org/10.3109/00016341003678419

***Tucker, J. S., Linnemayr, S., Pedersen, E. R., Shadel, W. G., Zutshi, R., DeYoreo, M., & Cabreros, I. (2021). Pilot Randomized Clinical Trial of a Text Messaging-Based Intervention for Smoking Cessation Among Young People Experiencing Homelessness. *Nicotine & Tobacco Research*, *23*(10), 1691–1698. https://dx.doi.org/10.1093/ntr/ntab055**

Unrod, M., Smith, M., Spring, B., DePue, J., Redd, W., & Winkel, G. (2007). Randomized controlled trial of a computer-based, tailored intervention to increase smoking cessation counseling by primary care physicians. *Journal of General Internal Medicine*, *22*(4), 478–484. https://doi.org/10.1007/s11606-006-0069-0

Vidrine, D. J., Frank-Pearce, S. G., Vidrine, J. I., Tahay, P. D., Marani, S. K., Chen, S., Yuan, Y., Cantor, S. B., & Prokhorov, A. V. (2019). Efficacy of mobile phone–delivered smoking cessation interventions for socioeconomically disadvantaged individuals: A randomized clinical trial. *JAMA Internal Medicine*, *179*(2), 167–174. https://dx.doi.org/10.1001/jamainternmed.2018.5713

***Vilardaga, R., Rizo, J., Palenski, P. E., Mannelli, P., Oliver, J. A., & Mcclernon, F. J. (2020). Pilot randomized controlled trial of a novel smoking cessation app designed for individuals with co-occurring tobacco use disorder and serious mental illness. *Nicotine & Tobacco Research*, *22*(9), 1533–1542. https://doi.org/10.1093/ntr/ntz202**

***Villanti, A. C., Peasley-Miklus, C., Cha, S., Schulz, J., Klemperer, E. M., LePine, S. E., West, J. C., Mays, D., Mermelstein, R., Higgins, S. T., & Graham, A. L. (2022). Tailored text message and web intervention for smoking cessation in U.S. socioeconomically-disadvantaged young adults: A randomized controlled trial. *Preventive Medicine*, *165*(Part B), 107209. https://dx.doi.org/10.1016/j.ypmed.2022.107209**

***Walker, L. (2020). *Using age-progression facial morphing technology to encourage smoking cessation in women and the role of the stress response* [Doctoral thesis (PhD), Manchester Metropolitan University]. https://e-space.mmu.ac.uk/627316/**

Webb Hooper, M., Baker, E. A., & Robinson, R. G. (2014). Efficacy of a DVD-based smoking cessation intervention for African Americans. *Nicotine & Tobacco Research*, *16*(10), 1327–1335. https://dx.doi.org/10.1093/ntr/ntu079

Webb Hooper, M., Miller, D. B., Saldivar, E., Mitchell, C., Johnson, L., Burns, M., & Huang, M.-C. (2021). Randomized controlled trial testing a video-text tobacco cessation intervention among economically disadvantaged African American adults. *Psychology of Addictive Behaviors*, *35*(7), 769. https://doi.org/10.1037/adb0000691

Webb, J., Peerbux, S., Ang, A., Siddiqui, S., Sherwani, Y., Ahmed, M., MacRae, H., Puri, H., Majeed, A., & Glasner, S. (2022). Long-Term Effectiveness of a Clinician-Assisted Digital Cognitive Behavioral Therapy Intervention for Smoking Cessation: Secondary Outcomes From a Randomized Controlled Trial. *Nicotine & Tobacco Research*, *24*(11), 1763–1772. https://dx.doi.org/10.1093/ntr/ntac113

Westmaas, J. L., Bontemps-Jones, J., Hendricks, P. S., Kim, J., & Abroms, L. C. (2018). Randomised controlled trial of stand-alone tailored emails for smoking cessation. *Tobacco Control*, *27*(2), 136. https://dx.doi.org/10.1136/tobaccocontrol-2016-053056

Wetter, D. W., McClure, J. B., Cofta-Woerpel, L., Costello, T. J., Reitzel, L. R., Businelle, M. S., & Cinciripini, P. M. (2011). A randomized clinical trial of a palmtop computer-delivered treatment for smoking relapse prevention among women. *Psychology of Addictive Behaviors*, *25*(2), 365. https://dx.doi.org/10.1037/a0022797

White, J. S., Toussaert, S., Thrul, J., Bontemps-Jones, J., Abroms, L., & Westmaas, J. L. (2020). Peer mentoring and automated text messages for smoking cessation: A randomized pilot trial. *Nicotine & Tobacco Research*, *22*(3), 371–380. https://doi.org/10.1093/ntr/ntz047

***White, M. A., Ivezaj, V., & Grilo, C. M. (2019). Evaluation of a web-based cognitive behavioral smoking cessation treatment for overweight/obese smokers. *Journal of Health Psychology*, *24*(13), 1796–1806. https://doi.org/10.1177/1359105317701560**

Whittaker, R. (2011). *Can a multimedia mobile phone programme help young people stop smoking?* (ACTRN12606000476538). https://anzctr.org.au/Trial/Registration/TrialReview.aspx?ACTRN=12606000476538

Whittaker, R., Dorey, E., Bramley, D., Bullen, C., Denny, S., Elley, C. R., Maddison, R., McRobbie, H., Parag, V., Rodgers, A., & Salmon, P. (2011). A theory-based video messaging mobile phone intervention for smoking cessation: Randomized control trial. *JMIR*, *13*(1). https://dx.doi.org/10.2196/jmir.1553

Woodruff, S. I., Conway, T. L., Edwards, C. C., Elliott, S. P., & Crittenden, J. (2007). Evaluation of an Internet virtual world chat room for adolescent smoking cessation. *Addictive Behaviors*, *32*(9), 1769–1786. https://doi.org/10.1016/j.addbeh.2006.12.008

Ybarra, M. L., Holtrop, J. S., Prescott, T. L., Rahbar, M. H., & Strong, D. (2013). Pilot RCT results of Stop My Smoking USA: A text messaging–based smoking cessation program for young adults. *Nicotine & Tobacco Research*, *15*(8), 1388–1399. https://dx.doi.org/10.1093/ntr/nts339

Zullig, L. L., Sanders, L. L., Shaw, R. J., McCant, F., Danus, S., & Bosworth, H. B. (2014). A randomised controlled trial of providing personalised cardiovascular risk information to modify health behaviour. *Journal of Telemedicine and Telecare*, *20*(3), 147–152. https://dx.doi.org/10.1177/1357633X14528446

~~C.~~D. Study characteristics of extended set of studies

Supplementary Table 1 Study characteristics of the 48 studies included in the extended analysis

<table>
<tr><th>Study</th><th>Study Type</th><th>Country</th><th>N Overall (N Intervention (I) | N Control (C))</th><th>Inclusion Criteria</th><th>Age: Mean (SD) I|C</th><th>% Female I|C</th><th>SEP Measure</th><th>% low SEP I|C</th><th>Baseline CPD: Mean (SD) I|C</th><th>Baseline Nicotine Dependence Measure: Mean (SD) I|C</th><th>Abstinence Measure</th><th>Follow-up Time</th><th>% Followed Up I|C</th></tr>
<tr><td>~~Study~~</td><td>~~Study Type~~</td><td>~~Country~~</td><td>~~N Overall (N Intervention | N Control)~~</td><td>~~Inclusion Criteria~~</td><td>~~Mean (SD) Age Intervention | Control~~</td><td>~~Percentage Female Intervention | Control~~</td><td>~~SES Measure~~</td><td>~~Percentage with low SES Intervention | Control~~</td><td>~~Baseline Mean (SD) CPD Overall Intervention | Control~~</td><td>~~Baseline Nicotine Dependence Measure: Mean (SD) Intervention | Control~~</td><td>~~Abstinence Measure~~</td><td>~~Follow-up Time~~</td><td>~~Percentage Followed Up Intervention | Control~~</td></tr>
<tr><td>Abroms (2014)</td><td>RCT</td><td>USA</td><td>503 (262 | 241)</td><td>Ages 18+, no motivational requirement, smoking at least five~~5~~ CPD</td><td>35.9 (10.7) | 35.5 (10.6)</td><td>68.7 | 62.8</td><td>Education</td><td>16.4 | 27.8</td><td>17.7 (8.1) | 16.9 (8.0)</td><td>FTND: 5.4 (2.3) | 5.3 (2.3)</td><td>Biochemically verified repeated point prevalence abstinence</td><td>24 weeks (6 months)</td><td>62.2 | 70.9</td></tr>
<tr><td>Baggett (2019)</td><td>RCT</td><td>USA</td><td>50 (25 | 25)</td><td>Ages 18+, homeless, motivated to quit, smoking at least ~~5 CPD~~five CPD, smoking at least 100 cigarettes in life-time, eCO at least 8 ppm</td><td>46.1 (9.2) | 45.1 (9.6)</td><td>60 | 56</td><td>Homelessness</td><td>100 | 100</td><td>25.4 (6.7) | 16.2 (6.3)</td><td>FTND: 4.9 (2.0) | 5.1 (1.8)</td><td>Biochemically verified ~~7 day~~7-day PPA</td><td>8 weeks (2 months)</td><td>64 | 64</td></tr>
<tr><td>Baskerville (2018)</td><td>RCT</td><td>Canada</td><td>851 (426 | 425)</td><td>Ages 19-29, motivated to quit, smoking daily</td><td>only categorical information</td><td>45.4 | 48.6</td><td>Education</td><td>39.6 | 42.6</td><td>Only categorical information</td><td>HSI: only categorical information</td><td>Self-reported sustained abstinence (3 months)</td><td>24 weeks (6 months)</td><td>43.2 | 47.6</td></tr>
<tr><td>Beckham (2019)</td><td>RCT</td><td>USA</td><td>127 (63 | 64)</td><td>Ages 18+, homeless veteran, motivated to quit, smoking at least 10 CPD</td><td>53.8 (10.2) | 55.7 (7.5)</td><td>9.5 | 4.7</td><td>Homelessness</td><td>100 | 100</td><td>Not reported</td><td>None</td><td>Biochemically verified sustained abstinence (no smoking for 7 consecutive days or at least once a week for two weeks post 2 week grace period after quit date)</td><td>24 weeks (6 months)</td><td>Not reported</td></tr>
<tr><td>Begh (2015)</td><td>RCT</td><td>UK</td><td>118 (60 | 58)</td><td>Ages 18+, signed up to smoking cessation intervention, smoking at least 10 CPD, eCO smoking at least 10ppm</td><td>46.5 (12.7) | 43.0 (12.7)</td><td>56.7 | 59.3</td><td>Education</td><td>51.7 | 63.8</td><td>21.8 (9.9) | 19.8 (8.5)</td><td>FTND: 5.3 (2.4) | 5.7 (2.1)</td><td>Biochemically verified sustained abstinence (~~Russell standard~~Russell Standard)</td><td>24 weeks (6 months)</td><td>70.0 | 60.3</td></tr>
<tr><td>Bricker (2022)</td><td>RCT</td><td>USA</td><td>2,415 (1,214 | 1,201)</td><td>Ages 18+, motivated to quit, smoking at least ~~5 CPD~~five CPD in last 12 months</td><td>38.2 (10.8) | 38.3 (11.0)</td><td>70.6 | 70.2</td><td>Income</td><td>36.8 | 34.6</td><td>Not reported</td><td>FTND: 5.8 (2.1) | 5.9 (2.0)</td><td>Self-reported sustained abstinence</td><td>52 weeks (12 months)</td><td>85.7 | 88.8</td></tr>
</table>

Formatted: Font: 8 pt, Bold

Formatted Table

Brown (2014)	RCT	UK	4,613 (2,321 | 2,292)	Ages 18+, motivated to quit, smoking daily	39.5 (13.0) | 38.8 (12.5)	63.0 | 62.3	Occupational social grade	46.9 | 46.0	18.7 (8.9) | 18.5 (9.0)	FTND [HSI]: 5.1 (2.4) | 5.0 (2.4) [2.9 (1.1) | 2.9 (1.0)]	Biochemically verified sustained abstinence (Russell Standard)	24 weeks (6 months)	70.8 | 72.9
Coleman (2022)	RCT	UK	1,002 (501 | 501)	Ages 16+, pregnant, willing to receive smoking cessation advice, smoking at least ~~5 CPD~~five CPD pre-pregnancy, smoking at least ~~1 CPD~~one CPD during pregnancy	27.1 (5.6) | 27.5 (5.7)	100 | 100	Education	68.7 | 68.1	8.6 (5.5) | 8.9 (5.5)	HSI: 1.9 (1.4) | 2.0 (1.4)	Biochemically verified sustained abstinence (Russell Standard) AND ~~7 day~~7-day PPA	36 weeks of gestation (on average 21 weeks)	61.7 | 67.3
Crane (2019)	RCT	Global (intervention in English)	28,112 (14,228 | 13,884)	Ages 18+, signed up to smoking cessation intervention, current smoking	28.7 (9.0) | 29.1 (9.4)	49.3 | 48.8	Education	62.9 | 62.9	14.6 (7.4) | 14.8 (7.6)	HSI: 2.3 (1.5) | 2.4 (1.5)	Self-reported sustained abstinence (~~Russell standard~~Russell Standard)	12 weeks (3 months)	8.5 | 6.5
Dahne (2021)	RCT	USA	51 (34 | 17)	Ages 18+, no motivational requirement, smoking at least ~~5 CPD~~five CPD on at least 20 days in last month for at least 6 months	48.2 (13.4) | 42.8 (11.2)	67.7 | 70.6	Education	20.6 | 17.7	15.4 (10.2) | 13.2 (6.8)	None	Self-reported ~~7 day~~7-day PPA	12 weeks (3 months)	70.6 | 94.1
Dahne (2022)	RCT	USA	125 (85 | 40)	Ages 30+, no COPD diagnosis, no motivational requirement, smoking at least ~~5 CPD~~five CPD on at least 20 days in last month for at least 6 months	54.1 (9.8) | 51.9 (8.5)	61.2 | 55.0	Income	50.6 | 55.0	18.1 (9.0) | 19.2 (11.4)	None	Biochemically verified ~~7 day~~7-day PPA	12 weeks (3 months)	57.7 | 62.5
Dallery (2017)	RCT	USA	94 (48 | 46)	Ages 18+, motivated to quit, smoking at least 10 CPD, last cigarette within last 24 hours, smoking daily for 2~~two~~ years or more	36.7 (11.2) | 34.9 (11.1)	56 | 54	Income	34 | 26	17.8 (7) | 18.3 (7)	FTND: 4.8 (2.0) | 4.8 (2.3)	Biochemically verified ~~7 day~~7-day PPA	24 weeks (6 months)	79.2 | 89.1
Dingle (2017)	RCT	Australia	37 (19 | 18)	Ages 18+, signed up to smoking cessation intervention, current smoking	40.4 (9.6) | 41.4 (12.4)	36.8 | 33.3	Education	47.4 | 55.6	Not reported	FTND: 4.90 (2.5) | 4.7 (2.5)	Self-reported ~~7 day~~7-day PPA	6 weeks	57.9 | 72.2
Forinash (2018)	RCT	USA	49 (23 | 26)	Ages 18+, pregnant, Medicaid eligible, motivated to quit, current smoking	not reported	100 | 100	Health insurance status	100 | 100	Not reported	None	Biochemically verified cessation (no standard)	2 weeks	60.9 | 61.5
Free (2011)	RCT	UK	5,792 (2,911 | 2,881)	Ages 16+, motivated to quit, smoking daily	36.8 (11.0) | 36.9 (11.1)	44.8 | 45.0	Occupational social grade	31.4 | 30.3	Not reported	FTND: only categorical information	Biochemically verified sustained abstinence (~~Russell standard~~Russell Standard)	24 weeks (6 months)	94.0 | 96.8
Garrison (2020)	RCT	USA	505 (245 | 260)	Ages 18+, motivated to quit, smoking at least 5	42.9 (11.6) |	69.0 | 71.2	Education	20.8 | 17.7	17.0 (8.4) | 16.6 (7.9)	None	Biochemically verified ~~7 day~~7-day PPA	24 weeks (6 months)	78.4 | 73.9

				~~CPD~~five CPD, abstinent for less than 3 months in past 12 months	40.6 (12.2)								
Haaga (2020)	RCT	USA	278 (146 | 132)	Ages 18+, no motivational requirement, smoking at least 10 CPD, smoking daily, eCO at least 9 ppm	only overall 49.8 (11.4)	47.8	Income	63.0 | 75.0	only overall 16.6 (8.1)	FTND: only overall 5.4 (2.0)	Biochemically verified ~~7 day~~7-day PPA	4 weeks (1 month)	87.7 | 89.4
Heffner (2023)	RCT	USA	49 (25 | 24)	Ages 18+, veteran, low income, no motivational requirement, smoking at least ~~5 CPD~~five CPD in last month	47.9 (13.3) | 54.8 (16.1)	20.0 | 29.2	Income	100 | 100	18 (9.1) | 15.4 (7.0)	FTND: 4.3 (2.3) | 4.2 (2.1)	Biochemically verified ~~30 day~~30-day PPA	12 weeks (3 months)	72.0 | 83.3
Herbec (2014)	RCT	UK	200 (99 | 101)	Ages 18+, pregnant, motivated to quit, smoking daily	27.6 (6.0) | 28.1 (5.8)	100 | 100	Occupational social grade	54.6 | 41.6	14.6 (5.2) | 14.7 (7.8)	FTND [HSI]: 4.6 (1.7) | 4.3 (1.6) [2.7 (1.4) | 2.7 (1.4)]	Self-reported ~~30 day~~30-day PPA	8 weeks (2 months)	63.6 | 69.3
Herbec (2019)	RCT	UK	425 (208 | 217)	Ages 18+, motivated to quit, smoking daily	33.1 (10.1) | 32.8 (11.4)	44.7 | 46.1	Education	29.8 | 32.7	15.1 (7.3) | 15.6 (7.0)	HSI: 2.6 (1.4) | 2.5 (1.4)	Self-reported sustained abstinence (Russell Standard) AND ~~7 day~~7-day PPA	26 weeks (6.5 months)	38.0 | 42.4
Jackson (2023)	RCT	Global (intervention in English)	3,143 (1,564 | 1,579)	Ages 18+, motivated to quit, current smoking	49.2 (11.6) | 48.9 (11.4)	74.0 | 75.5	Education	34.5 | 37.1	18.0 (8.7) | 18.2 (10.0)	Time to first cigarette: only categorical information	Self-reported sustained abstinence (Russell Standard)	28 weeks (7 months)	33.8 | 36.4
Joyce (2021)	RCT	USA	27 (18 | 9)	Ages 18+, pregnant/up to 3 weeks post-partum, Medicaid eligible, no motivational requirement, current smoking	27.3 (4.7)	100 | 100	Health insurance status	100 | 100	only overall 13.2 (13.8)	None	Biochemically verified (substantially more lenient standard than standard) negative cotinine test	12 weeks (3 months)	55.6 | 66.7
Kahler (2020)	RCT	USA	119 (58 | 61)	Ages 18+, drinking at heavy or at-risk levels, signed up to smoking cessation intervention, smoking daily	37.1 (10.6) | 38.6 (12.9)	71 | 69	Education	25.9 | 19.7	18.4 (10.5) | 15.9 (7.4)	FTND: 5.3 (2.3) | 4.9 (2.3)	Self-reported ~~7 day~~7-day PPA	24 weeks (6 months)	56.9 | 49.2
Kim (2020)	RCT	USA	53 (27 | 26)	Ages 18-65, HIV positive, female, motivated to quit, smoking at least ~~5 CPD~~five CPD in last 6 months	51.8 (7.0) | 50.9 (6.8)	100 | 100	Education	51.9 | 61.5	19.2 (11.4) | 15.4 (8.3)	FTND: 6.2 (2.4) | 4.7 (2.2)	Biochemically verified (substantially more lenient cut-offs than standard) sustained abstinence	12 weeks (3 months)	85.2 | 96.2
King (2022)	RCT	UK	28 (15 | 13)	Ages 16+, pregnant, no motivational requirement, current smoking, eCO at least 4 ppm	27.2 (6.1) | 24.4 (5.6)	100 | 100	Education	33 | 67	only categorical information	Time to first cigarette: only categorical information	Self-reported sustained abstinence (6 weeks; abstinence since last appointment)	12-13 weeks post due date (approx 40 weeks (10 months) post randomisation)	40 | 23

<table>
<tr><th></th><th></th><th></th><th></th><th></th><th></th><th></th><th></th><th></th><th></th><th></th><th></th><th></th><th></th></tr>
<tr><td>Klimis (2020)</td><td>RCT</td><td>Australia</td><td>369 (177 | 192)</td><td>Ages 18+, diagnosis of coronary heart disease, no motivational requirement, smoking at least <s>5 CPD</s>five CPD in last 12 months, eCO indicates smoking (cut-off not reported)</td><td>only overall 56.7 (8.9)</td><td>13.26</td><td>Education</td><td>72.4</td><td>only overall 22.1 (13.8)</td><td>None</td><td>Biochemically verified (cut-offs not reported) cessation (no standard)</td><td>24 weeks (6 months)</td><td>97.9</td></tr>
<tr><td>Leykin (2012)</td><td>RCT</td><td>Global (intervention in Spanish and English)</td><td>8,223 (4,105 | 4,118)</td><td>Ages 18+, motivated to quit, smoking at least <s>5 CPD</s>five CPD</td><td>36.4 (13.6) | 36.1 (11.4)</td><td>46.9 | 47.0</td><td>Education</td><td>23.1 | 22.8</td><td>19.6 (9.7) | 19.4 (9.9)</td><td>FTND: 5.2 (2.5) | 5.2 (2.5)</td><td>Self-reported <s>30 day</s>30-day PPA</td><td>52 weeks (12 months)</td><td>23.2 | 19.8</td></tr>
<tr><td>McClure (2015)</td><td>RCT</td><td>USA</td><td>391 (192 | 199)</td><td>Ages 18+, enrolled in substance use disorder programme for illicit drugs, no motivational requirement, current smoking</td><td>34.7 (9.8) | 33.1 (10.5)</td><td>40.6 | 45.2</td><td>Education</td><td>90.6 | 83.9</td><td>only categorical information</td><td>FTND: 3.6 (2.3) | 3.4 (2.2)</td><td>Self-reported cessation (no standard)</td><td>36 weeks (9 months)</td><td>92.0 | 88.5</td></tr>
<tr><td>McRobbie (2020)</td><td>RCT</td><td>UK and Australia</td><td>234 (116 | 118)</td><td>Ages 18+, ex-smoker 7-100 days post quit, abstinent smokers who quit 7-100 days ago</td><td>Median between 43 and 44 | Median between 44 and 46</td><td>47.4 | 48.3</td><td>Income</td><td>58.2 | 52.2</td><td>0 | 0</td><td>HSI: only categorical information</td><td>Self-reported sustained abstinence (no more than 7 consecutive days of smoking in past 6 months and not even a puff in last month)</td><td>24 weeks (6 months)</td><td>87.1 | 89.8</td></tr>
<tr><td>Mutter (2020)</td><td>RCT</td><td>USA</td><td>346 (172 | 174)</td><td>Ages 18+, interested in smoking reduction or cessation, smoking at least some days in past 14 days</td><td>34.2 (10.5) | 34.5 (10.5)</td><td>47.7 | 42.5</td><td>Education</td><td>12.8 | 17.8</td><td>12.1 (9.0) | 10.9 (8.3)</td><td>Five-item version of the Cigarette Dependence Scale (CDS-5): 17.0 (4.1) | 16.4 (4.7)</td><td>Self-reported <s>7 day</s>7-day PPA</td><td>4 weeks (1 month)</td><td>75.6 | 77.0</td></tr>
<tr><td>Naughton (2014)</td><td>RCT</td><td>UK</td><td>602 (299 | 303)</td><td>Ages 18-75, motivated to quit, smoking at least <s>1 CPD</s>one CPD, smoked in last 7 days</td><td>42.3 | 41.3</td><td>53.2 | 52.1</td><td>Occupational social grade</td><td>46.8 | 40.9</td><td>18.4 (7.9) | 18.2 (8.2)</td><td>None</td><td>Biochemically verified sustained abstinence</td><td>24 weeks (6 months)</td><td>76.6 | 78.6</td></tr>
<tr><td>Naughton (2017)</td><td>RCT</td><td>UK</td><td>407 (203 | 204)</td><td>Ages 16+, pregnant, willing to receive cessation advice, smoking at least <s>5 CPD</s>five CPD pre-pregnancy, smoking at least <s>1 CPD</s>one CPD during pregnancy</td><td>26.6 (5.7) | 26.4 (5.7)</td><td>100 | 100</td><td>Education</td><td>75.9 | 73.5</td><td>9.0 (5.9) | 9.4 (6.1)</td><td>HSI: only categorical information</td><td>Biochemically verified sustained abstinence (Russell Standard)</td><td>36 weeks of gestation (on average 21-22 weeks)</td><td>63.6 | 64.7</td></tr>
<tr><td>Naughton (2023)</td><td>RCT</td><td>UK</td><td>209 (104 | 105)</td><td>Ages 16+, motivated to quit, smoking at least seven<s>7</s> cigarettes per week</td><td>39.6 (10.0) | 42.6 (10.0)</td><td>56.7 | 54.3</td><td>Occupational social grade</td><td>23.1 | 11.4</td><td>15.4 (7.6) | 15.5 (6.5)</td><td>HSI: only categorical information</td><td>Biochemically verified sustained abstinence (Russell Standard)</td><td>24 weeks (6 months)</td><td>78 | 75</td></tr>
<tr><td>Palmer (2023)</td><td>RCT</td><td>USA</td><td>34 (19 | 15)</td><td>Ages 18+, motivated to quit, smoking at least <s>5</s></td><td>43 (12) | 45 (13)</td><td>36.8 | 40.0</td><td>Education</td><td>31.6 | 33.3</td><td>17.1 (5.9) | 19.4 (9.9)</td><td>Time to first cigarette: only</td><td>Self-reported 3 day PPA</td><td>4 weeks (1 month)</td><td>100 | 100</td></tr>
</table>

				~~CPD~~five CPD, smoking at least 25 days in past month, smoking for over 1 year						categorical information			
Pbert (2020)	matched cluster RCT	USA	96 (48 | 48)	~~Ages~~ High school students~~Teenager~~, motivated to quit, smoking at least ~~5 CPD~~five CPD	16.9 (1.1) | 16.7 (1.1)	60.4 | 54.2	Income	68.8 | 68.8	4.8 (3.1) | 4.9 (4.2)	None	Biochemically verified ~~7 day~~7-day PPA	24 weeks (6 months)	92 | 98
Shuter (2022)	RCT	USA	506 (255 | 251)	Ages 18+, HIV positive, motivated to quit, at least a puff in the last ~~7 day~~7-days, smoking at least 100 cigarettes in life-time	50.5 (10.2) | 50.0 (10.6)	41.6 | 43.8	Income	89.8 | 90.0	11.3 (8.7) | 11.8 (8.9)	Modified Fagerström Tolerance Questionnaire: only categorical information	Biochemically verified ~~7 day~~7-day PPA	24 weeks (6 months)	74.1 | 75.3
Smits (2022)	RCT	USA	96 (46 | 50)	Ages 18+, motivated to quit, smoking at least ~~5 CPD~~five CPD in last 12 months	42.8 (10.8) | 43.3 (10.7)	54.3 | 56.0	Education	8.7 | 10.0	13.5 (6.1) | 15.5 (7.8)	FTND: 4.4 (2.4) | 5.1 (2.2)	Biochemically verified ~~7 day~~7-day PPA	12 weeks (3 months)	80.4 | 76.0
Spears (2019)	RCT	USA	36 (3 | 33)	Ages 18+, motivated to quit, abstinent smokers who quit 7-100 days ago	45.6 (12.4) | 45.6 (12.0)	45 | 61	Income	48 | 33	14.4 (9.4) | 18.8 (9.3)	Time to first cigarette: only categorical information	Biochemically verified ~~7 day~~7-day PPA	7 weeks	86.8 | 90.9
Sridharan (2019)	RCT	USA	398 (199 | 199)	Ages 18+, motivated to quit, smoking at least ~~5 CPD~~five CPD, smoking for smoking at least 1 year	42.1 (12.0) | 42.0 (12.5)	59 | 58	Income	31.7 | 29.6	19.1 (15.9) | 19.0 (16.5)	FTND: 5.8 (2.0) | 5.9 (2.1)	Biochemically verified ~~30 day~~30-day PPA	8 weeks (2 months)	90.5 | 93.0
Stoops (2009)	RCT	USA	68 (35 | 33)	Ages 18+, no motivational requirement, smoking at least 10 CPD, eCO at least 8ppm	38 |40	74.3 | 75.8	Education	20.0 | 42.4	30 | 30	FTND: 5 | 5	Biochemically verified ~~7 day~~7-day PPA	5 weeks	82.9 | 87.9
Stretcher (2008)	Randomised fractional factorial trial -> RCT	USA	944 (488 | 456)	Ages 21-70, motivated to quit, smoking at least 10 CPD, smoked in last ~~7 day~~7-days, smoking at least 100 cigarettes in life-time	only overall 46.3	59.5	Education	36.5 | 36.6	only overall 21.8	None	Self-reported ~~7 day~~7-day PPA	24 weeks (6 months)	50.6
Tan (2019)	RCT	Global (intervention in English)	10 (6 | 4)	Ages 18+, motivated to quit, smoking at least 10 CPD, HSI of at least 4	only categorical information	50 | 50	Education	16.7 | 0	15.8 (4.1) | 21.3 (8.5)	None	Self-reported cessation (no standard)	4 weeks (1 month)	83.3 | 25.0
Tucker (2021)	cluster-crossover RCT	USA	81 (43 | 38)	Ages 18-25, homeless, motivated to quit, smoking at least ~~5 CPD~~five CPD on at least 20 days in past month	22.2 (1.9) | 23.0 (1.7)	22.5 | 13.9	Homelessness	100 | 100	8.2 (7.1) | 9.3 (5.7)	None	Self-reported sustained abstinence	12 weeks (3 months)	83.7 | 79.0

Vilardaga (2020)	RCT	USA	62 (33 | 29)	Ages 18+, diagnosis of serious mental illness, motivated to quit, smoking at least ~~5 CPD~~five CPD, eCO at least 6ppm	46.1 (11.3) | 45.6 (10.9)	64 | 55	Education	33.3 | 28.6	21 (15.5) | 14 (6.4)	FTND: 5.2 (2.6) | 4.7 (2.3)	Biochemically verified ~~7 day~~7-day PPA	16 weeks (4 months)	100 | 96.6
Villanti (2022)	RCT	USA	437 (229 | 208)	Ages 18-30, socially disadvantaged, motivated to quit, current smoking, smoking at least 100 cigarettes in lifetime	only overall 25.6 (3.3)	86.6 | 82.5	Subjective financial status	100 | 100	12.2 (10.7) | 12.7 (10.3)	FTND: 1.9 (1.3) | 2.1 (1.2)	Biochemically verified (cut-offs not reported) ~~30 day~~30-day PPA	12 weeks (3 months)	48.5 | 68.3
Walker (2020)	RCT	UK	73 (50 | 23)	Ages 18-55, female, no motivational requirement, smoking at least 1 cigarette per week	25.5 (7.8) | 25.3 (7.5)	100 | 100	Education	2 | 0	only categorical information	None	Self-reported ~~7 day~~7-day PPA	12 weeks (3 months)	76.0 | 69.6
White (2019)	RCT	USA	54 (27 | 27)	Ages 18+, overweight or obesity, no motivational requirement, smoking at least 10 CPD, less than 3~~three~~ consecutive months of abstinence in past year	46.4 (11.6) | 45.4 (9.6)	74.1 | 70.4	Education	66.7 | 70.4	18.9 (5.7) | 20.5 (10.7)	None	Biochemically verified (substantially more lenient cut-offs than standard) sustained abstinence	24 weeks (6 months)	96.3 | 66.7
Ybarra (2013)	RCT	USA	164 (101 | 63)	Ages 18-25, motivated to quit, at least 4 CPD on at least 6 days per week, at least 24 cigarettes per week	21.6 (2.1) | 21.6 (2.1)	43.6 | 44.4	Education	57.4 | 60.3	12.4 (6.3) | 11.9 (5.7)	FTND: only categorical information	Self-reported and confirmed by significant other sustained abstinence (~~Russell standard~~Russell Standard)	12 weeks (3 months)	80.2 | 81.0

CPD = Cigarettes per day, eCO = expired carbon monoxide, FTND = Fagerström Test for Nicotine Dependence, HIV = Human immunodeficiency virus, HSI = Heaviness of smoking index, PPM = Parts per million, RCT = Randomised controlled trial, SD = Standard deviation, SEP = Socioeconomic position, UK = United Kingdom of Great Britain and Northern Ireland, USA = United States of America

Formatted: Keep with next

Formatted: Caption

~~D.~~E. Risk of bias: individual studies and domains

Supplementary Table 2 Risk of bias of individual studies across domains; D1: Randomization process; D2: Timing of identification or recruitment of participants; D3: Deviations from intended interventions; D4: Missing outcome data; D5: Measurement of the outcome; D6: Selection of the reported results.
The domain "Timing of identification or recruitment of participants" is only applicable to cluster randomised trials, and therefore was relevant for only two of the included studies (as indicated by the NA and grey colour for the remaining studies).

**Study**	**D1**	**D2**	**D3**	**D4**	**D5**	**D6**	**Overall Bias**
Abroms (2014)	Some concerns	NA	Some concerns	Low	Low	Some concerns	**Some concerns**
Baggett (2019)	Low	NA	Low	Low	Low	Low	**Low**
Baskerville (2018)	Low	NA	Some concerns	Low	Low	High	**High**
Beckham (2019)	Some concerns	NA	Some concerns	Some concerns	Low	Low	**Some concerns**
Begh (2015)	Low	NA	Low	Low	Low	Low	**Low**
Bricker (2022)	Low	NA	Low	Low	Low	Low	**Low**
Brown (2014)	Low	NA	Low	Low	Low	Low	**Low**
Coleman (2022)	Low	NA	Low	Low	Low	Low	**Low**
Crane (2019)	Low	NA	Low	High	Low	Some concerns	**High**
Dahne (2021)	Low	NA	Low	High	Low	Low	**High**
Dahne (2022)	Low	NA	Low	Low	Low	Low	**Low**
Dallery (2017)	Low	NA	Low	Low	Low	Low	**Low**
Dingle (2017)	Low	NA	Low	Low	Low	Low	**Low**
Forinash (2018)	Low	NA	Low	Low	High	Some concerns	**High**
Free (2011)	Low	NA	Low	Low	Low	Low	**Low**
Garrison (2020)	Low	NA	Low	Low	Low	Low	**Low**
Haaga (2020)	Some concerns	NA	Low	Low	Low	Low	**Some concerns**
Heffner (2023)	Low	NA	Low	Low	Low	Low	**Low**

Herbec (2014)	Low	NA	Low	Low	Low	Low	**Low**
Herbec (2019)	Low	NA	Low	High	Low	Low	**High**
Jackson (2023)	Low	NA	Low	Some concerns	Low	Low	**Some concerns**
Joyce (2021)	Some concerns	NA	Some concerns	Low	High	Low	**High**
Kahler (2020)	Low	NA	Low	Some concerns	Low	Low	**Some concerns**
Kim (2020)	Low	NA	Low	Low	Low	Low	**Low**
King (2022)	Low	NA	Low	Some concerns	Some concerns	Low	**Some concerns**
Klimis (2020)	Low	NA	Low	Low	Low	Some concerns	**Some concerns**
Leykin (2012)	Low	NA	Low	High	Low	Low	**High**
McClure (2015)	Low	NA	Low	Low	High	Low	**High**
McRobbie (2020)	Low	NA	Low	Low	Low	Low	**Low**
Mutter (2020)	Low	NA	Low	Low	Low	Low	**Low**
Naughton (2014)	Low	NA	Low	Low	Low	Low	**Low**
Naughton (2017)	Low	NA	Low	Low	Low	Low	**Low**
Naughton (2023)	Low	NA	Low	Low	Low	Low	**Low**
Palmer (2023)	Some concerns	NA	High	Low	High	High	**High**
Pbert (2020)	Some concerns	Some concerns	High	Low	Low	Some concerns	**High**
Shuter (2022)	Low	NA	Low	Low	Low	Low	**Low**
Smits (2022)	Low	NA	Low	Low	Low	Low	**Low**
Spears (2019)	Some concerns	NA	Low	Low	Low	Low	**Some concerns**
Sridharan (2019)	Some concerns	NA	Low	Low	Low	Low	**Some concerns**
Stoops (2009)	Some concerns	NA	Low	Low	Low	Low	**Some concerns**
Stretcher (2008)	Low	NA	Some concerns	High	Low	Some concerns	**High**

Tan (2019)	High	NA	Low	High	High	Low	**High**
Tucker (2021)	Low	Some concerns	Low	Low	Low	Low	**Some concerns**
Vilardaga (2020)	Some concerns	NA	Low	Low	Low	Low	**Some concerns**
Villanti (2022)	Low	NA	Low	Some concerns	Low	Low	**Some concerns**
Walker (2020)	Low	NA	Low	Low	Low	Low	**Low**
White (2019)	Some concerns	NA	Low	High	Low	Low	**High**
Ybarra (2013)	High	NA	Low	Low	Low	Low	**High**

~~E.~~F. Risk of bias: summary of extended set of studies

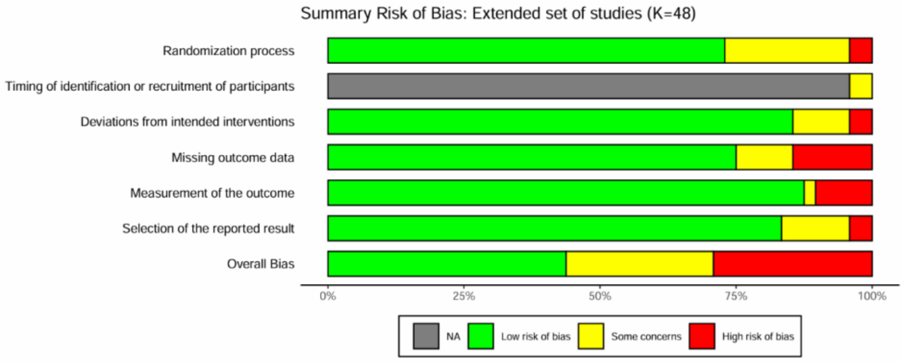

Supplementary Figure 1 Risk of B~~b~~ias for the studies included in the main analyses. The domain "Timing of identification or recruitment of participants" is only applicable to cluster randomised trials, and therefore was relevant for only two of the included studies (as indicated by the grey colour for the remaining studies).

~~F.~~G. BCTs in the intervention and control conditions

Table 3 BCTs present in intervention and comparator conditions of the k=29 studies included in the main analysis. "None" indicates that there was no comparator intervention or that the comparator was treatment as usual.

Study	BCTs in Intervention Condition	BCTs in Comparator Condition
Baskerville (2018)	1.1 Goal setting (behaviour); 1.9 Commitment; 2.2 Feedback on behaviour; 2.3 Self-monitoring of behaviour; 3.1 Social support (unspecified); 4.2 Information about antecedents; 5.1 Information about health consequences; 5.2 Salience of consequences; 5.3 Information about social and environmental consequences; 6.2 Social comparison; 9.1 Credible source; 10.4 Social reward; 10.5 Social incentive; 11.1 Pharmacological support; 11.2 Reduce negative emotions; 12.3 Avoidance/reducing exposure to cues for the behaviour; 12.4 Distraction	1.1 Goal setting (behaviour); 1.2 Problem solving; 1.4 Action planning; 1.9 Commitment; 3.1 Social support (unspecified); 4.2 Information about antecedents; 5.1 Information about health consequences; 5.3 Information about social and environmental consequences; 5.6 Information about emotional consequences; 8.2 Behaviour substitution; 8.7 Graded tasks; 9.3 Comparative imagining of future outcomes; 10.7 Self-incentive; 10.9 Self-reward; 11.1 Pharmacological support; 11.2 Reduce negative emotions; 11.3 Conserving mental resources; 12.3 Avoidance/reducing exposure to cues for the behaviour; 12.4 Distraction; 13.2 Framing/reframing; 13.5 Identity associated with changed behaviour; 15.1 Verbal persuasion about capability; 15.3 Focus on past success; 15.4 Self-talk
Begh (2015)	1.1 Goal setting (behaviour); 3.1 Social support (unspecified); 4.1 Instruction on how to perform the behaviour; 7.8 Associative learning; 11.1 Pharmacological support	1.1 Goal setting (behaviour); 3.1 Social support (unspecified); 4.1 Instruction on how to perform the behaviour; 11.1 Pharmacological support
Brown (2014)	1.1 Goal setting (behaviour); 1.2 Problem solving; 1.4 Action planning; 1.5 Review behaviour goal(s); 1.6 Discrepancy between current behaviour and goal; 1.8 Behavioural contract; 1.9 Commitment; 2.2 Feedback on behaviour; 2.3 Self-monitoring of behaviour; 2.7 Feedback on outcome(s) of behaviour; 3.1 Social support (unspecified); 3.3 Social support (emotional); 4.1 Instruction on how to perform the behaviour; 4.2 Information about antecedents; 4.3 Re-attribution; 5.1 Information about health consequences; 5.2 Salience of consequences; 5.3 Information about social and environmental consequences; 5.4 Monitoring of emotional consequences; 5.5 Anticipated regret; 5.6 Information about emotional consequences; 6.2 Social comparison; 8.1 Behavioural practice/rehearsal; 8.2 Behaviour substitution; 8.4 Habit reversal; 9.1 Credible source; 9.2 Pros and cons; 9.3 Comparative imagining of future outcomes; 10.4 Social reward; 10.9 Self-reward; 11.1 Pharmacological support; 11.2 Reduce negative emotions; 11.3 Conserving mental resources; 12.1 Restructuring the physical	1.1 Goal setting (behaviour); 1.4 Action planning; 1.9 Commitment; 2.3 Self-monitoring of behaviour; 3.1 Social support (unspecified); 4.1 Instruction on how to perform the behaviour; 4.2 Information about antecedents; 4.3 Re-attribution; 5.1 Information about health consequences; 5.3 Information about social and environmental consequences; 5.6 Information about emotional consequences; 8.2 Behaviour substitution; 8.4 Habit reversal; 11.1 Pharmacological support; 11.2 Reduce negative emotions; 11.3 Conserving mental

	environment; 12.2 Restructuring the social environment; 12.3 Avoidance/reducing exposure to cues for the behaviour; 12.4 Distraction; 12.5 Adding objects to the environment; 12.6 Body changes; 13.1 Identification of self-as role model; 13.2 Framing/reframing; 13.5 Identity associated with changed behaviour; 15.1 Verbal persuasion about capability; 15.2 Mental rehearsal of successful performance; 15.3 Focus on past success; 16.1 Imaginary punishment; 16.2 Imaginary reward	resources; 12.1 Restructuring the physical environment; 12.3 Avoidance/reducing exposure to cues for the behaviour; 12.4 Distraction; 15.3 Focus on past success
Coleman (2022)	1.1 Goal setting (behaviour); 1.2 Problem solving; 1.4 Action planning; 1.6 Discrepancy between current behaviour and goal; 1.7 Review outcome goal(s); 1.9 Commitment; 2.2 Feedback on behaviour; 3.1 Social support (unspecified); 3.3 Social support (emotional); 4.2 Information about antecedents; 5.1 Information about health consequences; 5.2 Salience of consequences; 5.3 Information about social and environmental consequences; 5.5 Anticipated regret; 5.6 Information about emotional consequences; 6.2 Social comparison; 8.1 Behavioural practice/rehearsal; 8.2 Behaviour substitution; 8.4 Habit reversal; 9.1 Credible source; 9.2 Pros and cons; 9.3 Comparative imagining of future outcomes; 10.4 Social reward; 10.7 Self-incentive; 10.9 Self-reward; 11.2 Reduce negative emotions; 11.3 Conserving mental resources; 12.1 Restructuring the physical environment; 12.2 Restructuring the social environment; 12.3 Avoidance/reducing exposure to cues for the behaviour; 12.4 Distraction; 13.1 Identification of self-as role model; 13.2 Framing/reframing; 13.5 Identity associated with changed behaviour; 15.1 Verbal persuasion about capability; 15.2 Mental rehearsal of successful performance; 15.3 Focus on past success; 15.4 Self-talk	None
Crane (2019)	1.1 Goal setting (behaviour); 1.2 Problem solving; 1.4 Action planning; 1.5 Review behaviour goal(s); 1.8 Behavioural contract; 1.9 Commitment; 2.2 Feedback on behaviour; 2.3 Self-monitoring of behaviour; 2.4 Self-monitoring of outcome(s) of behaviour; 3.1 Social support (unspecified); 3.3 Social support (emotional); 4.2 Information about antecedents; 5.1 Information about health consequences; 5.2 Salience of consequences; 5.3 Information about social and environmental consequences; 5.4 Monitoring of emotional consequences; 5.5 Anticipated regret; 5.6 Information about emotional consequences; 6.2 Social comparison; 7.1 Prompts/cues; 8.2 Behaviour substitution; 9.1 Credible source; 9.2 Pros and cons; 10.4 Social reward; 10.9 Self-reward; 11.1 Pharmacological support; 11.2 Reduce negative emotions; 11.3 Conserving mental resources; 12.1 Restructuring the physical environment; 12.2 Restructuring the social environment; 12.3 Avoidance/reducing exposure to cues for the behaviour; 12.4 Distraction; 13.2 Framing/reframing; 13.5 Identity associated with changed behaviour; 15.1 Verbal persuasion about capability; 15.2 Mental rehearsal of successful performance; 15.3 Focus on past success; 15.4 Self-talk	1.1 Goal setting (behaviour); 1.2 Problem solving; 1.4 Action planning; 1.5 Review behaviour goal(s); 2.2 Feedback on behaviour; 2.3 Self-monitoring of behaviour; 2.4 Self-monitoring of outcome(s) of behaviour; 3.1 Social support (unspecified); 3.3 Social support (emotional); 4.2 Information about antecedents; 5.1 Information about health consequences; 5.2 Salience of consequences; 5.3 Information about social and environmental consequences; 5.4 Monitoring of emotional consequences; 5.5 Anticipated regret; 5.6 Information about emotional consequences; 6.2 Social comparison; 8.2 Behaviour substitution; 9.1 Credible source; 9.2 Pros and cons; 10.4 Social reward; 10.9 Self-reward; 11.1 Pharmacological support; 11.2 Reduce negative emotions; 11.3 Conserving mental resources; 12.1 Restructuring the physical environment; 12.2 Restructuring the social environment; 12.3 Avoidance/reducing

		exposure to cues for the behaviour; 12.4 Distraction; 13.5 Identity associated with changed behaviour; 15.1 Verbal persuasion about capability
Dallery (2017)	1.1 Goal setting (behaviour); 2.6 Biofeedback; 4.1 Instruction on how to perform the behaviour; 6.1 Demonstration of the behaviour; 8.7 Graded tasks; 10.1 Material incentive (behaviour); 10.2 Material reward (behaviour); 14.3 Remove reward; 14.4 Reward approximation; 14.9 Reduce reward frequency	1.1 Goal setting (behaviour); 2.6 Biofeedback; 4.1 Instruction on how to perform the behaviour; 6.1 Demonstration of the behaviour; 8.7 Graded tasks
Dingle (2017)	1.2 Problem solving; 1.4 Action planning; 4.2 Information about antecedents; 5.6 Information about emotional consequences; 8.2 Behaviour substitution; 11.2 Reduce negative emotions;	None
Garrison (2020)	1.1 Goal setting (behaviour); 1.2 Problem solving; 1.5 Review behaviour goal(s); 2.3 Self-monitoring of behaviour; 3.1 Social support (unspecified); 4.1 Instruction on how to perform the behaviour; 4.2 Information about antecedents; 4.3 Re-attribution; 4.4 Behavioural experiments; 5.1 Information about health consequences; 5.4 Monitoring of emotional consequences; 6.3 Information about others' approval; 7.1 Prompts/cues; 8.1 Behavioural practice/rehearsal; 8.2 Behaviour substitution; 8.7 Graded tasks; 9.1 Credible source; 9.2 Pros and cons; 11.1 Pharmacological support; 11.2 Reduce negative emotions; 13.2 Framing/reframing; 15.1 Verbal persuasion about capability; 15.3 Focus on past success; 15.4 Self-talk; 16.2 Imaginary reward	1.1 Goal setting (behaviour); 2.3 Self-monitoring of behaviour; 7.1 Prompts/cues; 11.1 Pharmacological support
Haaga (2020)	3.1 Social support (unspecified); 5.1 Information about health consequences; 5.2 Salience of consequences; 5.5 Anticipated regret; 16.1 Imaginary punishment	3.1 Social support (unspecified); 5.1 Information about health consequences
Herbec (2014)	1.1 Goal setting (behaviour); 1.2 Problem solving; 1.4 Action planning; 1.5 Review behaviour goal(s); 1.6 Discrepancy between current behaviour and goal; 1.8 Behavioural contract; 1.9 Commitment; 2.2 Feedback on behaviour; 2.3 Self-monitoring of behaviour; 2.7 Feedback on outcome(s) of behaviour; 3.1 Social support (unspecified); 3.3 Social support (emotional); 4.1 Instruction on how to perform the behaviour; 4.2 Information about antecedents; 4.3 Re-attribution; 5.1 Information about health consequences; 5.2 Salience of consequences; 5.3 Information about social and environmental consequences; 5.4 Monitoring of emotional consequences; 5.5 Anticipated regret; 5.6 Information about emotional consequences; 6.2 Social comparison; 8.1 Behavioural practice/rehearsal; 8.2 Behaviour substitution; 8.4 Habit reversal; 9.1 Credible source; 9.2 Pros and cons; 9.3 Comparative imagining of future outcomes; 10.4 Social reward; 10.9 Self-reward; 11.1 Pharmacological support; 11.2 Reduce negative emotions; 11.3 Conserving mental resources; 12.1 Restructuring the physical environment; 12.2 Restructuring the social environment; 12.3 Avoidance/reducing exposure to cues for the behaviour; 12.4 Distraction; 12.5 Adding objects to the environment; 12.6 Body changes; 13.1 Identification of self-as role model; 13.2 Framing/reframing; 13.5 Identity associated with changed behaviour; 15.1 Verbal persuasion about capability; 15.2 Mental rehearsal of successful performance; 15.3 Focus on past success; 16.1 Imaginary punishment; 16.2 Imaginary reward	1.1 Goal setting (behaviour); 1.4 Action planning; 1.9 Commitment; 2.3 Self-monitoring of behaviour; 3.1 Social support (unspecified); 4.1 Instruction on how to perform the behaviour; 4.2 Information about antecedents; 4.3 Re-attribution; 5.1 Information about health consequences; 5.3 Information about social and environmental consequences; 5.6 Information about emotional consequences; 8.2 Behaviour substitution; 8.4 Habit reversal; 11.1 Pharmacological support; 11.2 Reduce negative emotions; 11.3 Conserving mental resources; 12.1 Restructuring the physical environment; 12.3 Avoidance/reducing exposure to cues for the behaviour; 12.4 Distraction; 15.3 Focus on past success
Herbec (2019)	1.1 Goal setting (behaviour); 1.3 Goal setting (outcome); 1.6 Discrepancy between current behaviour and goal; 1.9 Commitment; 2.2 Feedback on behaviour; 2.4 Self-monitoring of outcome(s) of	1.1 Goal setting (behaviour); 1.3 Goal setting (outcome); 1.6 Discrepancy between current

	behaviour; 2.7 Feedback on outcome(s) of behaviour; 3.1 Social support (unspecified); 4.2 Information about antecedents; 5.1 Information about health consequences; 5.2 Salience of consequences; 5.3 Information about social and environmental consequences; 5.5 Anticipated regret; 5.6 Information about emotional consequences; 6.2 Social comparison; 9.1 Credible source; 9.3 Comparative imagining of future outcomes; 10.3 Non-specific reward; 11.1 Pharmacological support; 11.2 Reduce negative emotions; 11.3 Conserving mental resources; 12.1 Restructuring the physical environment; 12.2 Restructuring the social environment; 12.3 Avoidance/reducing exposure to cues for the behaviour; 12.4 Distraction; 13.5 Identity associated with changed behaviour; 15.1 Verbal persuasion about capability; 15.4 Self-talk	behaviour and goal; 1.9 Commitment; 2.2 Feedback on behaviour; 2.4 Self-monitoring of outcome(s) of behaviour; 3.1 Social support (unspecified); 4.2 Information about antecedents; 5.1 Information about health consequences; 5.2 Salience of consequences; 5.3 Information about social and environmental consequences; 9.1 Credible source; 11.1 Pharmacological support; 11.3 Conserving mental resources; 12.1 Restructuring the physical environment; 12.2 Restructuring the social environment; 12.3 Avoidance/reducing exposure to cues for the behaviour; 13.5 Identity associated with changed behaviour; 15.1 Verbal persuasion about capability
Jackson (2023)	1.1 Goal setting (behaviour); 1.2 Problem solving; 1.4 Action planning; 1.5 Review behaviour goal(s); 1.8 Behavioural contract; 1.9 Commitment; 2.2 Feedback on behaviour; 2.3 Self-monitoring of behaviour; 2.4 Self-monitoring of outcome(s) of behaviour; 3.1 Social support (unspecified); 3.3 Social support (emotional); 4.2 Information about antecedents; 5.1 Information about health consequences; 5.2 Salience of consequences; 5.3 Information about social and environmental consequences; 5.4 Monitoring of emotional consequences; 5.5 Anticipated regret; 5.6 Information about emotional consequences; 6.2 Social comparison; 7.1 Prompts/cues; 8.2 Behaviour substitution; 9.1 Credible source; 9.2 Pros and cons; 10.4 Social reward; 10.9 Self-reward; 11.1 Pharmacological support; 11.2 Reduce negative emotions; 11.3 Conserving mental resources; 12.1 Restructuring the physical environment; 12.2 Restructuring the social environment; 12.3 Avoidance/reducing exposure to cues for the behaviour; 12.4 Distraction; 13.2 Framing/reframing; 13.5 Identity associated with changed behaviour; 15.1 Verbal persuasion about capability; 15.2 Mental rehearsal of successful performance; 15.3 Focus on past success; 15.4 Self-talk	1.1 Goal setting (behaviour); 1.9 Commitment; 2.3 Self-monitoring of behaviour; 5.1 Information about health consequences
Kahler (2020)	1.1 Goal setting (behaviour); 1.2 Problem solving; 1.4 Action planning; 1.9 Commitment; 2.2 Feedback on behaviour; 2.3 Self-monitoring of behaviour; 3.1 Social support (unspecified); 3.2 Social support (practical); 3.3 Social support (emotional); 4.1 Instruction on how to perform the behaviour; 4.2 Information about antecedents; 4.4 Behavioural experiments; 5.1 Information about health consequences; 5.2 Salience of consequences; 5.3 Information about social and environmental consequences; 5.6 Information about emotional consequences; 6.2 Social comparison; 7.1 Prompts/cues; 8.1 Behavioural practice/rehearsal; 8.2 Behaviour substitution; 8.4 Habit reversal; 8.7 Graded tasks; 9.1 Credible source; 10.4 Social reward; 11.1 Pharmacological support; 11.2 Reduce negative emotions; 11.3 Conserving mental resources; 12.1 Restructuring the physical environment; 12.2 Restructuring the social environment; 12.3 Avoidance/reducing exposure to cues for the	1.1 Goal setting (behaviour); 1.2 Problem solving; 1.4 Action planning; 1.9 Commitment; 2.2 Feedback on behaviour; 2.3 Self-monitoring of behaviour; 3.1 Social support (unspecified); 3.2 Social support (practical); 3.3 Social support (emotional); 4.1 Instruction on how to perform the behaviour; 4.2 Information about antecedents; 4.4 Behavioural experiments; 5.1 Information about health consequences; 5.2 Salience of consequences; 5.3 Information about social and

	behaviour; 12.4 Distraction; 12.5 Adding objects to the environment; 15.1 Verbal persuasion about capability; 16.2 Imaginary reward	environmental consequences; 5.6 Information about emotional consequences; 6.2 Social comparison; 7.1 Prompts/cues; 8.1 Behavioural practice/rehearsal; 8.2 Behaviour substitution; 8.4 Habit reversal; 8.7 Graded tasks; 9.1 Credible source; 10.4 Social reward; 11.1 Pharmacological support; 11.2 Reduce negative emotions; 11.3 Conserving mental resources; 12.1 Restructuring the physical environment; 12.2 Restructuring the social environment; 12.3 Avoidance/reducing exposure to cues for the behaviour; 12.4 Distraction; 12.5 Adding objects to the environment; 15.1 Verbal persuasion about capability; 16.2 Imaginary reward
King (2022)	1.1 Goal setting (behaviour); 1.9 Commitment; 2.3 Self-monitoring of behaviour; 3.1 Social support (unspecified); 3.3 Social support (emotional); 4.2 Information about antecedents; 5.1 Information about health consequences; 5.2 Salience of consequences; 5.3 Information about social and environmental consequences; 5.5 Anticipated regret; 5.6 Information about emotional consequences; 6.1 Demonstration of the behaviour; 6.2 Social comparison; 6.3 Information about others' approval; 8.2 Behaviour substitution; 8.7 Graded tasks; 9.1 Credible source; 10.7 Self-incentive; 10.9 Self-reward; 11.1 Pharmacological support; 11.2 Reduce negative emotions; 12.1 Restructuring the physical environment; 12.2 Restructuring the social environment; 12.3 Avoidance/reducing exposure to cues for the behaviour; 12.4 Distraction; 13.1 Identification of self-as role model; 15.1 Verbal persuasion about capability; 15.3 Focus on past success; 15.4 Self-talk; 16.3 Vicarious consequences	None
McRobbie (2020)	1.1 Goal setting (behaviour); 1.2 Problem solving; 1.4 Action planning; 1.9 Commitment; 2.3 Self-monitoring of behaviour; 3.1 Social support (unspecified); 3.3 Social support (emotional); 5.1 Information about health consequences; 5.2 Salience of consequences; 5.3 Information about social and environmental consequences; 5.4 Monitoring of emotional consequences; 5.6 Information about emotional consequences; 7.1 Prompts/cues; 7.7 Exposure; 8.1 Behavioural practice/rehearsal; 8.2 Behaviour substitution; 8.4 Habit reversal; 9.1 Credible source; 9.2 Pros and cons; 10.4 Social reward; 10.7 Self-incentive; 10.9 Self-reward; 11.1 Pharmacological support; 11.2 Reduce negative emotions; 11.3 Conserving mental resources; 12.1 Restructuring the physical environment; 12.2 Restructuring the social environment; 12.3 Avoidance/reducing exposure to cues for the behaviour; 12.4 Distraction; 13.5 Identity associated with changed behaviour; 15.1 Verbal persuasion about capability; 15.2 Mental rehearsal of successful performance; 15.3 Focus on past success; 15.4 Self-talk	1.1 Goal setting (behaviour); 1.9 Commitment; 3.1 Social support (unspecified); 5.1 Information about health consequences; 5.3 Information about social and environmental consequences; 10.4 Social reward; 11.1 Pharmacological support; 11.2 Reduce negative emotions; 13.5 Identity associated with changed behaviour; 15.1 Verbal persuasion about capability; 15.3 Focus on past success

Mutter (2020)	1.1 Goal setting (behaviour); 1.2 Problem solving; 1.4 Action planning; 5.5 Anticipated regret; 9.3 Comparative imagining of future outcomes; 16.1 Imaginary punishment; 16.2 Imaginary reward	5.2 Salience of consequences; 13.3 Incompatible beliefs; 16.1 Imaginary punishment; 16.2 Imaginary reward
Naughton (2014)	1.1 Goal setting (behaviour); 1.2 Problem solving; 1.5 Review behaviour goal(s); 1.9 Commitment; 2.2 Feedback on behaviour; 3.1 Social support (unspecified); 4.2 Information about antecedents; 4.3 Re-attribution; 5.1 Information about health consequences; 5.3 Information about social and environmental consequences; 5.6 Information about emotional consequences; 6.2 Social comparison; 8.1 Behavioural practice/rehearsal; 8.2 Behaviour substitution; 10.4 Social reward; 10.9 Self-reward; 11.1 Pharmacological support; 11.2 Reduce negative emotions	None
Naughton (2017)	1.1 Goal setting (behaviour); 1.2 Problem solving; 1.4 Action planning; 1.6 Discrepancy between current behaviour and goal; 1.7 Review outcome goal(s); 1.9 Commitment; 2.2 Feedback on behaviour; 3.1 Social support (unspecified); 3.3 Social support (emotional); 4.2 Information about antecedents; 5.1 Information about health consequences; 5.2 Salience of consequences; 5.3 Information about social and environmental consequences; 5.5 Anticipated regret; 5.6 Information about emotional consequences; 6.2 Social comparison; 8.1 Behavioural practice/rehearsal; 8.2 Behaviour substitution; 8.4 Habit reversal; 9.1 Credible source; 9.2 Pros and cons; 9.3 Comparative imagining of future outcomes; 10.4 Social reward; 10.7 Self-incentive; 10.9 Self-reward; 11.2 Reduce negative emotions; 11.3 Conserving mental resources; 12.1 Restructuring the physical environment; 12.2 Restructuring the social environment; 12.3 Avoidance/reducing exposure to cues for the behaviour; 12.4 Distraction; 13.1 Identification of self-as role model; 13.2 Framing/reframing; 13.5 Identity associated with changed behaviour; 15.1 Verbal persuasion about capability; 15.2 Mental rehearsal of successful performance; 15.3 Focus on past success; 15.4 Self-talk	None
Naughton (2023)	1.1 Goal setting (behaviour); 1.2 Problem solving; 1.4 Action planning; 1.9 Commitment; 2.2 Feedback on behaviour; 2.3 Self-monitoring of behaviour; 3.1 Social support (unspecified); 3.3 Social support (emotional); 4.1 Instruction on how to perform the behaviour; 4.2 Information about antecedents; 4.3 Re-attribution; 5.1 Information about health consequences; 5.2 Salience of consequences; 5.3 Information about social and environmental consequences; 5.5 Anticipated regret; 5.6 Information about emotional consequences; 6.2 Social comparison; 6.3 Information about others' approval; 7.1 Prompts/cues; 7.8 Associative learning; 8.1 Behavioural practice/rehearsal; 8.2 Behaviour substitution; 8.3 Habit formation; 8.4 Habit reversal; 9.1 Credible source; 9.2 Pros and cons; 10.4 Social reward; 10.7 Self-incentive; 10.9 Self-reward; 11.1 Pharmacological support; 11.2 Reduce negative emotions; 11.3 Conserving mental resources; 12.1 Restructuring the physical environment; 12.2 Restructuring the social environment; 12.3 Avoidance/reducing exposure to cues for the behaviour; 12.4 Distraction; 13.1 Identification of self-as role model; 13.2 Framing/reframing; 13.4 Valued self-identify; 13.5 Identity associated with changed behaviour; 15.1 Verbal persuasion about capability; 15.2 Mental rehearsal of successful performance; 15.3 Focus on past success; 15.4 Self-talk; 16.1 Imaginary punishment; 16.2 Imaginary reward	1.1 Goal setting (behaviour); 1.2 Problem solving; 1.4 Action planning; 3.1 Social support (unspecified); 4.1 Instruction on how to perform the behaviour; 5.1 Information about health consequences; 5.3 Information about social and environmental consequences; 8.2 Behaviour substitution; 8.4 Habit reversal; 11.1 Pharmacological support; 12.1 Restructuring the physical environment; 12.4 Distraction; 13.1 Identification of self-as role model; 15.1 Verbal persuasion about capability

Palmer (2023)	1.1 Goal setting (behaviour); 3.1 Social support (unspecified); 4.1 Instruction on how to perform the behaviour; 7.7 Exposure; 7.8 Associative learning; 11.1 Pharmacological support	3.1 Social support (unspecified)
Pbert (2020)	1.1 Goal setting (behaviour); 1.2 Problem solving; 1.5 Review behaviour goal(s); 2.3 Self-monitoring of behaviour; 3.1 Social support (unspecified); 4.1 Instruction on how to perform the behaviour; 4.2 Information about antecedents; 4.3 Re-attribution; 4.4 Behavioural experiments; 5.1 Information about health consequences; 5.4 Monitoring of emotional consequences; 6.3 Information about others' approval; 7.1 Prompts/cues; 8.1 Behavioural practice/rehearsal; 8.2 Behaviour substitution; 8.7 Graded tasks; 9.1 Credible source; 9.2 Pros and cons; 11.2 Reduce negative emotions; 13.2 Framing/reframing; 15.1 Verbal persuasion about capability; 15.3 Focus on past success; 15.4 Self-talk; 16.2 Imaginary reward	1.1 Goal setting (behaviour); 3.1 Social support (unspecified); 3.2 Social support (practical); 3.3 Social support (emotional); 4.2 Information about antecedents; 5.1 Information about health consequences; 5.3 Information about social and environmental consequences; 5.6 Information about emotional consequences; 6.2 Social comparison; 8.2 Behaviour substitution; 8.3 Habit formation; 10.9 Self-reward; 11.1 Pharmacological support; 11.2 Reduce negative emotions; 12.2 Restructuring the social environment; 12.3 Avoidance/reducing exposure to cues for the behaviour; 12.4 Distraction; 15.1 Verbal persuasion about capability
Spears (2019)	1.2 Problem solving; 1.4 Action planning; 1.5 Review behaviour goal(s); 2.2 Feedback on behaviour; 2.4 Self-monitoring of outcome(s) of behaviour; 3.1 Social support (unspecified); 3.3 Social support (emotional); 4.2 Information about antecedents; 4.3 Re-attribution; 4.4 Behavioural experiments; 5.1 Information about health consequences; 5.3 Information about social and environmental consequences; 5.4 Monitoring of emotional consequences; 5.6 Information about emotional consequences; 6.3 Information about others' approval; 8.1 Behavioural practice/rehearsal; 8.2 Behaviour substitution; 8.4 Habit reversal; 10.4 Social reward; 10.7 Self-incentive; 10.9 Self-reward; 11.1 Pharmacological support; 11.2 Reduce negative emotions; 11.3 Conserving mental resources; 12.1 Restructuring the physical environment; 12.2 Restructuring the social environment; 12.3 Avoidance/reducing exposure to cues for the behaviour; 12.4 Distraction; 13.1 Identification of self-as role model; 13.2 Framing/reframing; 13.4 Valued self-identify; 13.5 Identity associated with changed behaviour; 15.1 Verbal persuasion about capability; 15.3 Focus on past success; 15.4 Self-talk; 16.2 Imaginary reward	1.1 Goal setting (behaviour); 1.2 Problem solving; 1.4 Action planning; 1.5 Review behaviour goal(s); 2.4 Self-monitoring of outcome(s) of behaviour; 3.1 Social support (unspecified); 4.2 Information about antecedents; 4.3 Re-attribution; 8.2 Behaviour substitution; 8.4 Habit reversal; 10.4 Social reward; 11.1 Pharmacological support; 11.2 Reduce negative emotions; 11.3 Conserving mental resources; 12.1 Restructuring the physical environment; 12.2 Restructuring the social environment; 12.3 Avoidance/reducing exposure to cues for the behaviour; 12.4 Distraction; 13.2 Framing/reframing
Sridharan (2019)	1.1 Goal setting (behaviour); 1.2 Problem solving; 1.4 Action planning; 2.2 Feedback on behaviour; 2.3 Self-monitoring of behaviour; 3.1 Social support (unspecified); 4.2 Information about antecedents; 4.3 Re-attribution; 5.6 Information about emotional consequences; 6.2 Social comparison; 8.2 Behaviour substitution; 9.1 Credible source; 10.4 Social reward; 11.1 Pharmacological support; 11.2 Reduce negative emotions; 13.2 Framing/reframing; 13.4 Valued self-identify	1.1 Goal setting (behaviour); 1.4 Action planning; 2.2 Feedback on behaviour; 2.3 Self-monitoring of behaviour; 3.1 Social support (unspecified); 10.4 Social reward; 11.1 Pharmacological support; 11.2 Reduce negative emotions; 13.4 Valued self-identify

Stoops (2009)	1.1 Goal setting (behaviour); 2.6 Biofeedback; 4.1 Instruction on how to perform the behaviour; 6.1 Demonstration of the behaviour; 8.7 Graded tasks; 10.1 Material incentive (behaviour); 10.2 Material reward (behaviour); 14.3 Remove reward; 14.4 Reward approximation; 14.9 Reduce reward frequency	1.1 Goal setting (behaviour); 2.6 Biofeedback; 4.1 Instruction on how to perform the behaviour; 6.1 Demonstration of the behaviour; 8.7 Graded tasks
Tucker (2021)	1.1 Goal setting (behaviour); 1.2 Problem solving; 1.4 Action planning; 3.1 Social support (unspecified); 4.1 Instruction on how to perform the behaviour; 4.2 Information about antecedents; 5.1 Information about health consequences; 5.2 Salience of consequences; 5.3 Information about social and environmental consequences; 5.6 Information about emotional consequences; 6.2 Social comparison; 6.3 Information about others' approval; 8.2 Behaviour substitution; 10.9 Self-reward; 11.1 Pharmacological support; 11.2 Reduce negative emotions; 11.3 Conserving mental resources; 12.2 Restructuring the social environment; 12.3 Avoidance/reducing exposure to cues for the behaviour; 12.4 Distraction; 13.1 Identification of self-as role model; 13.4 Valued self-identify; 13.5 Identity associated with changed behaviour; 15.1 Verbal persuasion about capability; 15.3 Focus on past success; 16.2 Imaginary reward	None
Vilardaga (2020)	1.1 Goal setting (behaviour); 1.2 Problem solving; 2.2 Feedback on behaviour; 2.3 Self-monitoring of behaviour; 3.1 Social support (unspecified); 4.1 Instruction on how to perform the behaviour; 4.2 Information about antecedents; 5.1 Information about health consequences; 5.2 Salience of consequences; 5.3 Information about social and environmental consequences; 5.4 Monitoring of emotional consequences; 5.6 Information about emotional consequences; 7.1 Prompts/cues; 7.8 Associative learning; 8.1 Behavioural practice/rehearsal; 10.4 Social reward; 11.1 Pharmacological support; 11.2 Reduce negative emotions; 13.2 Framing/reframing; 13.4 Valued self-identify; 15.1 Verbal persuasion about capability	1.1 Goal setting (behaviour); 1.2 Problem solving; 2.2 Feedback on behaviour; 2.3 Self-monitoring of behaviour; 3.1 Social support (unspecified); 3.2 Social support (practical); 3.3 Social support (emotional); 4.2 Information about antecedents; 5.6 Information about emotional consequences; 7.1 Prompts/cues; 8.2 Behaviour substitution; 10.4 Social reward; 10.9 Self-reward; 11.1 Pharmacological support; 11.2 Reduce negative emotions; 12.3 Avoidance/reducing exposure to cues for the behaviour; 15.3 Focus on past success
Villanti (2022)	1.1 Goal setting (behaviour); 1.2 Problem solving; 1.4 Action planning; 1.9 Commitment; 2.2 Feedback on behaviour; 2.3 Self-monitoring of behaviour; 3.1 Social support (unspecified); 3.2 Social support (practical); 3.3 Social support (emotional); 4.1 Instruction on how to perform the behaviour; 4.2 Information about antecedents; 4.4 Behavioural experiments; 5.1 Information about health consequences; 5.2 Salience of consequences; 5.3 Information about social and environmental consequences; 5.6 Information about emotional consequences; 6.2 Social comparison; 7.1 Prompts/cues; 8.1 Behavioural practice/rehearsal; 8.2 Behaviour substitution; 8.4 Habit reversal; 8.7 Graded tasks; 9.1 Credible source; 10.4 Social reward; 11.1 Pharmacological support; 11.2 Reduce negative emotions; 11.3 Conserving mental resources; 12.1 Restructuring the physical environment; 12.2 Restructuring the social environment; 12.3 Avoidance/reducing exposure to cues for the behaviour; 12.4 Distraction; 12.5 Adding objects to the environment; 13.4 Valued self-identify; 15.1 Verbal persuasion about capability; 16.2 Imaginary reward	None

Walker (2020)	3.1 Social support (unspecified); 4.1 Instruction on how to perform the behaviour; 5.1 Information about health consequences; 5.2 Salience of consequences; 5.3 Information about social and environmental consequences; 5.5 Anticipated regret; 9.3 Comparative imagining of future outcomes	3.1 Social support (unspecified); 4.1 Instruction on how to perform the behaviour; 5.1 Information about health consequences
White (2019)	1.1 Goal setting (behaviour); 1.2 Problem solving; 1.4 Action planning; 1.5 Review behaviour goal(s); 1.6 Discrepancy between current behaviour and goal; 1.9 Commitment; 3.1 Social support (unspecified); 3.3 Social support (emotional); 4.2 Information about antecedents; 4.3 Re-attribution; 5.1 Information about health consequences; 5.3 Information about social and environmental consequences; 5.4 Monitoring of emotional consequences; 5.6 Information about emotional consequences; 6.3 Information about others' approval; 8.2 Behaviour substitution; 9.2 Pros and cons; 10.9 Self-reward; 11.1 Pharmacological support; 11.2 Reduce negative emotions; 11.3 Conserving mental resources; 12.1 Restructuring the physical environment; 12.2 Restructuring the social environment; 12.3 Avoidance/reducing exposure to cues for the behaviour; 12.4 Distraction; 13.2 Framing/reframing; 13.5 Identity associated with changed behaviour; 15.1 Verbal persuasion about capability; 15.3 Focus on past success; 15.4 Self-talk	1.1 Goal setting (behaviour); 1.2 Problem solving; 1.4 Action planning; 3.1 Social support (unspecified); 3.3 Social support (emotional); 4.2 Information about antecedents; 5.1 Information about health consequences; 5.3 Information about social and environmental consequences; 5.4 Monitoring of emotional consequences; 5.6 Information about emotional consequences; 6.3 Information about others' approval; 8.2 Behaviour substitution; 9.2 Pros and cons; 10.9 Self-reward; 11.1 Pharmacological support; 11.2 Reduce negative emotions; 11.3 Conserving mental resources; 12.1 Restructuring the physical environment; 12.2 Restructuring the social environment; 12.3 Avoidance/reducing exposure to cues for the behaviour; 12.4 Distraction; 13.5 Identity associated with changed behaviour; 15.1 Verbal persuasion about capability; 15.3 Focus on past success

G.H. Number of BCTs in Intervention and Control Conditions

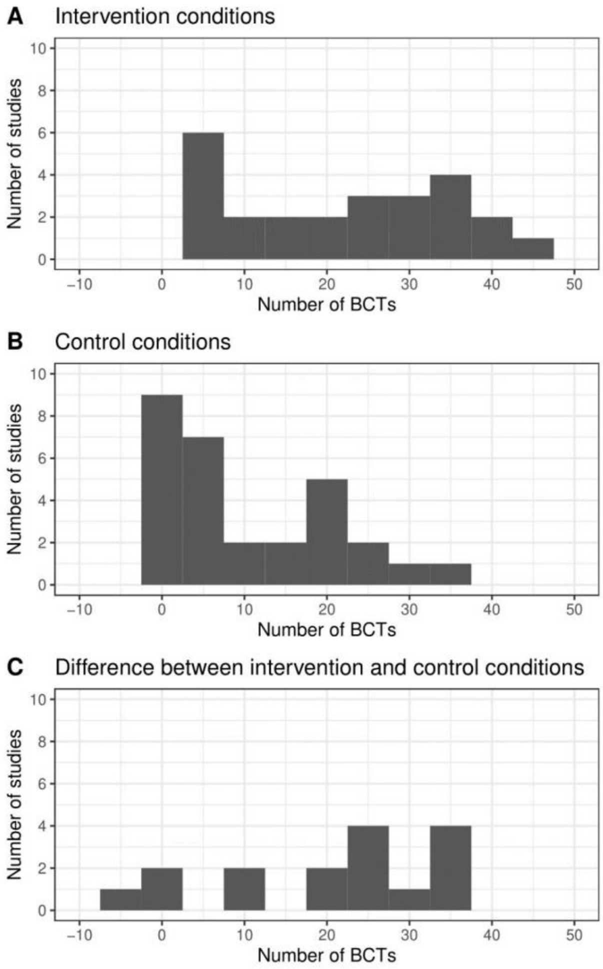

Supplementary Figure 2 Histograms showing the distribution of the number of BCTs in the intervention (A) and control (B) conditions as well as the number of BCTs in the intervention condition subtracted by the number of BCTs in the control condition (C) for the 29 included studies for which we have sufficient materials to characterise intervention content

~~H.~~I. Publication bias

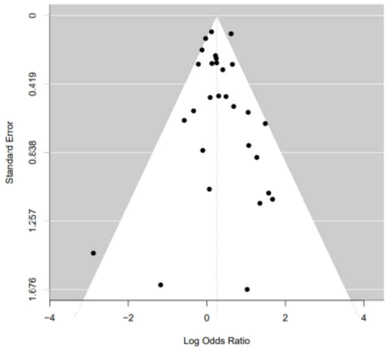

Figure 2 Funnel plot showing standard error by effect size for the 29 studies for which we have sufficient materials to characterise the intervention in terms of content and delivery features Asymmetry or excessive data points outside the white funnel/pyramid would indicate publication bias.

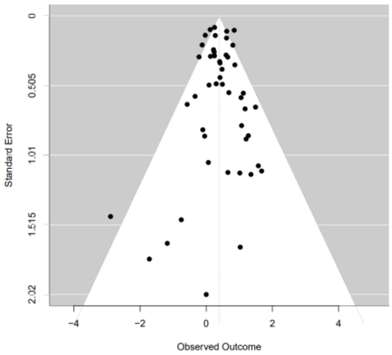

Figure 3 Funnel plot showing standard error by effect size for the 48 studies included in the extended analysis. Asymmetry or excessive data points outside the white funnel/pyramid would indicate publication bias.

~~I.~~J. Details of meta-CART analysis: initial trees and pruning/cross-validation tables

Main Analysis

Supplementary Table 4 Initial, unpruned tree and results of ten-fold cross-validation of meta-CART analyses including all 55 effect sizes

Level	Between subgroups Q statistic (Q_b)	Residual heterogeneity (τ^2)	Moderator	Split point	Parent node	Cross-validated sum of squared errors	Standard deviation of cross-validated sum of squared errors

1	0.00	0.102	NA	NA	NA	1.014	0.215
2	7.16	0.054	BCT 1.9 “Commitment”	< 0.5	1	1.157	0.256
3	11.99	0.044	SEP	Low	3	1.152	0.279
4	17.57	0.031	BCT 11.3 “Conserving mental resources”	< 0.5	4	1.337	0.291
5	20.20	0.027	BCT 6.2 “Social Comparison”	< 0.5	2	1.402	0.298
6	22.35	0.026	Mode of Delivery	Mobile based	9	1.374	0.296

Tree using only effect sizes for low SEP populations

Supplementary Table 5 Initial, unpruned tree and results of ten-fold cross-validation of meta-CART analyses including the 28 effect sizes for low SEP populations

Level	Between subgroups Q statistic (Q_b)	Residual heterogeneity (τ^2)	Moderator	Split point	Parent node	Cross-validated sum of squared errors	Standard deviation of cross-validated sum of squared errors
1	0.00	0.024	NA	NA	NA	1.029	0.384
2	7.82	0.000	BCT 2.3 “Self-monitoring of behaviour”	< 0.5	1	1.041	0.398
3	11.72	0.000	BCT 2.2 “Feedback on behaviour”	< 0.5	3	1.227	0.477
4	15.52	0.000	BCT 9.1 “Credible source”	< 0.5	2	1.200	0.444
5	18.48	0.000	BCT 1.8 “Behavioural contract”	< 0.5	7	1.428	0.513
6	19.67	0.000	BCT 5.5 “Anticipated regret”	< 0.5	6	1.459	0.503

Tree using only effect sizes for high SEP populations

Supplementary Table 6 Initial, unpruned tree and results of ten-fold cross-validation of meta-CART analyses including the 27 effect sizes for high SEP populations

Level	Between subgroups Q statistic (Q_b)	Residual heterogeneity (τ^2)	Moderator	Split point	Parent node	Cross-validated sum of squared errors	Standard deviation of cross-validated sum of squared errors
1	0.00	0.173	NA	NA	NA	1.025	0.243

2	10.95	0.055	BCT 1.9 “Commitment”	<0.5	1	0.976	0.231
3	20.73	0.026	BCT 12.4 “Distraction”	<0.5	2	1.056	0.237
4	27.70	0.010	BCT 16.1 “Imaginary punishment”	<0.5	4	1.048	0.243

J.K. Sensitivity analyses

Coding BCTs differently for exploratory meta-regressions

Univariate meta-regressions controlled for false discovery rate using the Benjamini-Hochberg correction (Benjamini & Hochberg, 1995).

When dropping all observations which had a BCT that was present in the control, but not the intervention condition, univariate meta-regressions suggested that only the BCT “Commitment” (b=0.47, 95% CI: 0.25-0.70, p<0.001) was significantly associated with overall effect size. When running univariate meta-regressions on low SES effect sizes, no moderators emerged as significant. The association between the BCT “Commitment” and effectiveness was non-significantly positive, however (b=0.10, 95% CI: -0.23-0.43, p=0.558). When running univariate meta-regressions on high SES effect sizes, no moderators emerged as significant either. The association between the BCT “Commitment” and effectiveness was significantly positive when not controlling for false discover rate, however (b=0.82, 95% CI: 0.22-1.30, p=0.001). Therefore, multivariate meta-regressions were not warranted.

When entering BCTs as a binary variable by recoding all -1 cases were recoded to 0s, therefore treating cases where a BCT was present in the control, but not the intervention condition in the same way as cases where the BCT was present in both or neither conditions, univariate meta-regressions suggested that the BCTs “Commitment” (b=0.49, 95% CI: 0.27-0.71, p<0.001) and “Credible Source” (b=-0.38, 95% CI: -0.61- -0.15, p=0.001) were significantly associated with effectiveness. When entering both of these moderators into a multivariate meta-regression, only “Commitment” significantly associated with effectiveness (b=0.39, 95% CI: 0.09-0.69, p=0.012), while “Credible Source” was attenuated (b=-0.14, 95% CI: -0.43-0.15, p=0.329). When running univariate meta-regressions on low SES effect sizes, no moderators emerged as significant. The association between the BCT “Commitment” and effectiveness was significantly positive when not controlling for false discover rate, however (b=0.49, 95% CI: 0.27-0.72, p<0.001). When running univariate meta-regressions on high SES effect sizes, only the BCT “Commitment” (b=0.79, 95% CI: 0.35-1.24, p=0.001) was significantly associated with effectiveness. A multivariate meta-regression was therefore not warranted for either high or low SES effect sizes.
